# Regulated interaction of ID2 with the anaphase-promoting complex links progression through mitosis with reactivation of cell-type-specific transcription

**DOI:** 10.1038/s41467-022-29502-2

**Published:** 2022-04-19

**Authors:** Sang Bae Lee, Luciano Garofano, Aram Ko, Fulvio D’Angelo, Brulinda Frangaj, Danika Sommer, Qiwen Gan, KyeongJin Kim, Timothy Cardozo, Antonio Iavarone, Anna Lasorella

**Affiliations:** 1 Institute for Cancer Genetics, Columbia University Medical Center, New York, 10032 USA; 2 Division of Life Sciences, Jeonbuk National University, Jeonju, 54896 Republic of Korea; 3 Department of Biomedical Sciences, College of Medicine, Inha University, Incheon, Republic of Korea; 4 Department of Biochemistry and Molecular Pharmacology, New York University Grossman School of Medicine, NYU Langone Health, New York, NY 10016 USA; 5 Department of Pathology and Cell Biology, Columbia University Medical Center, New York, 10032 USA; 6 Department of Neurology, Columbia University Medical Center, New York, 10032 USA; 7 Herbert Irving Comprehensive Cancer Center, Columbia University Medical Center, New York, 10032 USA; 8 Department of Pediatrics, Columbia University Medical Center, New York, 10032 USA

**Keywords:** Ubiquitylated proteins, Transcriptomics

## Abstract

Tissue-specific transcriptional activity is silenced in mitotic cells but it remains unclear whether the mitotic regulatory machinery interacts with tissue-specific transcriptional programs. We show that such cross-talk involves the controlled interaction between core subunits of the anaphase-promoting complex (APC) and the ID2 substrate. The N-terminus of ID2 is independently and structurally compatible with a pocket composed of core APC/C subunits that may optimally orient ID2 onto the APC^CDH1^ complex. Phosphorylation of serine-5 by CDK1 prevented the association of ID2 with core APC, impaired ubiquitylation and stabilized ID2 protein at the mitosis-G1 transition leading to inhibition of basic Helix-Loop-Helix (bHLH)-mediated transcription. The serine-5 phospho-mimetic mutant of ID2 that inefficiently bound core APC remained stable during mitosis, delayed exit from mitosis and reloading of bHLH transcription factors on chromatin. It also locked cells into a “mitotic stem cell” transcriptional state resembling the pluripotent program of embryonic stem cells. The substrates of APC^CDH1^ SKP2 and Cyclin B1 share with ID2 the phosphorylation-dependent, D-box-independent interaction with core APC. These results reveal a new layer of control of the mechanism by which substrates are recognized by APC.

## Abstract (web summary)

Tissue-specific transcriptional activity is silenced in mitotic cells. Here the authors reveal a general phosphorylation-dependent mechanism of recognition for the anaphase-promoting complex (APC) substrates, and show that the APC targets ID2 during the establishment of post-mitotic transcription.

## Introduction

The progression of the eukaryotic mitotic cycle is controlled by ordered oscillations of cell cycle regulators^1,2^. Ubiquitin-mediated proteolysis underpins this process, which is mostly controlled by the multi-subunit ubiquitin ligase anaphase promoting complex/cyclosome (APC/C, APC hereafter)^3–8^. The activity of APC is modulated during mitosis by the structurally related CDC20/Fizzy and CDH1/FZR1 coactivators that induce conformational changes in the APC complex^9^. Temporal control of the binding of these two coactivators to APC (APC^CDC20^, APC^CDH1^) and targeting of distinct substrates ensures metaphase to anaphase transition, exit from mitosis and entry into the G1 phase of the cell cycle, respectively^10–16^. A characteristic feature of cells undergoing mitosis is the drastic down-regulation of transcription^17–21^. In spite of the global decrease of gene expression during mitosis, proliferating cells are able to transfer transcriptional programs from mother to daughter cell and preserve their cell identity. As cells exit mitosis and enter G1, the amplitude of gene expression is reestablished through sequential transcription waves that re-institute cell-type-specific functions^17,18,20^. It was reported that in embryonic stem cells, APC controls gene expression through an interaction with WDR5 at promoters of pluripotency factors, decorating histones with K11/K48-branched chains leading to proteasome-mediated histone degradation and rapid expression of pluripotency factors after mitosis^22^. However, it remains incompletely understood whether and how APC substrates orchestrate the cross-talk between cell division and transcriptional programs of cell identity. This is especially relevant as APC activated by CDH1 orchestrates mitotic exit and the initiation of G1-specific differentiation programs^23^. One of the substrates of APC^CDH1^ is the Inhibitor of Differentiation 2, ID2 protein^24^. ID2 belongs to the evolutionary conserved ID family of transcription regulators that function to inhibit binding to DNA and activity of bHLH transcription factors, key determinants of cell fate determination and differentiation in multiple tissue types^25–29^. We showed that ID2 is recruited by CDH1 through the D-box located within the ID2 C-terminal region and is targeted for degradation by APC. We also determined that in neuronal cells degradation of ID2 by APC^CDH1^ is maximally achieved as differentiation is completed^24^. However, it remains unknown whether there is a requirement for temporal APC-mediated regulation of ID2 during the cell cycle, in particular at mitosis-G1 transition when expression of cell identity genes undergoes rapid changes. Here, we show that the N-terminal region of ID2 and the phosphorylation state of ID2-Ser-5 regulates ID2 binding to a pocket composed of core APC subunits and this interaction is essential for optimal positioning of ID2 onto the APC^CDH1^ complex. Ser-5 phosphorylated ID2 is unable to bind APC in mitosis and is stabilized. As cells exit mitosis, ID2 is dephosphorylated, recruited by APC and targeted for ubiquitin-mediated proteasomal degradation. The dynamic changes of the association of ID2 with APC affect chromatin binding of bHLH transcription factors and transcriptional activity at mitosis-G1 transition, thus suggesting a regulatory role for ID2 in the transcriptional adjustment of distinct cellular programs after mitosis.

## Results

### Distinct domains of ID2 bind core APC and the CDH1 coactivator

**Fig. 1:**
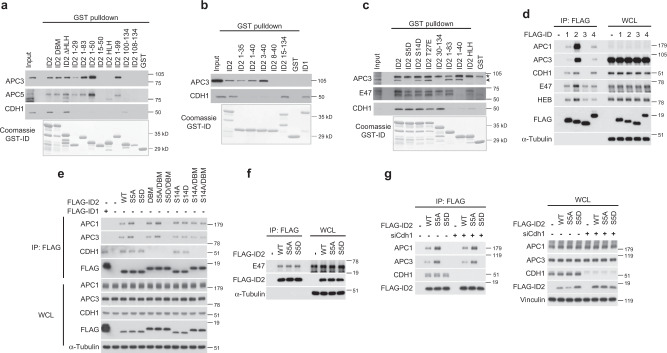
The N-terminal region of ID2 including Ser-5 is necessary for the interaction between ID2 and core APC subunits. **a** GST-ID2 WT and deletion mutants of the N- and C-terminus were incubated with lysates from HeLa cells. Bound proteins were analyzed by western blot. Lower panel, Coomassie staining of GST-ID2 proteins used in the GST pull-down. **b** GST pull-down using GST-ID2 WT and N-terminus deletion mutants and HeLa cell lysates. Bound proteins were analyzed by western blot. Lower panel, Coomassie staining of GST-ID2 proteins used in the GST pull-down. **c** GST pull-down using GST-ID2 proteins and U-2 OS cell lysates followed by western blot. Arrowhead, specific band; asterisk, non-specific band. Lower panel, Coomassie staining of GST-ID2 proteins used in the GST pull-down. **d** In vivo interaction between ID family members expressed in HeLa cells and core APC subunits, CDH1 coactivator, and bHLH transcription factors (E47 or HEB). ID interacting proteins were detected by FLAG immunoprecipitation followed by western blot for endogenous proteins as indicated. **e** Interaction between ID2 phosphorylation mutants and D-Box mutant (DBM) expressed in HeLa cells and core APC subunits or CDH1 coactivator. ID2 interacting proteins were detected by FLAG immunoprecipitation followed by western blot for endogenous proteins as indicated. **f** FLAG-ID2-S5 phospho-mutants expressed in HeLa cells were immunoprecipitated and probed by western blot for the association with endogenous E47. **g** HeLa cells transduced with non-targeting or *CDH1* siRNA were transfected with FLAG-ID2 WT or ID2-S5 phospho-mutants. Cellular lysates were used in FLAG immunoprecipitation followed by western blot for core APC subunits and CDH1 (left panel). Right panel, whole cellular lysates. Molecular weight markers are indicated in kDa. Coomassie staining in **a**–**c** was performed on SDS-gels loaded with the same GST-fusion protein amounts used in the binding reactions. APC proteins and CDH1 are from the same blots in each panel; E47 and HEB in panel **d** are from two independent gels; FLAG is from an independent gel; Loading controls are from the same gel as HEB in **d**, FLAG in **e**, E47 in **f**, APC1/APC3/CDH1 in **g**. Experiments were repeated two times with similar results.

We reported that the D-box of ID2 mediates the interaction with the CDH1 co-activator of APC, but not the core APC complex^24^. As both regulation and functional consequences of the binding between core APC and ID2 remain to be charted, here we sought to decipher the significance of this interaction. To identify the domain of ID2 that mediates the interaction with core APC, we generated deletion mutants of the ID2 protein and expressed GST-ID2 polypeptides in bacteria as purified GST fusion proteins (Supplementary Fig. 1a). The N-terminal region of ID2 (amino acids 1–50) retained full ability to bind core APC as documented by GST pulldown after incubation of different GST-ID2 polypeptides with HeLa cell lysate and western blot for the APC core subunits APC3 or APC5 (Fig. 1a). However, the ID2 polypeptide including amino acids 15–50 failed to bind either APC3 and APC5, revealing that the first 15 amino acids of ID2 are essential for recruitment of core APC. C-terminal deletions extending up to amino acid 29 (GST-ID2 1–29) did not eliminate binding to core APC. Conversely, the interaction of ID2 with the CDH1 coactivator was strictly dependent on the presence of the C-terminally located D-box at residues 100–104, as shown by successful binding to CDH1 only of the Id2-ΔHLH and ID2 100–134 polypeptides, both of which retain an intact D-box but not the deletion mutants that lack amino acids 100–104, regardless of their binding to core APC (Fig. 1a, Supplementary Fig. 1a). Thus, the ID2 protein exhibits a dual capacity to capture the APC^CDH1^ holocomplex whereby the N-terminal region captures core APC whereas the C-terminal D-box is independently recruited by CDH1.

Next, we performed detailed N-terminal amino acid deletions within the first 40 residues of ID2 to identify the N-terminal residues essential for the ID2-core APC interaction. Surprisingly, a minimal deletion of 3 amino acids was tolerated for core APC binding (ID2 3–40) whereas deletion of the first 8 amino acids of ID2 (ID2 8–40) abolished the interaction with core APC (Fig. 1b). Despite sharing with ID2 the C-terminal D-box and the interaction with CDH1^24^, the ID family member ID1 exhibits divergent amino acid sequences at the N-terminal domain (Supplementary Fig. 1b). Consistent with the notion that the N-terminal residues of ID2 are essential for binding core APC, ID1 was unable to bind core APC in vitro (Fig. 1b).

We recently found that the N-terminal region of ID2 can be phosphorylated on three residues, serine-5, serine-14, and threonine-27^30^. We therefore asked whether phosphorylation of any of the three amino acids, modeled through the phospho-mimetic mutations of serine-5 to aspartic acid (S5D), serine-14 to aspartic acid (S14D) and threonine-27 to glutamic acid (T27E), might impact the ability of full-length ID2 to bind core APC and CDH1. Whereas CDH1 binding was preserved in ID2 harboring each of the three phospho-mimetic mutations, binding to core APC was disrupted by mutation of the evolutionarily conserved S5 (ID2-S5D), but not ID2-S14D or ID2-T27E (Fig. 1c, Supplementary Fig. 1c). None of the ID2 phospho-mimetic mutants affected the interaction with the ubiquitous bHLH transcription factor E47, which is mediated by the HLH domain (amino acids 35–76) of ID2 (Fig. 1c). These findings underscore the specificity of the binding of the N-terminal region of ID2 to core APC and the potential regulation of this interaction by S5 phosphorylation.

To test whether the mechanisms uncovered in vitro also operate to regulate the interaction of ID2 with core APC and CDH1 in vivo, we expressed the wild type FLAG-ID proteins (ID1, ID2, ID3, ID4) and multiple mutants of FLAG-ID2 in HeLa cells and evaluated the ability of the FLAG-ID proteins to co-immunoprecipitate core APC and CDH1. In this system, wild type FLAG-ID2 but not FLAG-ID1 or FLAG-ID4 bound efficiently to core APC, as determined by FLAG immunoprecipitation followed by western blot for APC1 and APC3. FLAG-ID1, FLAG-ID2, and FLAG-ID4 exhibited similar binding to CDH1 (Fig. 1d). FLAG-ID3, which lacks a D-box and is not targeted by APC^CDH1^ for ubiquitin-mediated proteasomal degradation^24^, failed to bind either core APC or CDH1. As expected, all ID proteins co-immunoprecipitated the endogenous bHLH transcription factors E47 and HEB (Fig. 1d). Mirroring the in vitro findings, the ID2-S5D phospho-mimetic mutant protein failed to bind core APC subunits but retained binding to CDH1 (Fig. 1e). Conversely, the ID2 protein harboring a mutated D-box (ID2-DBM) bound to core APC but not CDH1, therefore confirming that the C-terminal D-box and the N-terminal S5-containing domain of ID2 differentially capture CDH1 and core APC, respectively. The ID2-S5A phospho-mutant and both ID2-S14A phospho-mutant and ID2-S14D phospho-mimetic proteins interacted with core APC and CDH1 as efficiently as wild-type ID2 (Fig. 1e). Binding to E47 was unaffected by S5A and S5D mutations, confirming in vivo the specificity of ID2-S5 phosphorylation as the mechanism for the regulation of the interaction with core APC (Fig. 1f).

To unequivocally demonstrate that the interaction of ID2 with CDH1 does not influence the binding of core APC to the S5-containing N-terminal region, we silenced CDH1 and immunoprecipitated the FLAG–ID2–APC complex. Loss of CDH1 did not affect the ability of wild type and S5A FLAG-ID2 to co-precipitate core APC whereas the ID2-S5D phospho-mimetic mutant was defective in APC binding regardless of whether CDH1 was present or absent in HeLa cell lysate (Fig. 1g).

**Fig. 2:**
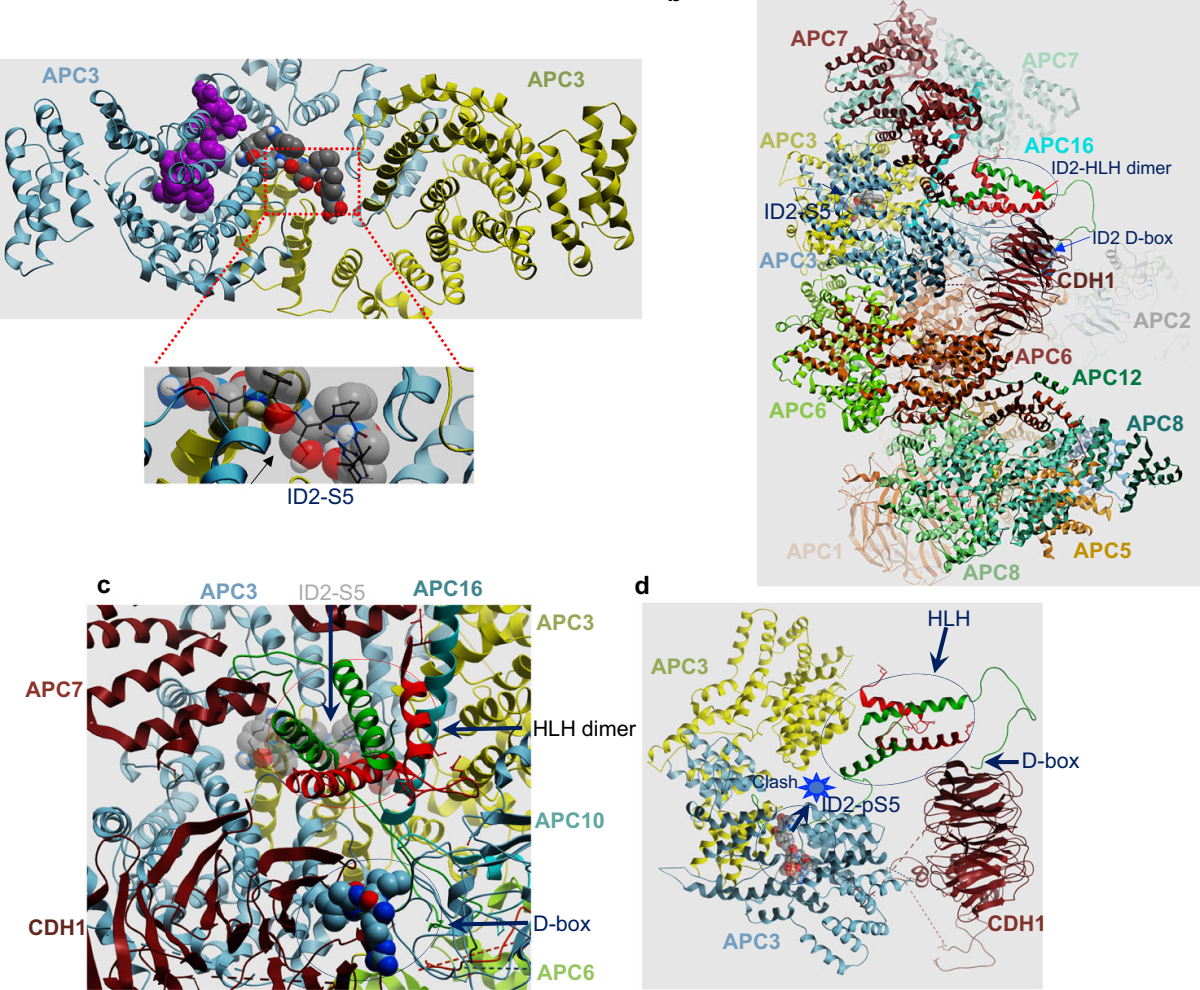
Molecular docking of ID2 on the APC^CDH1^ complex. **a** Upper panel, docked locations of ID2 amino acids 2-8 (gray-carbon, red-oxygen, blue-nitrogen, white-proton spheres) and ID2 phosphoS5 amino acids 2-8 (purple spheres) on the APC3 homodimer (gray, chain **a**; yellow, chain **b**). Lower panel, close-up view of S5 showing tight packing around the serine hydroxyl that cannot accommodate an aspartate or phosphorylation. **b** and **c** Side (**b**) and close-up (**c**) views of APC^CDH1^ holocomplex with ID2 in place. The APC cavity fitting ID2 bounded by APC7 (maroon helices upper left), APC16 (long aqua helix), APC10 (gray ribbon lower right) and CDH1 (maroon beta-propeller). The ID2 D-box binding peptide is shown in solid gray (carbon), red (oxygen) and blue (nitrogen) spheres, while the ID2 N-terminal peptide containing S5 docked to APC3 is shown in transparent spheres with the same color scheme. The ID2 protein is shown as green/red ribbons representing the four-helix bundle ID2 homodimer occupying the cavity with N-terminus bound to APC3, the two central helices and the C-terminal D-box bound to CDH1. APC3 homodimer is shown at the bottom of the cavity as gray and yellow ribbon. **d** Side view of APC3 and CDH1 only with ID2 HLH homodimer. The ID2-D-box is bound to CDH1 and the docked location of ID2-phospho-S5 amino acids 2–8 is shown as space filling spheres. Location of obstruction where the distance is too close to accommodate ID2 amino acids 9–29 is labeled “clash”.

**Fig. 3:**
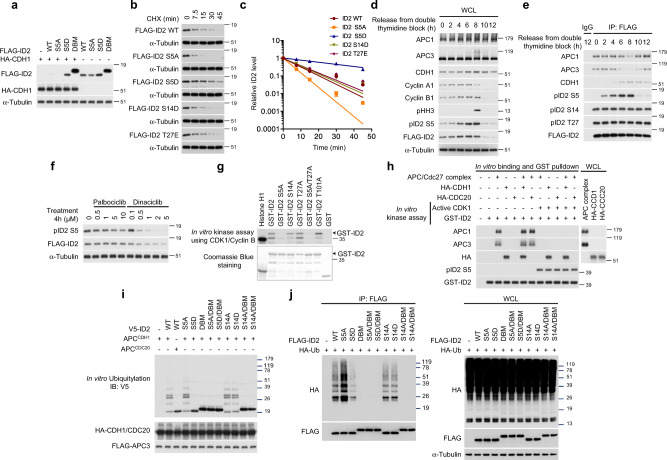
ID2-S5 is phosphorylated by CDK1 during mitosis leading to increased stability. **a** Overexpression of CDH1 does not affect stability of ID2 S5D. **b** Half-life of ID2 WT and phospho-mutants in HeLa cells treated with CHX. **c** Quantification of data in (**b**); data are means ± SEM of two independent experiments. **d** Regulation of ID2-S5 phosphorylation during cell cycle progression. Western blot of HeLa cells exposed to double thymidine block. **e** Loss of interaction between phosphorylated ID2-S5 and core APC subunits at entry into mitosis. Western blot of cells expressing FLAG-ID2 WT and phospho-mutants exposed to double thymidine block. **f** Inhibition of ID2-S5 phosphorylation by the CDK1-specific inhibitor dinaciclib. Western blot of lysates of cells expressing FLAG-ID2 and treated with palbociclib or dinaciclib. **g** In vitro kinase assay using CDK1-Cyclin B and GST-ID2 proteins. Lower panel, Coomassie blue staining of GST-ID2 proteins used in the assay. Arrowheads indicate full-length proteins. **h** ID2 phosphorylation by CDK1-Cyclin B prevents the interaction with core APC but not CDH1. GST-ID2 was phosphorylated in vitro by CDK1-Cyclin B before binding with FLAG-APC3 immunopurified from HeLa cells and activated by in vitro translated HA-CDC20 or CDH1. GST-pull-down reactions were analyzed by western blot. **i** Loss of ubiquitylation by the ID2-S5D. In vitro ubiquitylation of V5-ID2 WT or mutants was performed using the APC complex prepared as in (**h**). **j** In vivo ubiquitylation in HeLa cells co-expressing FLAG-ID2 WT or mutants and HA-ubiquitin. Left panel, HA-ubiquitin western blot of FLAG-ID2 immunoprecipitates. Right panel, western blot of whole cellular lysates (WCL). Coomassie staining in g was performed on SDS-gel loaded with the same amounts of GST-fusion proteins used in the binding reactions. APC proteins and CDH1 are from the same blots in each panel; Cyclin A1, Cyclin B1, pS5-ID2, pS14-ID2 and pT27-ID2, FLAG are from independent gels; in **i**, V5 and HA/FLAG are from two independent gels; in **j**, HA and FLAG are from independent gels. Loading controls are from the same gel as FLAG. Molecular weight markers are indicated in kDa. Experiments were repeated three times for **a**–**g** and two times for **h**–**j** with similar results.

We explored the mechanism of the binding of ID2 to core APC and CDH1 by computational molecular docking. A N-terminally derived ID2 peptide spanning the S5 phosphorylation site (amino acids 2–8) exhibited a strong propensity to dock in a pocket between the two APC3 monomers in high-resolution crystal structure of the APC3 homodimer (Fig. 2a). We did not detect alternative docking sites in other CDH1-proximal, APC subunits. However, these structures are of suboptimal experimental resolution. Next, we built a model of the ID2 polypeptide (amino acids 2–106) including the N-terminal region, the dimerizing HLH domain, and C-terminal D-box docked onto the cryo-EM structure of the APC^CDH1^ holocomplex. In this model, the N-terminal ID2-S5-containing peptide positioned within the APC3 homodimer pocket with geometry, space, and energy consistent with the four-helix ID2 HLH homodimer nestling alongside the CDH1 beta-propeller domain and the C-terminal D-box bound to the canonical CDH1 D-box-binding site (Fig. 2b, c, Supplementary Fig. 2). ID2 occupies a conspicuously large cavity in the APC^CDH1^ complex, the walls of which are formed by CDH1, APC7, APC10, and APC16 and the floor is formed by APC3. This cavity accommodates the N-terminal ID2-S5-containing peptide docked within the APC3 homodimer pocket as well as the four-helix ID2 HLH homodimer nestling alongside the CDH1 beta-propeller domain and the C-terminal D-box bound to the canonical CDH1 D-box binding site as a single chain with no significant clashes (Fig. 2b, c, Supplementary Fig. 2). In this conformation, the S5 side-chain is tightly packed into a relatively hydrophobic space that cannot spatially or electrostatically accommodate phosphorylation. Upon docking the same peptide with phosphorylated S5 (ID2-phospho-S5-amino acids 2–8) or mutated to aspartic acid (D), the phosphopeptide positioned to a different location enclosed entirely within one APC3 monomer (Fig. 2a). In this conformation, the linker connecting S5 to the ID2 HLH domain (ID2 amino acids 9–29) is too large to fit into the intervening space and thus is incompatible with the 3D structure of the APC^CDH1^ holocomplex (Fig. 2d). To test the requirements of the APC3 pocket for the interaction with ID2 in accordance to the APC3-ID2 (amino acid 2–8) modeling, we generated mutant of APC3-Y21 to F/W, APC3-D81 to G/H, and APC3-L120 to R/V, amino acids that exhibit close contact with the ID2 peptide (Supplementary Fig. 3a–d). We performed binding of in vitro translated APC3 wild type and mutant with recombinant GST-ID2 by GST pull down. We included CDK1 phosphorylated GST-ID2 as control for loss of APC3-ID2 binding (see Fig. 3, Supplementary Fig. 4c for CDK1-mediated phosphorylation of ID2-S5). We found that all APC3 mutants abolished the binding to ID2 in this assay (Supplementary Fig. 3e). However, the same mutations did not alter APC3 homodimerization, that is the interaction between in vitro translated HA-APC3 wild type and mutant polypeptides with FLAG-APC3 expressed in HeLa cells (Supplementary Fig. 3f). Similarly, the interaction between in vitro translated HA-APC3 mutants with endogenous APC1 from HeLa cells was unaffected (Supplementary Fig. 3g).

Taken together, our in vitro and in vivo experiments, corroborated by the computational molecular docking of ID2 within the APC^CDH1^ holocomplex, converge on the ability of ID2 to capture core APC and CDH1 through two different regions. Phosphorylation of S5 in the N-terminal region of ID2 emerged as an event that could potentially regulate the interaction with core APC.

### Cdk1-mediated phosphorylation of serine 5 of ID2 in mitosis regulates ubiquitylation and destruction by APC

The binding of core APC and CDH1 to separate regions of ID2 suggests that efficient APC^CDH1^-mediated degradation of ID2 may require cooperative recruitment of different APC components by the N-terminal and C-terminal domains of ID2, respectively. Under this scenario, the failure to recruit core APC by the ID2-S5D phospho-mimetic mutant might result in inefficient degradation. As the enzymatic ubiquitin ligase activity of the APC holocomplex towards ID2 requires the CDH1 co-activator^24^, we first asked whether destruction of FLAG-ID2 by CDH1 was impaired by the ID2-S5D phospho-mimetic mutation. Expression of CDH1 led to marked decrease of wild-type ID2 and ID2-S5A but ID2-S5D and ID2-DBM were resistant to CDH1-mediated destabilization (Fig. 3a). To determine the relevance of each phosphorylation event at the N-terminus for destruction of ID2, we monitored the rate of degradation of ID2 wild type and ID2 mutants S5A, S5D, S14D and T27E. In transiently transfected HeLa cells, the half-life of ID2-S5D was extended ~5-fold compared to ID2 wild type (Fig. 3b, c). Conversely, the S5A phospho-mutant was degraded more efficiently than the wild type protein. The rates of degradation of the S14D and T27E phospho-mimic mutants were comparable to wild type ID2. The inefficient degradation of ID2-S5D and the increased instability of ID2-S5A were independently confirmed by determining their half-life in a different cell line (U251 glioma, Supplementary Fig. 4a, b). These findings indicate that the inability to recruit core APC by ID2-S5D phospho-mimetic mutant leads to inefficient degradation.

As the primary function of the APC^CDH1^ ubiquitin ligase is to control mitotic exit and entry into the G1 phase of cell cycle, we sought to identify the S5 kinase of ID2. We also set out to determine whether ID2-S5 phosphorylation is regulated during cell cycle progression and whether such regulation is associated with changes in ID2 protein accumulation and the ID2–APC interaction. To uncover the cell cycle-dependent regulation of ID2-S5 phosphorylation, we generated and validated the specificity of antibodies against each of the three phospho-ID2 peptides (S5, S14, T27, Supplementary Fig. 4c)^30^. To monitor cell cycle regulation of ID2-S5 phosphorylation independently of the well-known transcriptional fluctuations of the *ID2* gene^31,32^, we generated stable HeLa cells expressing FLAG-ID2, synchronized them at the G1/S interphase by double thymidine block and released cells into cycling at different times after the block. Phosphorylation of ID2-S5 was low in arrested cells (time “0”) but progressively increased as cells moved into active cycling with maximal levels reached 8 h after release, a time corresponding to transition through mitosis, as evidenced by cell cycle profiles with fluorescence activated cell sorting (FACS) and positivity for phospho-histone H3 (Fig. 3d, Supplementary Fig. 4d). Mimicking the abrupt collapse of mitotic cyclins that marks exit from mitosis and entry into G1 10 h after release from the thymidine block, ID2-S5 phosphorylation and the total cellular levels of ID2 were also markedly down-regulated. To determine whether the changes of ID2-S5 phosphorylation detected as cells transit through mitosis and enter into G1 might impact the ability of ID2 to bind core APC, we immunoprecipitated FLAG–ID2 using limiting volume of anti-FLAG beads, thus saturating and equalizing the amounts of FLAG–ID2 complexes captured at different cell cycle times, and analyzed ID2 phosphorylation of FLAG immunoprecipitates using phospho-ID2-specific antibodies. We selected these experimental conditions to interrogate whether changes of ID2-S5 phosphorylation coincide with specific changes in the association of ID2 with core APC, in a manner that would be independent from the total amount of FLAG-ID2 in the cell. We found that ID2-S5 phosphorylation collapsed in cells re-entering G1 at 10 h whereas phospho-S14-ID2 and phospho-T27-ID2 remained stable at all times. Maximal ID2-S5 phosphorylation was detected in mitotic cells collected 8 h after release from the block and coincided with the lowest levels of the ID2-core APC (APC3 and APC1) complex. The ID2-core APC interaction was efficiently restored as cells exited mitosis and re-entered into the G1 phase of the next cycle with very low levels of ID2 phosphorylated on S5 (Fig. 3e, Supplementary Fig. 4e, f).

We also monitored mitotic exit by the loss of pHH3 and Cyclin B1 in cells arrested in G2/M by the specific CDK1 inhibitor RO3306^33^ and harvested at different times after release. Under such experimental conditions, we again observed increased ID2-S5 phosphorylation in mitotic cells (30 and 60 min after release from the RO3306 arrest) and loss of ID2-S5 phosphorylation associated with decreased ID2 abundance as cells exited mitosis (Supplementary Fig. 5a). These changes were associated with impaired recognition of ID2 by core APC (APC1 and APC3) in mitotic cells. The ID2–core APC complex was rapidly restored as cells transitioned from M into G1 (90 min after release from the RO3306 arrest, Supplementary Fig. 5b).

In previous work, it was proposed that cyclin dependent kinases (CDKs) were responsible for phosphorylation of ID2 on serine 5^34^. To unravel the identity of the cellular CDK that phosphorylates ID2 in vivo, we used the phospho-specific ID2-pS5 antibody for western blot of HeLa cells expressing FLAG-ID2 and treated with the CDK4/6 inhibitor palbociclib or the Cdk1/2 inhibitor dinaciclib. ID2-S5 phosphorylation was efficiently inhibited by dinaciclib in a dose-dependent manner whereas palbociclib was ineffective (Fig. 3f, Supplementary Fig. 5c, d). To ask whether the main mitotic cyclin–CDK complex (Cyclin B–CDK1) was responsible for the observed changes of ID2-S5 phosphorylation and whether this phosphorylation event directly regulates the formation of the complex with core APC, we first performed in vitro kinase assay and found that purified Cyclin B-CDK1 efficiently and specifically phosphorylated S5 of ID2 (Fig. 3g). Next, we interrogated the ability of ID2 to bind core APC following phosphorylation of ID2-S5 by active CDK1 under controlled conditions in vitro. In these experiments, GST-ID2 was first phosphorylated on S5 by CDK1 and then challenged for its ability to capture core APC purified from HeLa cells. In the absence of the CDK1-mediated ID2-S5 phosphorylation step, GST-ID2 efficiently captured both core APC (represented by APC3 and APC1) and CDH1 but not CDC20. As expected, core APC components and CDC20 were co-precipitated by histidine-tagged securin (Supplementary Fig. 5e). When GST-ID2 pull-down of APC was done after in vitro phosphorylation of ID2-S5 by Cyclin B-CDK1, phospho-S5-GST-ID2 failed to capture core APC without losing the ability to interact with CDH1 (Fig. 3h).

Consistent with a model in which S5 phosphorylation impairs efficient recognition of the ID2 substrate by the APC ubiquitin ligase complex, in vitro ubiquitylation assays revealed that, compared with wild type ID2 and the ID2-S5A phospho-mutant, the phospho-mimetic mutant ID2-S5D but not ID2-S14D was a poor ubiquitylation substrate of purified APC^CDH1^. The ID2-DBM mutant that cannot bind CDH1 was also inefficiently ubiquitylated by APC^CDH1^. ID2 was not ubiquitylated by APC^CDC20^, which efficiently ubiquitylated securin, thus confirming that CDH1 is the only APC co-activator that targets ID2 for ubiquitylation (Fig. 3i and Supplementary Fig. 5f). Similar findings emerged from in vivo ID2 ubiquitylation assays in which FLAG-ID2-S5D exhibited markedly lower efficiency of polyubiquitylation compared to wild type FLAG-ID2. The protective effect of ID2-S5 phosphorylation against ubiquitylation was also apparent from the increased ubiquitylated forms of ID2-S5A in these experiments. Conversely, neither the S14A and S14D mutations affected ID2 ubiquitylation by APC^CDH1^ (Fig. 3j).

These findings indicate that the high Cyclin B-CDK1 activity of mitotic cells triggers maximal ID2-S5 phosphorylation, which in turn compromises the interaction with core APC, thus preventing premature degradation of ID2. As cells exit mitosis and enter G1, mitotic cyclins collapse, ID2-S5 is dephosphorylated, the APC–ID2 complex is restored and ID2 is efficiently targeted for ubiquitin-dependent degradation by APC^CDH1^.

### Regulation of ID2-S5 phosphorylation controls ordered exit from mitosis

**Fig. 4:**
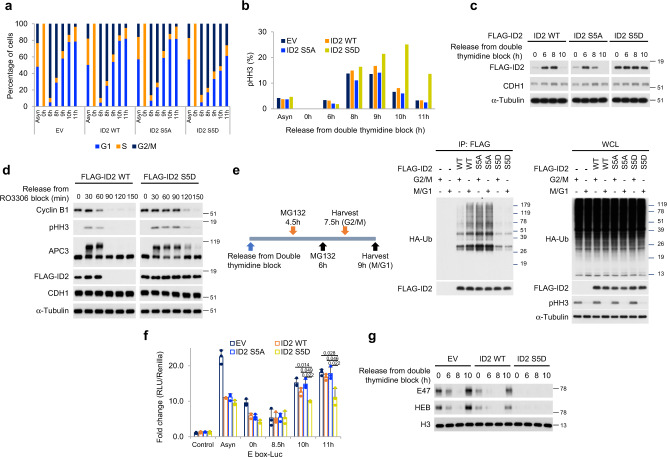
Impaired destabilization of the phospho-mimetic ID2 S5D mutant delays mitotic exit. **a** HeLa cells expressing ID2 WT or S5 phospho-mutants were exposed to double thymidine block and analyzed by flow cytometry. Bar graph shows quantification of cells in G1, S, and G2/M in one representative experiment. Experiments were performed three times with similar results. **b** HeLa cells treated as in **a** were immunostained with pHH3 and analyzed by flow cytometry. Bar graph shows quantification of pHH3-positive cells. **c** Loss of regulation of ID2-S5D during S-G2/M transition. Western blot of HeLa cells expressing FLAG-ID2 WT or S5 phospho-mutants exposed to double thymidine block. **d** Exit from mitosis is delayed by expression of ID2-S5D mutant. Western blot of HeLa cells expressing FLAG-ID2 WT or ID2-S5D treated with RO3306. **e** Left panel, schematics of the cell synchronization protocol for the analysis of ubiquitylation of ID2 WT and phospho-mutants at the G2/M and M/G1 transition. Middle panel, western blot of HeLa cells co-expressing FLAG-ID2 WT or phospho-mutants and HA-ubiquitin immunoprecipitated with FLAG antibody. Right panel, western blot of whole cellular lysates (WCL). Molecular weight markers are indicated in kDa. Experiments were repeated two times with similar results. **f** HeLa cells expressing ID2 wild type or S5 phospho-mutants were transiently transfected with a plasmid expressing the E-box-luciferase cassette. Cells were synchronized by double thymidine block and analyzed for luciferase expression. Bar graph indicates means ± SD of three replicates. *p*-values are from unpaired *t*-test, unequal variance. Experiment was repeated three times with similar results. **g** Loss of binding to chromatin of E47 and HEB in cells overexpressing ID2 S5D. Western blot of chromatin fractions of HeLa cells transfected with ID2 WT or ID2-S5D, synchronized by double thymidine block, and collected at serial times after release. Experiments were repeated two times with similar results. In **d**, APC3 and Cyclin B1 are from the same blots; pHH3, CDH1 are from independent gels; in **e**, HA and FLAG are from different gels; in **g**, E47 and HEB/pHH3 are from different gel. Loading controls are from the same gel as FLAG except in **e** where α-tubulin is from the same gel as pHH3.

As dephosphorylation of ID2-S5 coincides with exit from mitosis and entry into G1, we sought to establish the consequences of preventing ID2-S5 de-phosphorylation in synchronized cells transiting through mitosis. Towards this goal, we generated stable transfectants of HeLa cells expressing wild type FLAG-ID2, the phospho-mutant FLAG-ID2-S5A and the phospho-mimetic mutant FLAG-ID2-S5D. Following synchronization in G1-S by double thymidine, cells were released, harvested at multiple time points and monitored for quantitative cell cycle analysis and phospho-histone H3 by FACS. Approximately 80% of cells transduced with empty vector, FLAG-ID2 and FLAG-ID2-S5A completed transition through mitosis and re-entered G1 10 h after release from the block. However, only 43% of cells expressing FLAG-ID2-S5D exited mitosis and entered into G1 at 10 h (Fig. 4a). Similarly, the quantitative analysis of cells positive to the mitotic marker phospho-histone H3 revealed that 10 h after release from G1-S phase arrest 25% of FLAG-ID2-S5D expressing cells retained positivity for phospho-histone H3 but the positivity of cells transduced with empty vector, FLAG-ID2 and FLAG-ID2-S5A was markedly lower (6–8%, Fig. 4b, Supplementary Fig. 6). The impaired mitotic exit of cells expressing FLAG-ID2-S5D persisted 11 h after G1-S phase arrest (Fig. 4a, b, Supplementary Fig. 6). As expected, FLAG-ID2 wild type and to a larger extent FLAG-ID2-S5A were depleted as cells exited mitosis and re-entered G1 10 h after G1-S phase block. Conversely, FLAG-ID2-S5D levels remained stable throughout the experiment (Fig. 4c). Accordingly, whereas the wild type FLAG-ID2 protein was rapidly eliminated (90 min) in cells released from a G2/M arrest induced by the CDK1 inhibitor RO3306, FLAG-ID2-S5D was stable and delayed the elimination of the mitotic proteins pHH3 and Cyclin B1 (Fig. 4d, Supplementary Fig. 7a–e).

To ask whether the kinetics of depletion of ID2 from cells progressing through mitosis reflected the kinetics of the ubiquitin conjugation reaction upon ID2 and whether the S5D mutation impacted ID2 ubiquitylation, we analyzed FLAG-ID2 ubiquitylation in cells harvested at 7.5 h (G2/M) and 9 h (M/G1) after S phase arrest by double thymidine. Ubiquitylation of wild type FLAG-ID2 was detectable at G2/M and increased as cells exited mitosis whereas FLAG-ID2-S5A was maximally ubiquitylated at G2/M and ubiquitylation persisted in cells re-entering G1. Conversely, the S5D phospho-mimetic mutation prevented most ID2 ubiquitylation at both time points (Fig. 4e). Taken together, the above findings indicate that loss of binding to core APC by the S5D mutation prevents ubiquitylation and destruction of ID2 at the M-G1 interphase and results in inefficient exit from mitosis.

### Id2-S5 phosphorylation coordinates control of mitotic genes with inverse regulation of stemness and cell identity-specific gene expression programs

ID2 is a negative regulator of bHLH transcription factors, which are among the most important determinants of cell identity and differentiation in mammalian cells^25–29^. Mitotic cells exhibit a global arrest of transcription that is relieved at the M-G1 interphase to implement cell type-specific functions and differentiation^17–21^. Thus, we asked whether the phospho-S5-mediated regulation of ID2 stability through controlled interaction with core APC might provide the cross-talk between the intrinsic mitotic regulatory machinery and reactivation of tissue-specific transcriptional programs by bHLH transcription factors. Interestingly, recent work reported that bHLH transcription factors dissociate from mitotic chromosomes^35^. As the ubiquitously expressed E proteins are obligate partners of all bHLH transcription factors and cannot bind DNA in the presence of ID2^27^, we measured E-protein-mediated transcription with an E-box-luciferase reporter in cells transiting through mitosis. By comparing E-box-luciferase activity in cells transduced with empty vector, wild-type ID2, ID2-S5A and ID2-S5D, we also asked whether E-protein-mediated transcription was affected at each time point by ID2-S5 phosphorylation status. In cells transduced with empty vector, wild type ID2 and ID2-S5A and released from a double thymidine block for 8.5 h (mitosis), E-protein-mediated transcription was repressed, suggesting transcriptional inhibition of bHLH target genes in mitosis. As cells exited mitosis and entered G1 (10 and 11 h), bHLH transcriptional activity was restored. However, this effect was blunted in cells expressing ID2-S5D, thus indicating that the stabilized ID2-S5D protein delays G1-specific reactivation of bHLH-mediated transcription (Fig. 4f). These findings were corroborated by the analysis of the association with chromatin by the ubiquitous bHLH E proteins E47 and HEB^28^. In cells transduced with empty vector and wild type ID2, E proteins dissociated from chromatin in cells transiting through mitosis (8 h) but the association of E proteins with chromatin was fully restored in cells re-entering G1 (10 h). Conversely, expression of ID2-S5D prevented the association of E proteins with chromatin as cells exit mitosis (Fig. 4g), thus indicating that re-association of E proteins with chromatin that marks entry into G1 requires dephosphorylation of S5 of ID2.

**Fig. 5:**
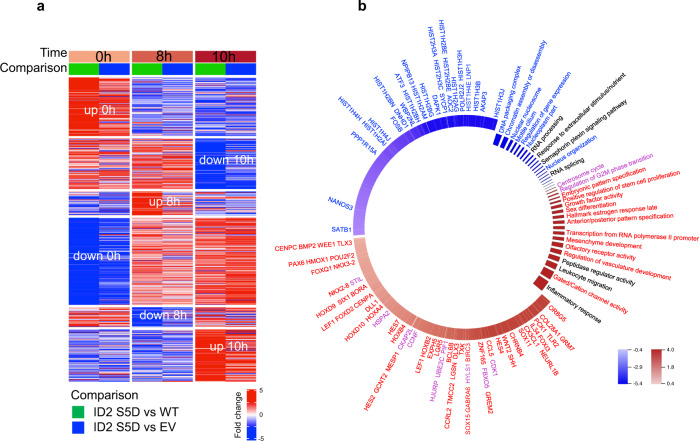
The transcription profile of ID2 S5D expressing cells denotes the inability to establish the G1 phase of the cell cycle. **a** Heatmap representing differentially expressed genes of triplicate biological samples comparing cells overexpressing ID2-S5D with ID2 wild type (green track) or with the empty vector (blue track) synchronized by double thymidine block (0), early M phase (8 h), and early G1 (10 h). **b** Circos plot showing genes and pathways perturbed in cells expressing ID2 S5 phospho-mimetic mutant compared with ID2 wild type expressing cells. Cells were synchronized by double thymidine block, released in normal medium and collected 10 h later (M-G1 transition). Differential gene expression was evaluated using MWW-GST (two-sided). Genes and pathways downregulated and upregulated are in blue and red, respectively. The experiment was performed using three independent biological replicates.

To uncover the global transcriptomic changes triggered by the persistent expression of the phospho-mimic ID2-S5D mutant in cells transiting through mitosis, we analyzed HeLa cells transduced with empty vector, ID2 wild type and ID2-S5D and released into cycle after double thymidine arrest (Fig. 5a, Supplementary Data 1). The comparative analysis of the global transcriptome of cells transduced with empty vector, ID2 wild type and ID2-S5D at mitosis-G1 transition (10 h after the arrest) by RNA-seq revealed that ID2-S5D suppressed the expression of multiple genes implicated in chromatin organization/assembly and positive regulation of gene expression (genes and pathways repressed by ID2-S5D are indicated in blue in Fig. 5b, Supplementary Fig. 8a, Supplementary Data 1, 2). Prominent among the genes down-regulated in cells expressing ID2-S5D are members of the four classes of replication-dependent histone genes, including H2A (4), H2B (7), H3 (6) and H4 (5), corresponding to 34% of the full complement of human histone genes. We also observed reduction of the pioneer/primer transcription factors ATF3^36^ and FOSB^37^, and genes involved in the organization of the nuclear pore complex LNP1^38^ and NPIPB13 (nuclear pore complex interacting protein). These genes and the biological pathways they effect mirror the activities that have to be restored for ordered M-G1 transition and progression of the cell cycle^17,39^. Conversely, the analysis of the genes and biological pathways that remained aberrantly elevated in ID2-S5D cells compared to vector and ID2 wild type expressing cells as they exited mitosis could be grouped into two categories, the first enriched with mitotic functions (genes and pathways indicated in purple in Fig. 5b), the second in stem cell and development-related functions (genes and pathways indicated in red in Fig. 5b). Mitotic genes de-repressed by ID2-S5D included key regulators of mitosis such as CDK1, FBXO5/EMI1, cyclin F and HJURP^39–44^. Interestingly, the *FBXO5/EMI1* gene codes for the EMI1 protein, a potent inhibitor of APC^CDH1^ ^45,46^. Therefore, the persistent elevation of EMI1 in cells expressing ID2-S5D (Fig. 5b, Supplementary Fig. 8b) may directly contribute to delaying APC-mediated destruction of cyclin B1 and CDK inactivation (Fig. 4b, d). The stem cell and development-specific genes that ID2-S5D kept at high level of expression at mitosis-G1 transition included transcription factors (homeobox, T-box, forkhead-related and HES transcription factors) and signaling molecules (SHH, BMP, WNT) with essential roles in preserving the stem/undifferentiated cell state and preventing premature initiation of differentiation programs during early embryogenesis^47–62^. Some of the genes down- and up-regulated by ID2-S5D in cells transiting through mitosis emerged as mitosis-specific transcripts from a computational tool for the analysis of time series of gene expression profiles from cells analyzed at multiple time points after release from G1-S arrest (Supplementary Fig. 8, Supplementary Data 3).

Taken together, these data indicate that dephosphorylation of serine-5 of ID2 in mitotic cells is essential for timely re-entry into G1 marked by resumption of bHLH-mediated transcription and activation of the chromatin-specific functions required for activation of cell type-specific differentiation programs. More importantly, ID2-S5 dephosphorylation is required in late mitosis to orchestrate the suppression of mitotic genes with activation of a coordinated set of transcription factors and signaling molecules implicated in the maintenance of the stem cell state that would otherwise prevent activation of cell type-specific transcriptional programs.

### A general phosphorylation-dependent mechanism of recognition of APC substrates

**Fig. 6:**
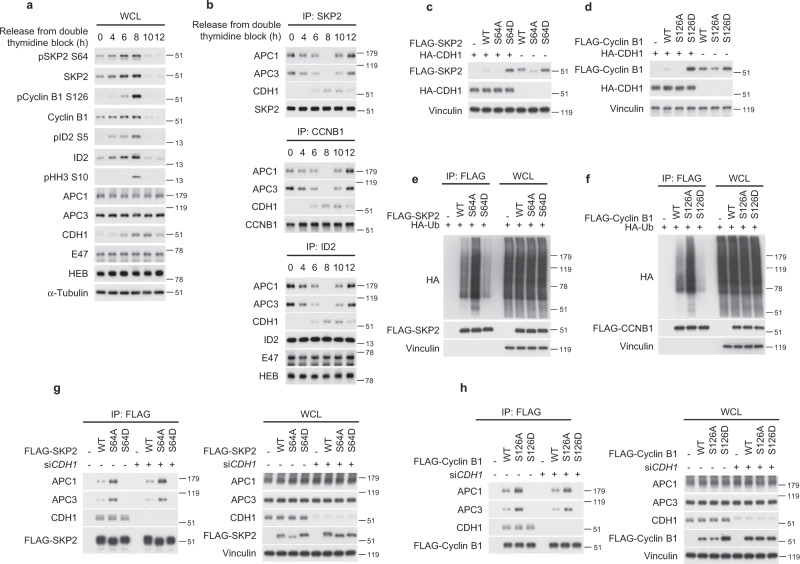
The APC substrates SKP2 and Cyclin B exhibit a phosphorylation-dependent mechanism for the interaction with core APC during cell cycle progression. **a** Western blot of HeLa cells synchronized by double thymidine block using phospho-specific antibodies. **b** Western blot of immunoprecipitates from HeLa cells synchronized by double thymidine block and collected at serial times for immunoprecipitation using SKP2 (upper panel), Cyclin B (middle panel), or ID2 (lower panel) antibodies. E47 and HEB are controls for the immunoprecipitation of endogenous ID2. **c** Loss of CDH1-mediated regulation of SKP2-S64D stability. Western blot of HeLa cells expressing FLAG-SKP2 WT, S64A, or S64D in the presence or absence of CDH1. **d** Loss of CDH1-mediated regulation of Cyclin B-S126D stability. Western blot of HeLa cells expressing FLAG-Cyclin B WT, S126A, or S126D in the presence or absence of CDH1. **e** In vivo ubiquitylation of HeLa cells co-expressing FLAG-SKP2 WT or phospho-mutants and HA-ubiquitin. HA western blot of cellular lyasates immunoprecipitated with FLAG antibody. WCL, whole cellular lysates. **f** In vivo ubiquitylation of HeLa cells co-expressing FLAG-Cyclin B1 WT or phospho-mutants and HA-ubiquitin. HA western blot of cellular lysates immunoprecipitated with FLAG antibody. WCL whole cellular lysates. **g** Loss of interaction between the SKP2-S64D and core APC is independent of CDH1. FLAG-immunoprecipitation-western blot of HeLa cells expressing FLAG-SKP2 WT or phospho-mutants in the presence or the absence of *CDH1* siRNA (left panel). Right panel, whole cellular lysate (WCL). **h** Loss of interaction between the Cyclin B-S126D and core APC is independent of CDH1. FLAG-immunoprecipitation-western blot of HeLa cells expressing FLAG-Cyclin B WT or phospho-mutants in the presence or the absence of *CDH1* siRNA (left panel). Right panel, whole cellular lysate (WCL). Total and phosphorylated proteins are from independent gels; APC1, APC3, CDH1 are from the same gel in each panel; E47, HEB, pHH3, SKP2 and Cyclin B1 are from independent gels; HA and FLAG/Vinculin in panel **e** are from different gels; Loading controls are from the same gel as FLAG or HEB in panel **a**. Molecular weight markers are indicated in kDa. Experiments were repeated two times with similar results.

The finding that optimal destruction of ID2 by APC^CDH1^ requires a dual substrate recognition mechanism, whereby a key role is played by the regulated interaction between core APC subunits and the CDK-dependent ID2-S5 phosphosite, raises the possibility that such mechanism of substrate recruitment by the APC might be more general and involve other substrates. Similar to ID2, the APC^CDH1^ substrates SKP2 and Cyclin B1 have classical D boxes for CDH1 recognition and also harbor established CDK1/2 phosphosites (S64 of SKP2 and S126 of Cyclin B1)^63–65^. We therefore asked whether SKP2 and Cyclin B1 interact with core APC in a cell-cycle regulated manner and whether such interactions are associated with changes of SKP2-S64 and Cyclin B1-S126 phosphorylation. The time-dependent analysis of HeLa cells released from a double thymidine block revealed that mitotic cells marked by positivity for pHH3 (8 h after the block) exhibited the highest levels of SKP2-S64 and Cyclin B1-S126 phosphorylation, which abruptly collapsed together with the total cellular levels of SKP2 and Cyclin B1 as cells exited mitosis. This profile mirrored the changes of ID2-pS5 and ID2 protein accumulation (Fig. 6a). Resembling the dynamic changes of the ID2–core APC complex, the SKP2–core APC and Cyclin B1–core APC complexes were disassembled in mitotic cells (8 h after the block) when the phosphorylation of SKP2-S64 and Cyclin B1-S126 was maximal (Fig. 6b). These findings are consistent with the hypothesis that, as for ID2, CDK1-mediated phosphorylation of SKP2 and Cyclin B1 may prevent interaction with core APC, thus impairing APC^CDH1^-mediated ubiquitylation and degradation of these substrates. To experimentally interrogate this scenario, we generated phospho-deficient (SKP2-S64A, Cyclin B1-S126A) and phospho-mimetic (SKP2-S64D, Cyclin B1-S126D) mutants and tested their ability to function as degradation substrates of CDH1. The phospho-deficient mutants (SKP2-S64A, Cyclin B1-S126A) exhibited lower basal level of expression than the wild type proteins and were efficiently eliminated by CDH1. Conversely, the SKP2-S64D and Cyclin B1-S126D phospho-mimetic mutants were expressed at higher basal levels than wild-type SKP2 and Cyclin B1 and were resistant to CDH1-mediated destabilization (Fig. 6c, d). Accordingly, SKP2-S64D and Cyclin B1-S126D mutants were inefficiently ubiquitylated in vivo whereas the corresponding phospho-mutants (SKP2-S64A, Cyclin B1-S126A) exhibited higher levels of ubiquitylation (Fig. 6e, f). Next, we asked whether phosphorylation of S64 of SKP2 and S126 of Cyclin B1 was implicated in regulation of binding to core APC and/or CDH1. Compared with the respective wild type proteins, the phospho-mimetic mutants (SKP2-S64D, Cyclin B1-S126D) did not bind to core APC but retained the interaction with CDH1. Conversely, the phospho-mutant proteins SKP2-S64A and Cyclin B1-S126A showed increased affinity for core APC, again without changes in the binding to CDH1. The independent modules for core APC versus CDH1 binding by SKP2 and Cyclin B1 was confirmed by the absence of functional consequences upon the SKP2–core APC and Cyclin B1–core APC complexes by silencing of CDH1, regardless of whether the experiments were done using wild type or mutant protein substrates (Fig. 6g, h). Each of the three proteins, ID2, SKP2 and Cyclin B1, contain the CDK-phosphorylated serine (S5 in ID2, S64 in SKP2 and S126 in cyclin B) as an APC interacting segment. No overall local sequence similarity is shared by these three unrelated proteins within four residues of the phosphorylated serine, but the local 3D structures of the seven amino acid peptides centered on the relevant serine residues is similar, and all three are included in disordered/flexible regions of ID2, SKP2 and Cyclin B1, which have resisted crystallographic resolution (Supplementary Fig. 8c). We conclude that APC^CDH1^ regulates ubiquitylation and degradation of ID2, SKP2 and Cyclin B1 through a common mechanism that involves phosphorylation-regulated recognition of these substrates by core APC.

## Discussion

The regulation of destruction of substrates by APC depends on the CDC20 and CDH1 co-activators as degron recognition factors (D-box)^66^. This paradigm also applies to the control of ubiquitin-mediated degradation of ID2 by APC^CDH1^ ^24^. However, our previous work indicated that ID2, similar to the APC substrates NEK2A^67,68^, and cyclin A2–CKS complex^69,70^ binds core subunits of APC independently of the canonical D-box^24^. Here we report mechanism, regulation and significance of the ID2–core APC interaction. We showed that (i) the N-terminal region of ID2 is sufficient and necessary for recognition of core APC; (ii) the N-terminal domain of ID2 interacts with core APC subunits but not CDH1; (iii) binding between the N-terminal domain of ID2 and core APC is negatively regulated by Cyclin B-CDK1-mediated phosphorylation of S5 of ID2. As similar degron-independent and phosphorylation-sensitive recognition of core APC is also shared by the APC substrates Cyclin B1 and SKP2, our work uncovered a more general mechanism of recognition of substrates by core APC and CDH1, respectively, that cooperatively provide optimal orientation of the substrates for the ubiquitylation reaction (Supplementary Fig. 9).

The three-dimensional structural compatibility of the independently derived 3D structures of APC^CDH1^ and ID2 suggest that the segment of the ID2 N-terminus centered on S5 can associate with APC3 while simultaneously the C-terminus D-box associates with the canonical binding site on CDH1, despite their distant 3D locations in the APC complex. Thus, the D-box is necessary but not sufficient for ID2 to recruit the APC holoenzyme and the N-terminal domain including S5 confers a stringent control of interaction and consequent degradation during mitosis-G1 transition. Similar to ID2, the phosphorylation-regulated binding of SKP2 and Cyclin B to APC suggests that at least some APC substrates evolved combinatorial interaction motifs with S5 of ID2, S64 of SKP2, and S126 of Cyclin B specifying an additional level of regulation of the binding (phosphorylation on/off) during mitotic progression and exit. Interestingly, it was proposed that regions of Cyclin B1 different from the D-box mediate interaction with the APC and regulate timely destruction in late mitosis to allow cytokinesis. Although each of the three proteins, ID2, SKP2 and Cyclin B, contain the CDK-phosphorylated serine as an APC interacting segment, there is no overall local sequence similarity surrounding the phosphorylated serine. However, preservation of local structural similarity only without surrounding sequence similarity is typical of phosphosite-regulated binding motifs, especially for ubiquitin ligases (e.g. DSG motif for unrelated substrates of ß-TrCP)^71,72^. A corollary for “floating” motifs like these is that they can occur anywhere in the target protein and in any order relative to other motifs. This appears to be the case with ID2, SKP2 and Cyclin B1 since there is no conserved order of occurrence of the SP motif and the D-box in the three proteins, with the D-box in Cyclin B occurring before the SP motif. Thus, we conclude that the APC interacting SP motif is a typical motif of this kind. Our docked complex of ID2 with APC3 supports this view, as it suggests a tight backbone specific for proline to place the serine in a tight pocket that cannot accommodate phosphorylation and 284 Å^2^ of contact area between the arginine and APC3 (Fig. 2a, d).

The interaction between core APC and ID2 is impaired in mitotic cells when high Cyclin B-CDK1 activity causes maximal phosphorylation of ID2-S5 and minimal degradation of ID2 by APC^CDH1^. Phosphorylation of ID2-S5 collapses at the M-G1 transition thus inducing the recognition of unphosphorylated ID2 by core APC and licensing APC^CDH1^-mediated destruction of ID2. Accordingly, the S5 phospho-mimetic mutant of ID2 (ID2-S5D) was resistant to APC^CDH1^-mediated destruction and delayed entry into G1. Moreover, in the presence of ID2-S5D, the cell cycle dynamics of the binding of bHLH transcription factors (E proteins) to chromatin was perturbed and their transcriptional activation blunted. At the end of mitosis, degradation of de-phosphorylated ID2-S5 provides the mechanism for rapid reactivation of bHLH transcription to reinstate cell identity functions. Thus, as normal cells transit through mitosis, CDK1 activity constitutes a bi-stable switch mechanism for ID2 protein that is operated through the ID2 N-terminus module with ID2 “off” at exit from mitosis. Whereas our findings uncovered a role for ID2-S5 phosphorylation in the control of exit from mitosis, some experiments indicated that the ID2-S5D phospho-mimic mutant has excessive inhibitory activity against E protein binding to DNA and transcriptional activation at the G1/S phase of the cell cycle (see for example, Figs. 4g and 5a). As ID2-S5 can also be phosphorylated by the G1/S CDK2 kinase^34^, our results do not exclude that other biochemical effects, different from the negative regulation of the interaction with core APC, may account for the unrestrained activity of ID2-S5D at the G1/S phase of the cell cycle.

The analysis of the transcriptome of cells transiting through mitosis in the presence of a constitutively phosphorylated ID2 protein (ID2-S5D) highlighted a more complex scenario for the function of ID2 in cells that exit mitosis and shed light on an unexpected new layer of regulation of transcriptional programs during mitosis. Extensive research supports the crucial role of APC^CDH1^ to induce differentiation programs in synchronization with entry and maintenance of the G1 state^23^. Several groups including ours have proposed a positive role of APC^CDH1^ in enforcing stable differentiation programs in multiple cell types through ubiquitin-mediated degradation of key inhibitors of differentiation such as ID2, SnoN, and SKP2^24,73,74^. In this study, we found that the coordination of mitosis and differentiation requires controlled degradation of ID2 by APC^CDH1^. Besides implementing the irreversible G1 state associated with terminal differentiation of certain cell types (e.g., neurons), a similar, albeit transient, APC^CDH1^-mediated degradation of ID2 is essential for timely exit from mitosis and re-entry into G1 of cycling cells. This process is initiated by dephosphorylation of S5 of ID2, an event that promotes the optimal recognition of the ID2 substrate by the core complex of APC. ID2-S5 dephosphorylation triggers the efficient degradation of unphosphorylated ID2 by APC^CDH1^ and the coordinated downregulation of mitotic genes, and transcription factors and signaling molecules (HOX, T-box and HES transcription factors, SHH and WNT signaling components) that act as master regulators of stem cells/cell fate by preventing premature/aberrant differentiation during embryonic development. Conversely, sustained ID2 activity by expression of ID2-S5D in cells exiting mitosis preserved the expression of mitotic genes and stem cell master regulators while suppressing the activation of cell-specific transcriptional programs. The transcriptomic analysis of cells expressing ID2-S5D uncovered a number of de-repressed genes that, together, may have a negative impact on APC activity resulting in impaired exit from mitosis. However, the persistent elevation in mitosis of the APC^CDH1^ inhibitor EMI1^45,46^ by ID2-S5D is likely to play a key role for the delay of APC-mediated destruction of cyclin B1 and CDK inactivation.

The transient “mitotic stem cell state” elicited by CDK1-mediated protection of ID2-phospho-S5 from degradation by APC^CDH1^ suggests a similarity between reprogrammed transcription during mitosis of somatic cells and transcriptional activity of highly cycling embryonic stem cells. It also helps explaining the distinct susceptibility of mitotic cells to reprogramming with stable loss of differentiation^75–77^. In conclusion, our findings provide evidence for the regulation of mitosis to G1 transition by the APC^CDH1^-ID2 S5 phosphorylation pathway.

## Methods

### Plasmids, cloning, and lentivirus production

FLAG-tagged ID plasmids (wild type and mutants) have been described previously^30^. V5-tagged ID2, HA-tagged Ubiquitin, HA-tagged CDH1 and CDC20 were generated by PCR and cloned into pcDNA3 plasmid. FLAG, V5 or HA tags were added at the N- or C-terminus as indicated. cDNA for APC3, SKP2, and Cyclin B1 expression were obtained from Addgene and sub-cloned into pcDNA3 or pLVX vector. All mutants in this manuscript were generated by site-directed mutagenesis using the QuickChange II Site-Directed Mutagenesis kit (Agilent, # 200524) and resulting plasmids were verified by Sanger sequencing. Lentiviral particles were obtained by co-transfection of lentiviral vectors with pCMV-ΔR8.1 and pMD2.G plasmids into HEK293T cells as previously described^30^.

### Cell culture

HeLa (ATCC, CCL-2), HEK293T (ATCC, CRL-11268), and U-2 OS (HTB-96) cell lines were acquired through American Type Culture Collection. U-251 MG were obtained from Millipore-Sigma (# 09063001). Cell lines were cultured in DMEM supplemented with 10% fetal bovine serum (FBS, Sigma). Cells were routinely tested for mycoplasma contamination using Mycoplasma Plus PCR Primer Set (Agilent, Santa Clara, CA # 302008) and were found to be negative. Cells were transfected with Lipofectamine 2000 (Invitrogen, # 11668019) or calcium phosphate. Cells were transduced using lentiviral particles in medium containing 4 μg/ml of polybrene (Sigma). siRNA/CDH1 was purchased from Dharmacon (siGenome Human *FZR1* siRNA SMARTPool, L-015377-00-0010) and transfected using Lipofectamine 2000 (Invitrogen, # 11668019).

### Cell cycle synchronization and flow cytometry analysis

HeLa cells were synchronized by double thymidine block. Briefly, cells were treated with 2 mM thymidine for 18 h. Cells were washed with phosphate buffered saline (PBS) and allowed to grow in regular growth medium for 9 h. Cells were then exposed again to 2 mM thymidine for 15 h. Cells synchronized at the G1-S phase of the cell cycle were released after extensive washes with PBS in fresh medium and collected at serial time points as indicated in figures. For synchronization in late G2, HeLa cells were treated by 10 μM RO3306 for 20 h. After extensive washes with PBS, cells were released in fresh medium and collected at the time points indicated in figures.

Cells were harvested by trypsinization, washed in PBS and fixed in cold 70% ethanol overnight. Fixed cells were washed twice in PBS, treated with ribonuclease A 100 μg/ml in PBS plus 0.1% Triton X-100 and stained with propidium iodide at concentration of 50 mg/ml for at least 2 h before the analysis using FACSCalibur instrument (BD) and collecting the area, height, and width parameters for the DNA channel in addition to forward scatter and sideward scatter. For phospho-histone-H3 staining, cells harvested and fixed in cold 70% ethanol were permeabilized in PBS containing 0.25% triton X-100 for 15 min, washed in PBS containing 1% bovine serum albumin and immunostained using Alexa Fluor 647-conjugated histone-H3 phospho-Ser-10 antibody (1:50; Cell Signaling Technology # 3458) in PBS containing 1% bovine serum albumin for 1 h at room temperature. After three washes in PBS, cells were stained with propidium iodide in PBS containing 100 μg/ml ribonuclease A for 30 min and analyzed by flow cytometry using LSR II Flow Cytometer (BD Biosciences, San Jose, CA). Debris and cell aggregates were excluded using the appropriate FSC versus SSC gates. BD FACSDiva Software v.7.0.1 was used for acquisition; for analysis we used FCS Express 6 Flow v.7.12.007 or FlowJo v.10.7.1. In every experiment at least 10,000 cells for each sample were evaluated.

### Immunoprecipitation, western blot, and in vitro GST/histidine pull-down assay

Cells were lysed in NP40 lysis buffer [50 mM Tris–HCl, pH 7.5, 150 mM NaCl, 1 mM EDTA, 1% NP40, 1.5 mM Na_3_VO4, 50 mM sodium fluoride, 10 mM sodium pyrophosphate, 10 mM β-glycerolphosphate and EDTA free protease inhibitor cocktail (Roche)] or RIPA buffer [50 mM Tris–HCl, pH 7.5, 150 mM NaCl, 1 mM EDTA, 1% NP40, 0.5% sodium dexoycholate, 0.1% sodium dodecyl sulfate, 1.5 mM Na_3_VO_4_, 50 mM sodium fluoride, 10 mM sodium pyrophosphate, 10 mM β-glycerolphosphate, and EDTA free protease inhibitor cocktail (Roche)]. Lysates were cleared by centrifuge at 20,000×*g* for 15 min at 4 °C. For immunoprecipitation, cell lysates were incubated with primary antibody and protein G/A beads (Santa Cruz, # sc-2003), FLAG-M2 affinity beads (Sigma, # F2426), or HA affinity matrix (Millipore-Sigma, # 11815016001) at 4 °C overnight. Beads were washed with lysis buffer for four times and eluted in 2× SDS sample buffer or FLAG peptide (Sigma, # F4799). Protein samples were separated by SDS–PAGE and transferred to polyvinyl difluoride (PVDF) or nitrocellulose (NC) membrane. Membranes were blocked in TBS with 5% non-fat milk and 0.1% Tween20, and probed with primary antibodies. Antibodies and working concentrations are: ID2 1:500 (C-20, # sc-489), E2A/E47 1:1000 (N-649, # sc-763) and CDH1/Fzr 1:250 (DCS-266, # sc-56312) obtained from Santa Cruz Biotechnology; HA 1:1000 (C29F4, # 3724 or # 2367), phospho-SKP2 1:1000 (Ser64) (# 14865), SKP2 1:1000 (D3G5, # 2652), CCNB1 1:1000 (D5C10, # 12231), APC1 1:1000 (D1E9D, # 13329), APC3/CDC27 1:1000 (D3I1V, # 12530), TCF12/HEB 1:1000 (D2C10, # 11825), securin1:1000 (D2B6O # 13455) obtained from Cell Signaling Technology; β-actin 1:8000 (# A5441), α-tubulin 1:8000 (# T5168), vinculin 1:5000 (# V9131), and FLAG M2 1:500 (# F1804) obtained from Sigma; HA 1:1000 (3F10, # 12158167001) obtained from Roche; phospho-Cyclin B1 (S126) 1:1000 (# ab55184) obtained from Abcam. Secondary antibodies anti-mouse (# 32460), anti-rabbit (# 32430), and anti-rat (# 31470) horseradish-peroxidase-conjugated were purchased from ThermoFisher Scientific and ECL or ECL PLUS reagent (GE Healthcare Amersham) was used for detection. For in vitro binding assay, Glutathione-S-Transferase (GST)-tagged ID2 (wild type and mutants), or GST-ID1 proteins were expressed in *E. coli* BL21(DE3) (Thermo Scientific # EC0114) and immobilized on glutathione sepharose 4B affinity matrix (Sigma, # GE17075601). 6X-Histidine-tagged securin protein was obtained from Abcam (ab87664) and immobilized on Ni-NTA-Superflow agarose (Pierce-Thermo Scientific 25214). Immobilized proteins were incubated with cell lysate prepared in NP40 lysis buffer for 2 h, and after extensive washing steps, protein complexes were eluted using Laemmli sodium dodecyl sulfate (SDS) containing sample buffer and analyzed by western blot.

### In vitro kinase assays

Recombinant active Cdk1 kinase was purchased from ThermoFisher (# PV3980). Five hundred nanograms of GST-ID proteins were incubated with 10–20 ng of the active kinase. The reaction mixture included 10 μCi of [γ-^32^P]ATP (PerkinElmer Life Sciences, # BLU002Z250UC) in 50 μl of kinase buffer (25 mM Tris–HCl, pH 7.5, 5 mM β-glycerophosphate, 2 mM dithiothreitol (DTT), 0.1 mM Na_3_VO_4_, 10 mM MgCl_2_, and 0.2 mM ATP). Reactions were incubated at 30 °C for 30 min. Reactions were terminated by addition of Laemmli SDS sample buffer and boiling on 95 °C for 5 min. Proteins were separated on SDS–PAGE gel and phosphorylation of proteins was visualized by autoradiography or phospho-specific antibodies. Coomassie staining of GST-fusion proteins was performed to document the amounts of substrates included in the kinase reaction.

### Evaluation of protein half-life

HeLa cells expressing FLAG-tagged ID2 were treated by 50 μg/ml of cycloheximide (CHX) for the indicated times and analyzed by western blot. ID2 half-life was quantified by densitometry using ImageJ processing software (NIH). Densitometry values were analyzed by Prism 6.0 using the linear regression function.

### Ubiquitylation assays

#### In vivo *ubiquitylation*

Cells were transfected with FLAG-tagged substrates (Cyclin B1, SKP2, and ID2) and pcDNA3-HA-Ubiquitin. 36 h after transfection, cells were treated with 5 μM MG132 (EMD Millipore, # 474790) for 10 h or the indicated time. After two washes with ice-cold PBS, cells were collected and lysates prepared in 100 μl of buffer 50 mM Tris–HCl pH 8.0, 150 mM NaCl (TBS), and 2% SDS and boiled at 100 °C for 10 min. Lysates were diluted with 900 μl of tris buffered saline containing 1% NP40. Immunoprecipitation was performed using 1 mg of cellular lysates. Proteins were immunoprecipitated using anti-FLAG affinity matrix (Sigma, # F2426) and analyzed by western blot using the indicated antibodies.

#### In vitro *ubiquitylation*

HeLa cells expressing FLAG-APC3/CDC27 were synchronized by the use of 100 ng/ml nocodazole overnight. Cells in prometaphase were harvested by mitotic shake off and wash twice in ice cold PBS. Cells were lysed in NP40 buffer (50 mM Tris, pH 7.5, 250 mM NaCl, 1% NP40, 0.1% Triton X-100, 1 mM EDTA, 1 mM DTT, and phosphatase and protease inhibitors). Four mg of cell lysate were immunoprecipitated with anti-FLAG affinity matrix at 4 °C for 2 h 30 min. Anti-FLAG affinity matrix was washed four times with NP40 buffer and twice with modified QA buffer (10 mM Tris–Cl, pH 7.5, 100 mM KCl, 1 mM MgCl_2_, 0.1 mM CaCl_2_, 0.1% NP40, and phosphatase and protease inhibitors). Purified APC complex was activated using in vitro translated CDH1 or CDC20. Activated APC complex was washed four times in modified QA buffer and eluted in 4 μl of FLAG peptide dissolved in TBS on ice for 1 h. ID2-V5 substrates were synthesized by in vitro translation. Recombinant 6X-histidine-tagged securin was obtained from Abcam (ab87664). In vitro ubiquitylation assay was performed in 15 μl of reaction mixture containing 0.3 μM UBE1 (Boston Biochem, # E-304), 1.2 μM UBE2C (Enzo, # BML-UW0960-0100), 0.6 μM UBE2D1 (Boston Biochem, # E2-800), 0.6 μM UBE2D3 ((Boston Biochem, # E2-802), 1 μM Ubiquitin aldehyde (Boston Biochem, # U-201), 166 μM Ubiquitin (Boston Biochem, # U-100H) diluted in 1× Ubiquitin reconstitution buffer (Boston Biochem, # B-90), APC complex, substrate (ID2-V5), 1× energy regeneration solution (Boston Biochem, # B-10), and E3 ligase reaction buffer (Boston Biochem, # B-71). In vitro ubiquitylation was performed at 30 °C for 30 min. Reactions were terminated by the addition of SDS sample buffer and analyzed by western blot using the indicated antibodies.

### Chromatin fractionation

Chromatin fractionation was performed according to published method^78^. Briefly, 5 × 10^6^ cells were washed in ice-cold PBS twice and resuspend in 200 μl buffer A [10 mM Tris-HCl buffer, pH 7.5, 10 mM KCl, 1.5 mM MgCl_2_, 0.34 M sucrose, 10% glycerol, 1 mM DTT, 1.5 mM sodium orthovanadate, 50 mM sodium fluoride, 10 mM sodium pyrophosphate, 10 mM β-glycerolphosphate, and EDTA free protease inhibitor cocktail (Roche)] containing 1 mM DTT, protease inhibitors and 0.1% Triton X-100 for 5 min on ice. Nuclei were collected by centrifugation at 1300×*g* for 4 min at 4 °C. Nuclei were washed in 200 μl buffer A and centrifuged at 1300×*g* for 4 min. Nuclei were then lysed in 200 μl buffer B [3 mM EDTA, 0.2 mM EGTA, 1 mM DTT and 1.5 mM sodium orthovanadate, 50 mM sodium fluoride, 10 mM sodium pyrophosphate, 10 mM β-glycerolphosphate and EDTA free protease inhibitor cocktail] for 30 min on ice. The insoluble chromatin pellet was centrifuged at 1700×*g* for 4 min at 4 °C, washed once in buffer B, centrifuged again under the same conditions and dissolved in 4% SDS containing buffer followed by boiling. Immunoprecipitation was performed as indicated above and immunoprecipitates analyzed by western blot.

### Modeling of APC complex–ID2 interaction

The peptide corresponding to amino acids 2–8 of ID2 (ID2 amino acids 2-8) was docked to both the 3.3 Å crystallographic structure of the APC3 homodimer (PDB 4rg6)^79^ and the 3.6 Å cryo-EM structure of holo APC (PDB 4ui9)^80^, as previously described^81^. Briefly, grid potentials representing van der Waals, hydrogen bonding, Coulombic, and solvation electrostatics were generated from the APC3 homodimer coordinates and the coordinates of all the subunits surrounding (APC1, APC3, APC6, APC7, APC10, APC13, APC16) and including CDH1, respectively; a conformational search of a full atom model of ID2 peptide amino acids 2-8 was performed within the space of these grid potentials. As both top docked conformations of the ID2 peptide within APC3 alone and within the wider CDH1-centered APC region were similar, a parallel docking with ID2 peptide amino acids 2-8 phosphorylated at S5 (ID2-pS5-2-8) and also with S5 mutated to D (ID2-D5-2-8) was performed for the APC3 grid alone. The conformation docked to the APC3 homodimer alone was placed within the larger cryo-EM structure of the whole APC^CDH1^ complex (PDB: 4ui9)^80^ to evaluate its position relative to the D-box-binding site on CDH1. A full length model of ID2 with its N-terminal 2–8 residues in the APC3 homodimer-docked position in situ within the whole APC^CDH1^ complex and its C-terminal D-box superimposed on the CDH1-bound D-box (PDB 4bh6)^82^. Superimposition of the 4-helix bundle homodimer of ID2 (PDB 4aya)^83^ showed that this 4-helix bundle could fit without clashing into the cavity between CDH1, APC7, APC10, and APC16 in the intact APC^CDH1^ complex. Energy minimization of the flexible ID2 linker (amino acids 9–29) between this location of the 4-helix bundle and the APC3-docked N-terminus using van der Waals, torsional, and electrostatic terms showed that the linker could adopt an un-clashed conformation connecting the C-terminus of the APC3-docked, ID2 amino acids 2-8, and the N-terminus of the first helix in the ID2 4-helix bundle. Similarly, the linker connecting the ID2 4-helix bundle to the C-terminal D-box was energetically compatible. ID2-pS5-2-8 and ID2-D5-2-8 however, docked to a different location on APC3, exhibiting strong electrostatic interaction between the phosphate/aspartate and a surface of APC3. The new location was too close to the ID2 4-helix bundle to accommodate any folded conformation of the ID2 linker consisting of amino acids 9–29. All molecular modeling was performed with ICM-Pro (Molsoft LLC, La Jolla, CA, USA).

### RNA sequencing and analysis

HeLa were synchronized by double thymidine block. Total RNA was purified with TRIZOL (ThermoFisher, #155960026). Total RNA was poly(A)-selected and sequencing libraries were constructed using the TruSeq stranded mRNA Library Prep (llumina, # 20020594) and Seq RNA CD Index Plate (Illumina, 20019792). Library were sequenced on a NovaSeq 6000 System (Illumina). We performed a pseudoalignment to a kallisto index created from transcriptomes (GRCh38) using kallisto^84^, and computed the estimated counts for each gene using tximport R package^85^. The final matrix was composed of 27 samples (3 cell conditions: ID2 S5D, ID2 WT, EV; 3 time points: 0, 8, 10 h; 3 replicates each). Downstream analysis of gene expression was performed in the R statistical environment. To test whether the expression trajectory of a gene over time differs between ID2-S5D and ID2 wild type samples, we used the ImpulseDE2 R package, an algorithm for differential expression of longitudinal sequencing experiments^86^. The algorithm produces gene-wise expression trajectories over time with a descriptive single-pulse (impulse) function, and is based on a negative binomial noise model with dispersion trend smoothing by DESeq2 and uses the impulse model to constrain the mean expression trajectory of each gene. We defined a gene up-/down-regulated between ID2-S5D versus ID2 wild type if ImpulseDE2 *q* < 0.05, $\left|{{{{{{\rm{log }}}}}}}_{2}{{{\rm {FC}}}}_{{10}\, {\rm {h}}}\right| \; > \; 0.58$, two-sided *t*-test, unequal variance *p*_10h_ < 0.05, that resulted in 22 up-regulated and 12 down-regulated genes in cells expressing ID-S5D. Gene ontology enrichment analysis using the full ranked list of genes defined as the ordered set of genes on the basis of the ${{{{{{\rm{log }}}}}}}_{2}{{{\rm {FC}}}}_{{10}\,{\rm {h}}}$ between ID2 S5D versus ID2 WT expressing samples collected 10 h after double thymidine block was obtained using MWW-GST from the yaGST R package (two-sided MWW-GST, *p* < 0.01, $\left|{{{{{\rm{logit}}}}}}\left({{\rm {NES}}}\right)\right| \; > \; 0.3$)^87^. Genes reported in the circos plot in Fig. 5b are up-/down-regulated in ID2-S5D compared with ID2 WT (${\left|{{{{{{\rm{log }}}}}}}_{2}{{{\rm {FC}}}}_{{10}\,{\rm {h}}}\right| \; > \; 0.4}$).

### Statistics

Results in graphs are expressed as means ± SD or SEM as indicated in figure legends, for the indicated number of observations. Statistical significance was determined by the Student’s *t*-test (two-tailed, unequal variance) using GraphPad Prism 6.0 software package (GraphPad Inc.). *p*-value < 0.05 is considered significant and is indicated in figure legends.

### Reporting summary

Further information on research design is available in the Nature Research Reporting Summary linked to this article.

## Supplementary information

Supplementary Information

Description of Additional Supplementary Files

Supplementary Data 1–3

Reporting Summary

## Data Availability

The data that support this study are available from the corresponding authors upon reasonable request. The RNA-seq data generated in this study are available from the Gene Expression Omnibus (GEO) with the accession no. GSE181639. Source data are provided with this paper.

## Source data

Source Data

## References

[1] Gerard C, Goldbeter A (2012). From quiescence to proliferation: Cdk oscillations drive the mammalian cell cycle. Front. Physiol..

[2] Murray AW (2004). Recycling the cell cycle: cyclins revisited. Cell.

[3] Watson ER, Brown NG, Peters JM, Stark H, Schulman BA (2019). Posing the APC/C E3 ubiquitin ligase to orchestrate cell division. Trends Cell Biol..

[4] Gieffers C, Dube P, Harris JR, Stark H, Peters JM (2001). Three-dimensional structure of the anaphase-promoting complex. Mol. Cell.

[5] Zachariae W, Shin TH, Galova M, Obermaier B, Nasmyth K (1996). Identification of subunits of the anaphase-promoting complex of *Saccharomyces cerevisiae*. Science.

[6] Tugendreich S, Tomkiel J, Earnshaw W, Hieter P (1995). CDC27Hs colocalizes with CDC16Hs to the centrosome and mitotic spindle and is essential for the metaphase to anaphase transition. Cell.

[7] Sudakin V (1995). The cyclosome, a large complex containing cyclin-selective ubiquitin ligase activity, targets cyclins for destruction at the end of mitosis. Mol. Biol. Cell.

[8] King RW (1995). A 20S complex containing CDC27 and CDC16 catalyzes the mitosis-specific conjugation of ubiquitin to cyclin B. Cell.

[9] Chang LF, Zhang Z, Yang J, McLaughlin SH, Barford D (2014). Molecular architecture and mechanism of the anaphase-promoting complex. Nature.

[10] Wei W (2004). Degradation of the SCF component Skp2 in cell-cycle phase G1 by the anaphase-promoting complex. Nature.

[11] Bashir T, Dorrello NV, Amador V, Guardavaccaro D, Pagano M (2004). Control of the SCF(Skp2-Cks1) ubiquitin ligase by the APC/C(Cdh1) ubiquitin ligase. Nature.

[12] Sigrist SJ, Lehner CF (1997). Drosophila fizzy-related down-regulates mitotic cyclins and is required for cell proliferation arrest and entry into endocycles. Cell.

[13] Fang G, Yu H, Kirschner MW (1998). The checkpoint protein MAD2 and the mitotic regulator CDC20 form a ternary complex with the anaphase-promoting complex to control anaphase initiation. Genes Dev..

[14] Kramer ER, Gieffers C, Holzl G, Hengstschlager M, Peters JM (1998). Activation of the human anaphase-promoting complex by proteins of the CDC20/Fizzy family. Curr. Biol..

[15] Fang G, Yu H, Kirschner MW (1998). Direct binding of CDC20 protein family members activates the anaphase-promoting complex in mitosis and G1. Mol. Cell.

[16] Visintin R, Prinz S, Amon A (1997). CDC20 and CDH1: a family of substrate-specific activators of APC-dependent proteolysis. Science.

[17] Palozola KC (2017). Mitotic transcription and waves of gene reactivation during mitotic exit. Science.

[18] Probst AV, Dunleavy E, Almouzni G (2009). Epigenetic inheritance during the cell cycle. Nat. Rev. Mol. Cell Biol..

[19] Michelotti EF, Sanford S, Levens D (1997). Marking of active genes on mitotic chromosomes. Nature.

[20] Gottesfeld JM, Forbes DJ (1997). Mitotic repression of the transcriptional machinery. Trends Biochem. Sci..

[21] Bender MA, Prescott DM (1962). DNA synthesis and mitosis in cultures of human peripheral leukocytes. Exp. Cell Res..

[22] Oh E (2020). Gene expression and cell identity controlled by anaphase-promoting complex. Nature.

[23] Wasch R, Robbins JA, Cross FR (2010). The emerging role of APC/CCdh1 in controlling differentiation, genomic stability and tumor suppression. Oncogene.

[24] Lasorella A (2006). Degradation of Id2 by the anaphase-promoting complex couples cell cycle exit and axonal growth. Nature.

[25] Imayoshi I, Kageyama R (2014). bHLH factors in self-renewal, multipotency, and fate choice of neural progenitor cells. Neuron.

[26] Kageyama R, Shimojo H, Ohtsuka T (2019). Dynamic control of neural stem cells by bHLH factors. Neurosci. Res..

[27] Lasorella A, Benezra R, Iavarone A (2014). The ID proteins: master regulators of cancer stem cells and tumour aggressiveness. Nat. Rev. Cancer.

[28] Massari ME, Murre C (2000). Helix-loop-helix proteins: regulators of transcription in eucaryotic organisms. Mol. Cell Biol..

[29] Murre C (2019). Helix-loop-helix proteins and the advent of cellular diversity: 30 years of discovery. Genes Dev..

[30] Lee SB (2016). An ID2-dependent mechanism for VHL inactivation in cancer. Nature.

[31] Barone MV, Pepperkok R, Peverali FA, Philipson L (1994). Id proteins control growth induction in mammalian cells. Proc. Natl Acad. Sci. USA.

[32] Hara E (1994). Id-related genes encoding helix-loop-helix proteins are required for G1 progression and are repressed in senescent human fibroblasts. J. Biol. Chem..

[33] Vassilev LT (2006). Selective small-molecule inhibitor reveals critical mitotic functions of human CDK1. Proc. Natl Acad. Sci. USA.

[34] Hara E, Hall M, Peters G (1997). Cdk2-dependent phosphorylation of Id2 modulates activity of E2A-related transcription factors. EMBO J..

[35] Soares MAF (2021). Hierarchical reactivation of transcription during mitosis-to-G1 transition by Brn2 and Ascl1 in neural stem cells. Genes Dev..

[36] Zhao J, Li X, Guo M, Yu J, Yan C (2016). The common stress responsive transcription factor ATF3 binds genomic sites enriched with p300 and H3K27ac for transcriptional regulation. BMC Genom..

[37] Zhao Q (2019). TCF21 and AP-1 interact through epigenetic modifications to regulate coronary artery disease gene expression. Genome Med..

[38] Casey AK, Chen S, Novick P, Ferro-Novick S, Wente SR (2015). Nuclear pore complex integrity requires Lnp1, a regulator of cortical endoplasmic reticulum. Mol. Biol. Cell.

[39] Zaidi SK (2018). Mitotic gene bookmarking: an epigenetic program to maintain normal and cancer phenotypes. Mol. Cancer Res..

[40] Foltz DR (2009). Centromere-specific assembly of CENP-a nucleosomes is mediated by HJURP. Cell.

[41] Dunleavy EM (2009). HJURP is a cell-cycle-dependent maintenance and deposition factor of CENP-A at centromeres. Cell.

[42] Bai C, Richman R, Elledge SJ (1994). Human cyclin F. EMBO J..

[43] Clijsters L (2019). Cyclin F controls cell-cycle transcriptional outputs by directing the degradation of the three activator E2Fs. Mol. Cell.

[44] Malumbres M, Barbacid M (2009). Cell cycle, CDKs and cancer: a changing paradigm. Nat. Rev. Cancer.

[45] Cappell SD (2018). EMI1 switches from being a substrate to an inhibitor of APC/C(CDH1) to start the cell cycle. Nature.

[46] Miller JJ (2006). Emi1 stably binds and inhibits the anaphase-promoting complex/cyclosome as a pseudosubstrate inhibitor. Genes Dev..

[47] Chen D, Zhao M, Mundy GR (2004). Bone morphogenetic proteins. Growth Factors.

[48] Varga AC, Wrana JL (2005). The disparate role of BMP in stem cell biology. Oncogene.

[49] Davidson KC (2012). Wnt/beta-catenin signaling promotes differentiation, not self-renewal, of human embryonic stem cells and is repressed by Oct4. Proc. Natl Acad. Sci. USA.

[50] Merrill BJ (2012). Wnt pathway regulation of embryonic stem cell self-renewal. Cold Spring Harb. Perspect. Biol..

[51] Basler K, Struhl G (1994). Compartment boundaries and the control of Drosophila limb pattern by hedgehog protein. Nature.

[52] Heemskerk J, DiNardo S (1994). Drosophila hedgehog acts as a morphogen in cellular patterning. Cell.

[53] Varjosalo M, Taipale J (2008). Hedgehog: functions and mechanisms. Genes Dev..

[54] Sasai Y, Kageyama R, Tagawa Y, Shigemoto R, Nakanishi S (1992). Two mammalian helix-loop-helix factors structurally related to Drosophila hairy and enhancer of split. Genes Dev..

[55] Akazawa C, Sasai Y, Nakanishi S, Kageyama R (1992). Molecular characterization of a rat negative regulator with a basic helix-loop-helix structure predominantly expressed in the developing nervous system. J. Biol. Chem..

[56] Kageyama R, Ohtsuka T, Kobayashi T (2007). The Hes gene family: repressors and oscillators that orchestrate embryogenesis. Development.

[57] McGinnis W, Krumlauf R (1992). Homeobox genes and axial patterning. Cell.

[58] Duverger O, Morasso MI (2008). Role of homeobox genes in the patterning, specification, and differentiation of ectodermal appendages in mammals. J. Cell. Physiol..

[59] Bhatlekar S, Fields JZ, Boman BM (2018). Role of HOX genes in stem cell differentiation and cancer. Stem Cells Int..

[60] Takashima Y, Suzuki A (2013). Regulation of organogenesis and stem cell properties by T-box transcription factors. Cell. Mol. Life Sci..

[61] Papaioannou VE (2014). The T-box gene family: emerging roles in development, stem cells and cancer. Development.

[62] Herrmann BG, Labeit S, Poustka A, King TR, Lehrach H (1990). Cloning of the T gene required in mesoderm formation in the mouse. Nature.

[63] Borgne A, Ostvold AC, Flament S, Meijer L (1999). Intra-M phase-promoting factor phosphorylation of cyclin B at the prophase/metaphase transition. J. Biol. Chem..

[64] Hagting A, Jackman M, Simpson K, Pines J (1999). Translocation of cyclin B1 to the nucleus at prophase requires a phosphorylation-dependent nuclear import signal. Curr. Biol..

[65] Rodier G, Coulombe P, Tanguay PL, Boutonnet C, Meloche S (2008). Phosphorylation of Skp2 regulated by CDK2 and Cdc14B protects it from degradation by APC(Cdh1) in G1 phase. EMBO J..

[66] Yamano, H. APC/C: current understanding and future perspectives. *F1000Res* **8**, 10.12688/f1000research.18582.1 (2019). 10.12688/f1000research.18582.1 PMC6534075 31164978

[67] Alfieri C, Tischer T, Barford D (2020). A unique binding mode of Nek2A to the APC/C allows its ubiquitination during prometaphase. EMBO Rep..

[68] Sedgwick GG (2013). Mechanisms controlling the temporal degradation of Nek2A and Kif18A by the APC/C-Cdc20 complex. EMBO J..

[69] Wolthuis R (2008). Cdc20 and Cks direct the spindle checkpoint-independent destruction of cyclin A. Mol. Cell.

[70] Zhang S, Tischer T, Barford D (2019). Cyclin A2 degradation during the spindle assembly checkpoint requires multiple binding modes to the APC/C. Nat. Commun..

[71] Bairoch A, Bucher P, Hofmann K (1997). The PROSITE database, its status in 1997. Nucleic Acids Res..

[72] Kanemori Y, Uto K, Sagata N (2005). Beta-TrCP recognizes a previously undescribed nonphosphorylated destruction motif in Cdc25A and Cdc25B phosphatases. Proc. Natl Acad. Sci. USA.

[73] Cuende J, Moreno S, Bolanos JP, Almeida A (2008). Retinoic acid downregulates Rae1 leading to APC(Cdh1) activation and neuroblastoma SH-SY5Y differentiation. Oncogene.

[74] Stegmuller J (2006). Cell-intrinsic regulation of axonal morphogenesis by the Cdh1-APC target SnoN. Neuron.

[75] Egli D, Rosains J, Birkhoff G, Eggan K (2007). Developmental reprogramming after chromosome transfer into mitotic mouse zygotes. Nature.

[76] Ganier O (2011). Synergic reprogramming of mammalian cells by combined exposure to mitotic Xenopus egg extracts and transcription factors. Proc. Natl Acad. Sci. USA.

[77] Halley-Stott RP, Jullien J, Pasque V, Gurdon J (2014). Mitosis gives a brief window of opportunity for a change in gene transcription. PLoS Biol..

[78] Mendez J, Stillman B (2000). Chromatin association of human origin recognition complex, cdc6, and minichromosome maintenance proteins during the cell cycle: assembly of prereplication complexes in late mitosis. Mol. Cell Biol..

[79] Yamaguchi M (2015). Structure of an APC3–APC16 complex: insights into assembly of the anaphase-promoting complex/cyclosome. J. Mol. Biol..

[80] Chang L, Zhang Z, Yang J, McLaughlin SH, Barford D (2015). Atomic structure of the APC/C and its mechanism of protein ubiquitination. Nature.

[81] Sehnal, D. et al. Mol* Viewer: modern web app for 3D visualization and analysis of large biomolecular structures. *Nucleic Acids Res.*10.1093/nar/gkab314 (2021). 10.1093/nar/gkab314 PMC8262734 33956157

[82] He J (2013). Insights into degron recognition by APC/C coactivators from the structure of an Acm1–Cdh1 complex. Mol. Cell.

[83] Wong MV, Jiang S, Palasingam P, Kolatkar PR (2012). A divalent ion is crucial in the structure and dominant-negative function of ID proteins, a class of helix-loop-helix transcription regulators. PLoS ONE.

[84] Bray NL, Pimentel H, Melsted P, Pachter L (2016). Near-optimal probabilistic RNA-seq quantification. Nat. Biotechnol..

[85] Soneson C, Love MI, Robinson MD (2015). Differential analyses for RNA-seq: transcript-level estimates improve gene-level inferences. F1000Res..

[86] Fischer DS, Theis FJ, Yosef N (2018). Impulse model-based differential expression analysis of time course sequencing data. Nucleic Acids Res..

[87] Frattini V (2018). A metabolic function of FGFR3-TACC3 gene fusions in cancer. Nature.

