# Supplementary material for: Regulated interaction of ID2 with the anaphase-promoting complex links progression through mitosis with reactivation of cell-type-specific transcription
Source: Nat Commun. 2022 Apr 19;13:2089. doi: 10.1038/s41467-022-29502-2 (PMC9018835; doi:10.1038/s41467-022-29502-2)
Supplement: Supplementary file 1 — Supplementary Information [file 41467_2022_29502_MOESM1_ESM.pdf]

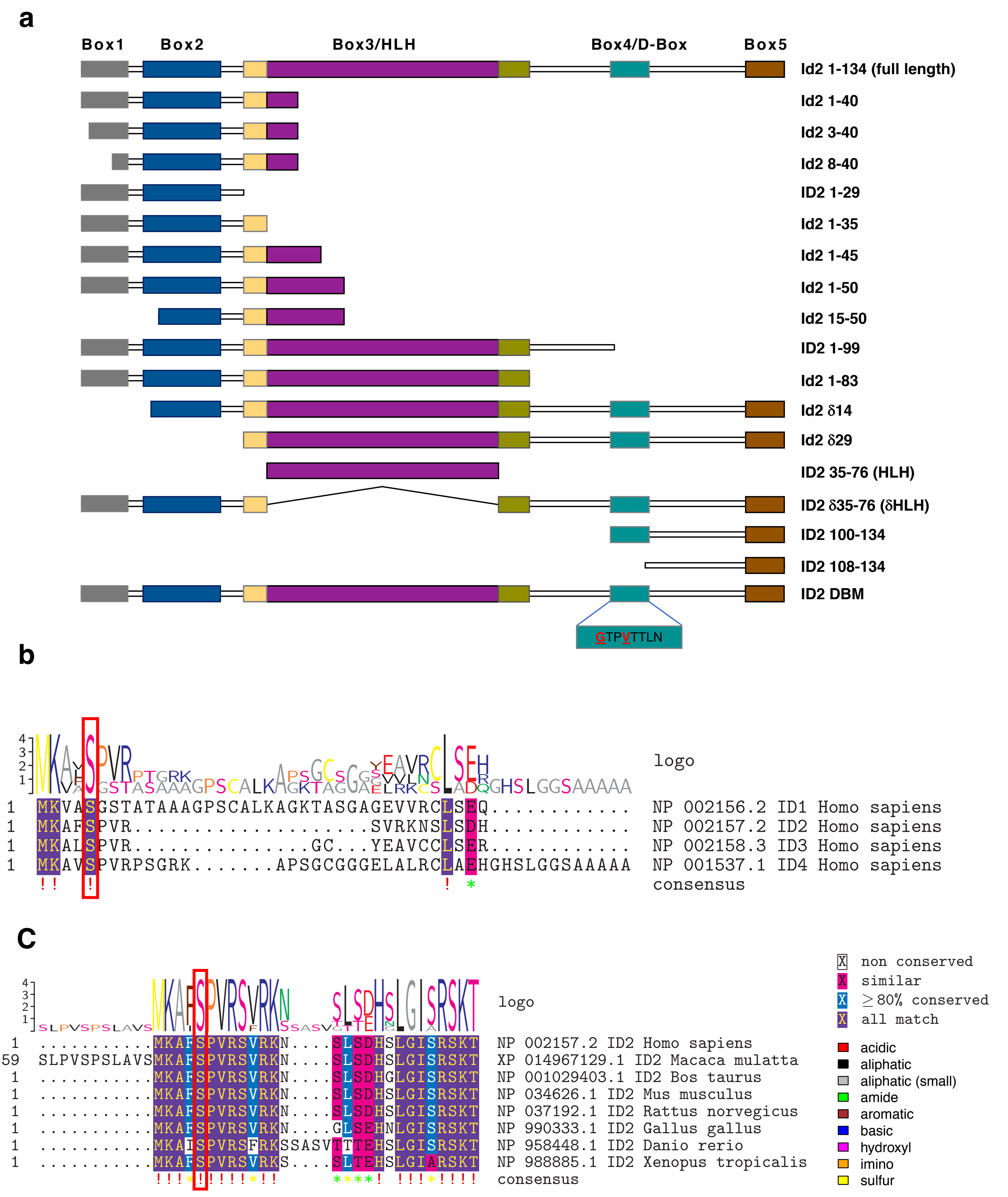

Supplementary Figure 1

Supplementary Fig. 1. **Mutations of ID2 protein and conservation of ID2 and ID family members. a**, Schematics of GST-ID2 mutant constructs included in experiments in Fig. 1. **b**, Serine 5 is conserved in human ID family members. **c**, Serine 5 of human ID2 is evolutionary conserved.

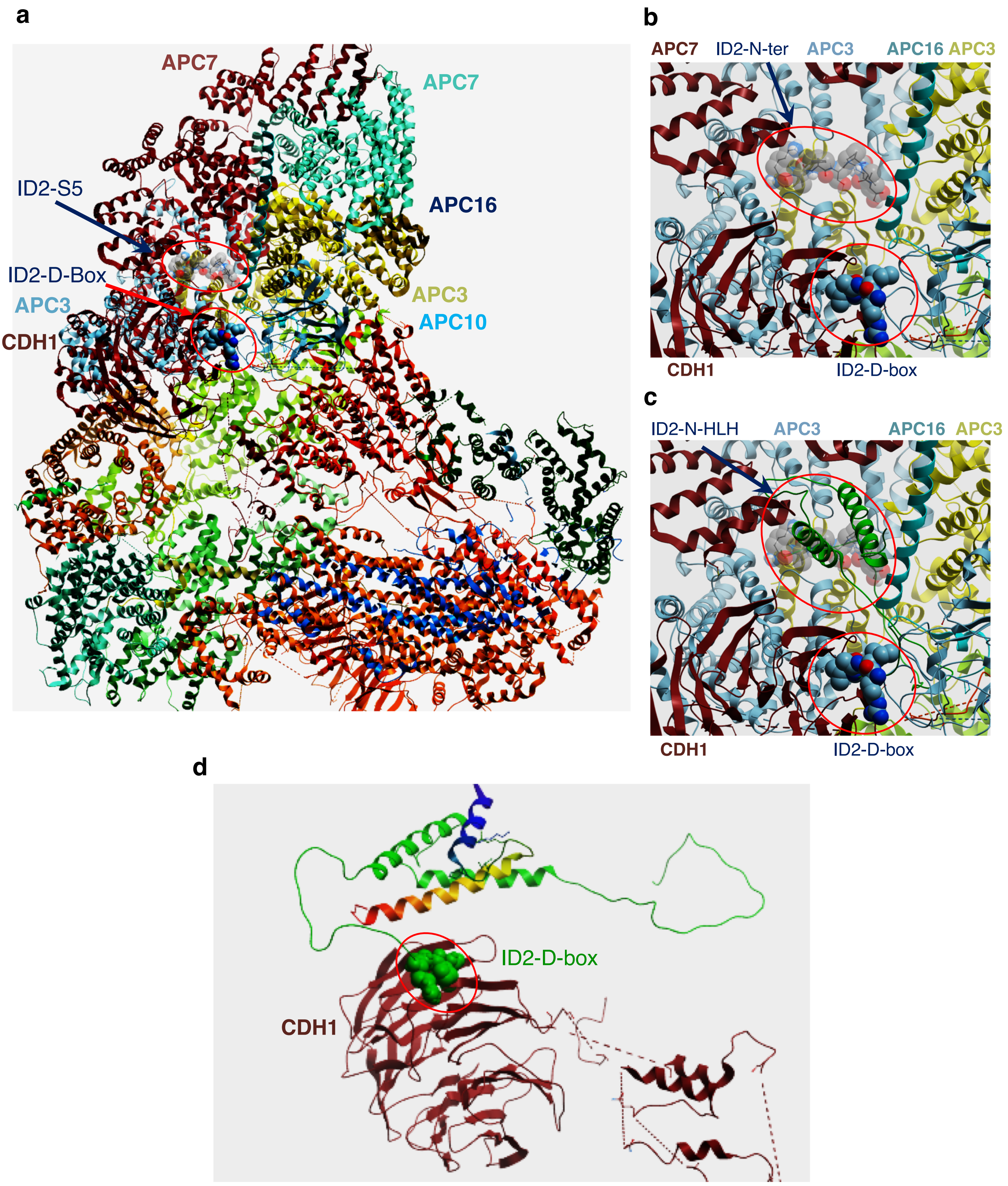

Supplementary Figure 2

Supplementary Fig. 2. Molecular docking of ID2 on the APC^{CDH1} complex. a-b, APC^{CDH1} complex (**a**) and close-up (**b**) view showing large cavity bounded by APC7 (maroon helices upper left in a and b; aqua helices upper right only visible in a), APC16 (long aqua helix in a and b), APC 10 (light blue ribbon central in a and lower right in b) and CDH1 (maroon beta-propeller). The ID2 D-box binding peptide is shown in solid grey (carbon), red (oxygen) and blue (nitrogen) spheres, while the ID2 N-terminal peptide containing S5 docked to APC3 is shown in transparent spheres with the same color scheme. APC3 homodimer is shown at the bottom of the cavity as grey and yellow ribbon. **c**, Same view as **b**, but with ID2 full length protein shown as green ribbon occupying the cavity with N-terminus bound to APC3, the two central helices of ID2 (HLH) in green and the C-terminal D-box bound to CDH1. **d**, ID2 4-helix bundle homodimer in its conformation plugging the cavity in holo-APC including ID2 2-106 (green) with N-terminus 2-8 peptide docked to APC3 and C-terminus D-box (green sphere) docked to CDH1 (red ribbon).

a

ID2 side chain	Contact Area with APC3 (Angstroms)	Closest APC3 Heavy Atom	Closest_Distance (Angstroms)
Arg8	147	Tyr21 hydroxyl	2.1
Lys2	81	Leu120 distal carbon	2.6
Phe4	54	Asp81 carboxyl oxygen	2.4

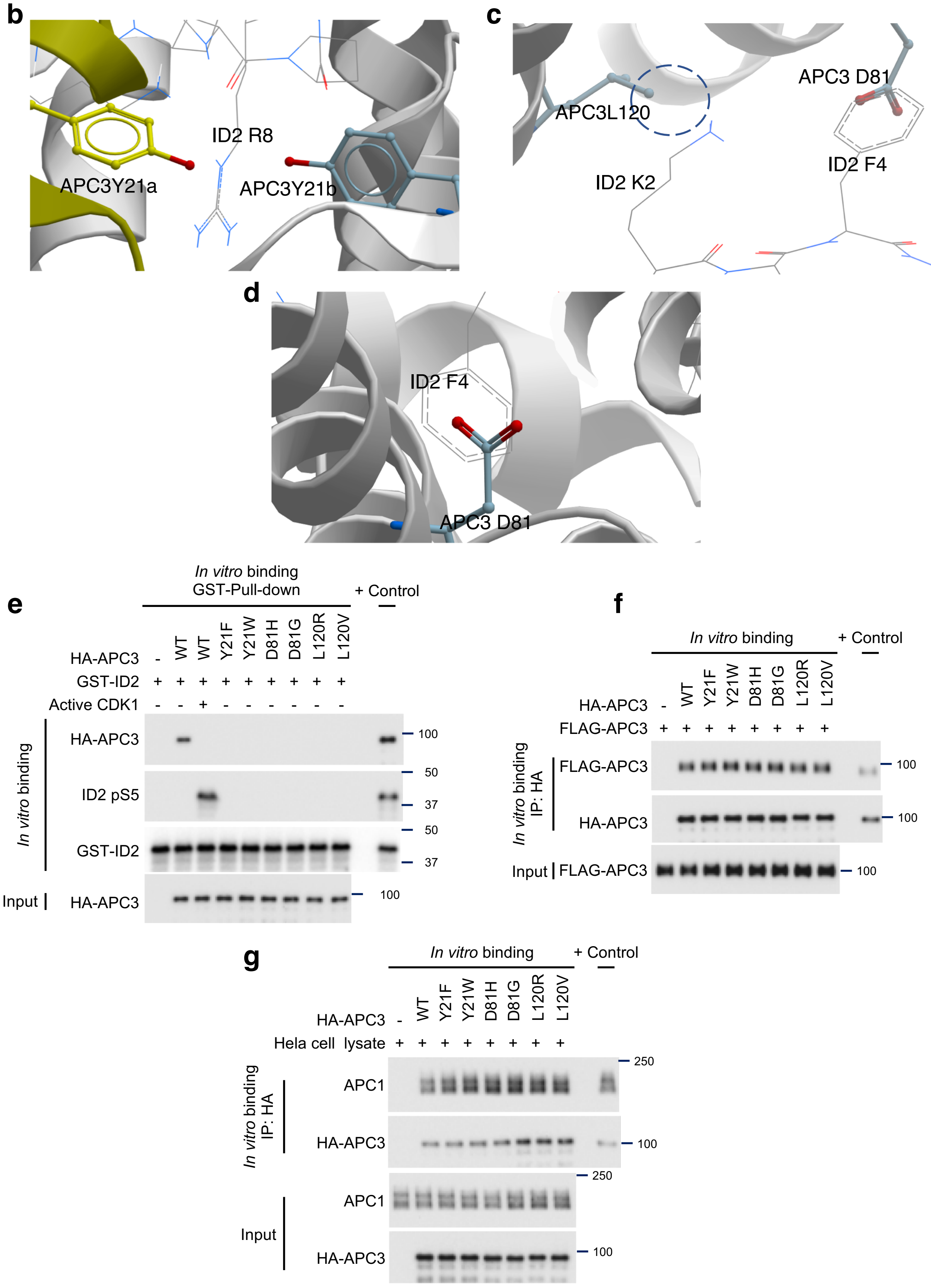

Supplementary Figure 3

Supplementary Fig. 3. APC3 Mutations at predicted ID2 interface abolish the interaction *in vitro*. a, Top ID2-contacting surface (not buried in the hydrophobic core or the APC3 homodimer interface) amino acid side-chains in APC3. **b**, Hydroxyl groups of tyrosine-21 (Tyr21, Y21) from both APC3 monomers in the dimer form hydrogen bonds with arginine-8 (Arg8, R8) of ID2 at the docked location within APC3. Mutation to either phenylalanine (F) or tryptophan (W) abolishes this electrostatic contact. **c**, APC3 Leucine-120 (Leu120, L120) forces the positively charged terminal amino group of ID2 lysine-2 (Lys2, K2) into a position where it can form a salt bridge with APC3 aspartic acid-81 (Asp-81, D81). Mutation of L120 to the smaller valine creates a cavity (dashed circle) which would likely disrupt the salt bridge, while mutation to arginine electrostatically repels ID2-K2, also potentially disrupting the salt bridge. **d**, The carboxyl group of APC3 D81 also forms an anion-π interaction with F4 of ID2 in the docked conformation, which is abolished by mutation to either H or G. **e**, *In vitro* binding between ID2 and APC3 WT and mutants was performed using *in vitro* translated HA-APC3 polypeptides and GST-ID2. After GST pull-down, precipitated proteins were analyzed by western blot. GST-ID2 phosphorylated in vitro by active CDK1 was included. **f**, *In vitro* binding between *in vitro* translated HA-APC3 WT and mutants and FLAG-APC3 WT expressed in HeLa cells. Immunoprecipitation was performed using HA affinity matrix. Precipitated proteins were analyzed by western blot. **g**, *In vitro* binding between *in vitro* translated HA-APC3 WT and mutants and endogenous APC1 from HeLa cell lysates. Immunoprecipitation was performed using HA affinity matrix. Precipitated proteins were analyzed by western blot. Molecular weight markers are indicated in kDa. + Control are aliquots of *in vitro* translation of APC3 wild type or CDK1 *in vitro* kinase reaction (e, f, g), and HeLa cell lysates (f, g). HA-APC3, FLAG-APC3 and pS5-ID2 are from independent gels; APC1 and APC3 in panel g are from same gel. Experiments were repeated two times with similar results.

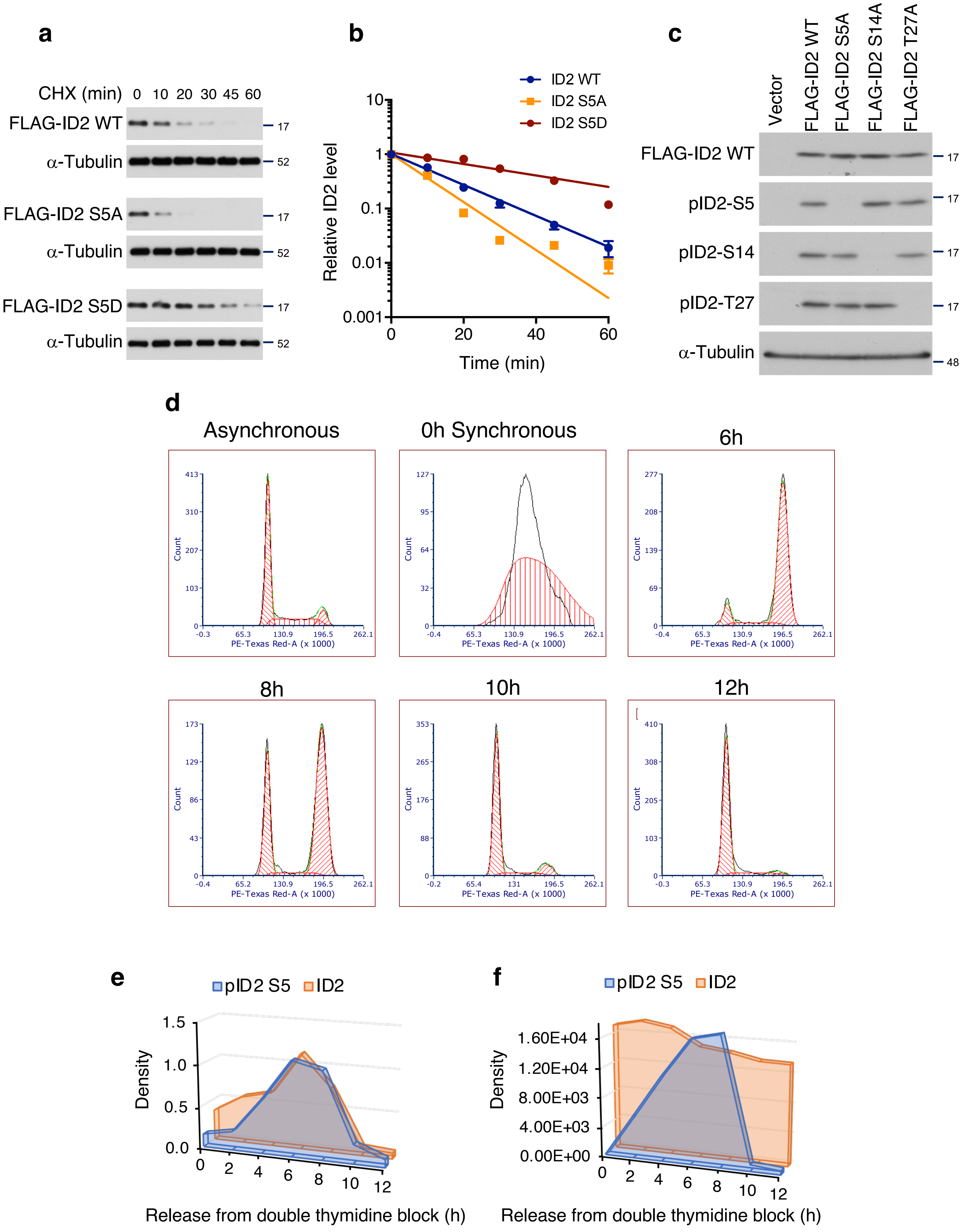

Supplementary Figure 4

Supplementary Fig. 4. Constitutive phosphorylation of S5 extends ID2 half-life and peaks at mitosis. **a**, Half-life of ID2 wild type and phospho-mutants as determined in U251 cells treated with CHX for the indicated times. **b**, Quantification of data in a. Data are means +/- SEM of two independent experiments. **c**, Validation of ID2 phospho-specific antibodies. Cells were transfected with vectors expressing FLAG-tagged ID2 wild type, ID2-S5A, ID2-S14A, and ID2-T27A. The western blot indicates the specificity of each antibody for the corresponding phosphosite of ID2. **d**, Representative cell cycle profile of HeLa cells synchronized by double thymidine block and released at the indicated times. **e, f**, Quantification of ID2 and phospho-S5-ID2 in total lysates (e) and immunoprecipitates (f) of HeLa cells released from double thymidine block as shown in Fig. 3d, e. Experiments were repeated three times with similar results.

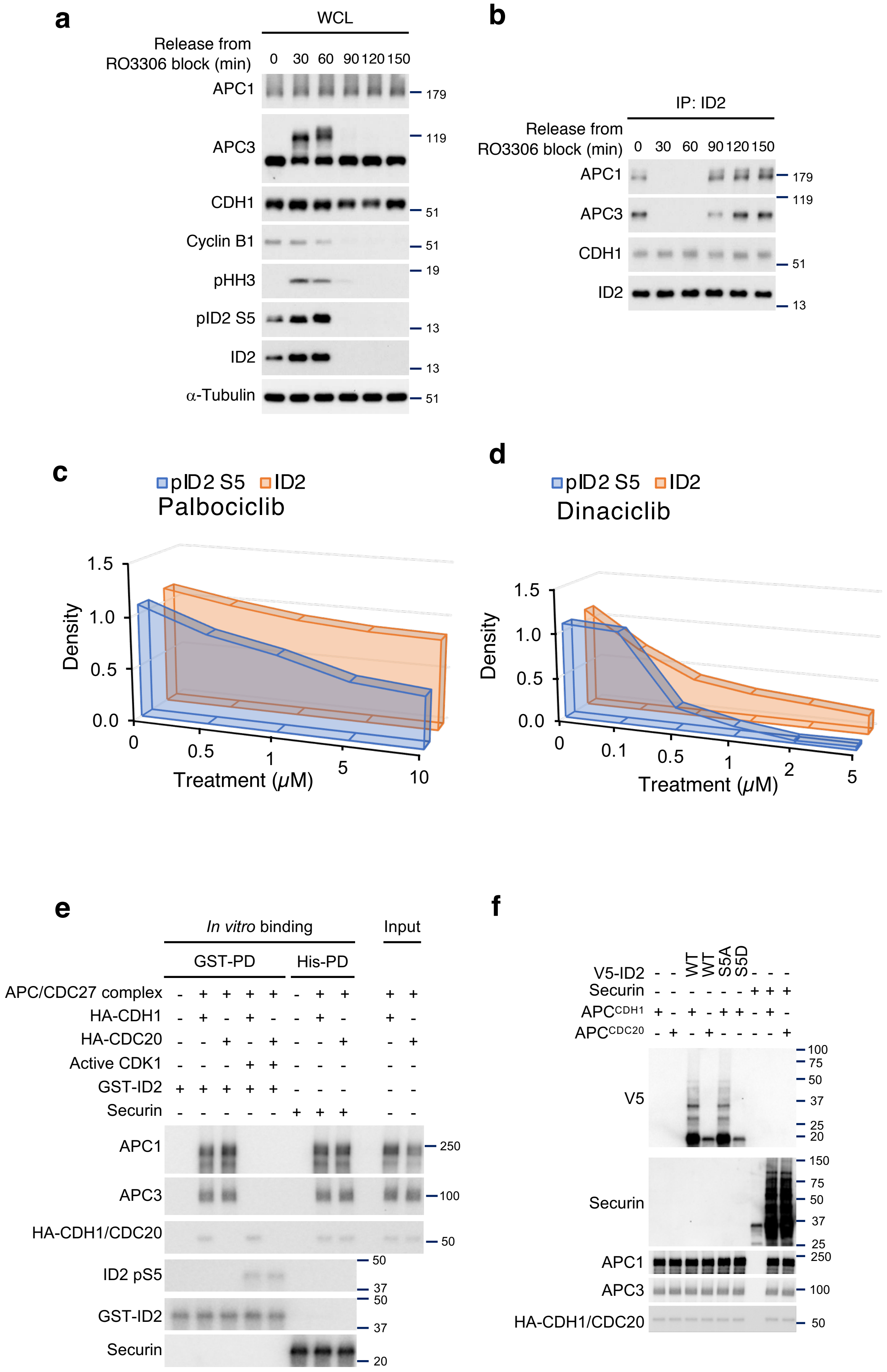

Supplementary Figure 5

Supplementary Fig. 5. Analysis of cells synchronized in G2/M phase of the cell cycle and validation of APC^{CDC20} activity. a, Western blot analysis of lysates from cells synchronized at the G2/M phase of the cell cycle using RO3306 and released at the indicated times. **b**, Analysis of the binding between endogenous ID2 and APC^{CDH1} complex during transition from G2-M to G1 phase of the cell cycle as shown in a. Immunoprecipitation was performed using ID2 antibody followed by western blot for APC1, APC3, and CDH1. **c, d** Quantification of ID2 and phospho-S5-ID2 in total lysates of HeLa cells treated with palbociclib or dinaciclib as shown in Fig. 3f. **e**, Core APC is unable to bind ID2 phosphorylated by CDK1-Cyclin B but is still proficient for the interaction with securin. GST-ID2 was phosphorylated *in vitro* by CDK1-Cyclin B or mock treated before undergoing binding *in vitro* with immunopurified FLAG-APC3 that had been expressed in HeLa cells and activated by *in vitro* translated HA-CDC20 or CDH1. 6x-histidine-tagged securin was included as control for the binding capacity of APC^{CDC20}. Proteins co-precipitated by GST or 6x-histidine pull-down were detected by western blot. **f**, Inability of APC^{CDH1} to ubiquitylate the ID2-S5 phospho-mimetic mutant S5D. *In vitro* ubiquitylation of V5-ID2 wild type and phosphorylation mutants was performed using the APC complex purified and activated as in **e**. Ubiquitylation of recombinant securin was tested as control for the enzymatic activity of APC^{CDC20}. Molecular weight markers are indicated in kDa. APC1, APC3, CDH1/CDC20 are from the same gel in each panel; Cyclin B, total ID2, pS5-ID2, GST-ID2, securin, and V5 are from different gels. In panel a, loading control is from the same gel as ID2. Molecular weight markers are indicated in kDa. Experiments were repeated two times with similar results.

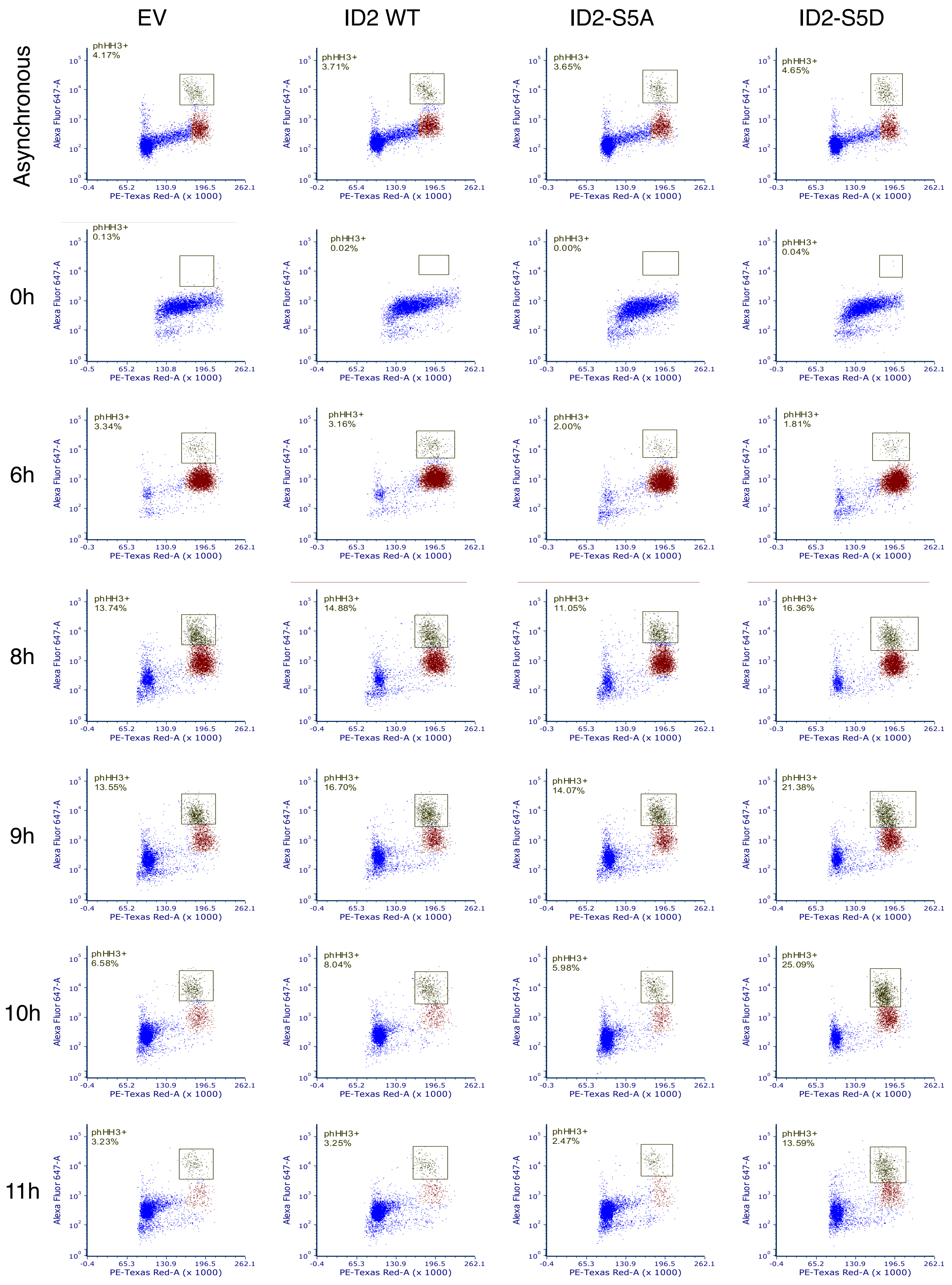

Supplementary Figure 6

Supplementary Fig. 6. Quantitative analysis of mitotic progression in HeLa cells expressing ID2 wild type, ID2-S5A, ID2 S5D or the empty vector. Cells were synchronized by double thymidine block and released at the indicated times. Cells were fixed, immunostained using the antibody against the phosphorylated Ser-10 of histone H3 (phHH3), and analyzed by flow cytometry. Experiments were repeated four times with similar results.

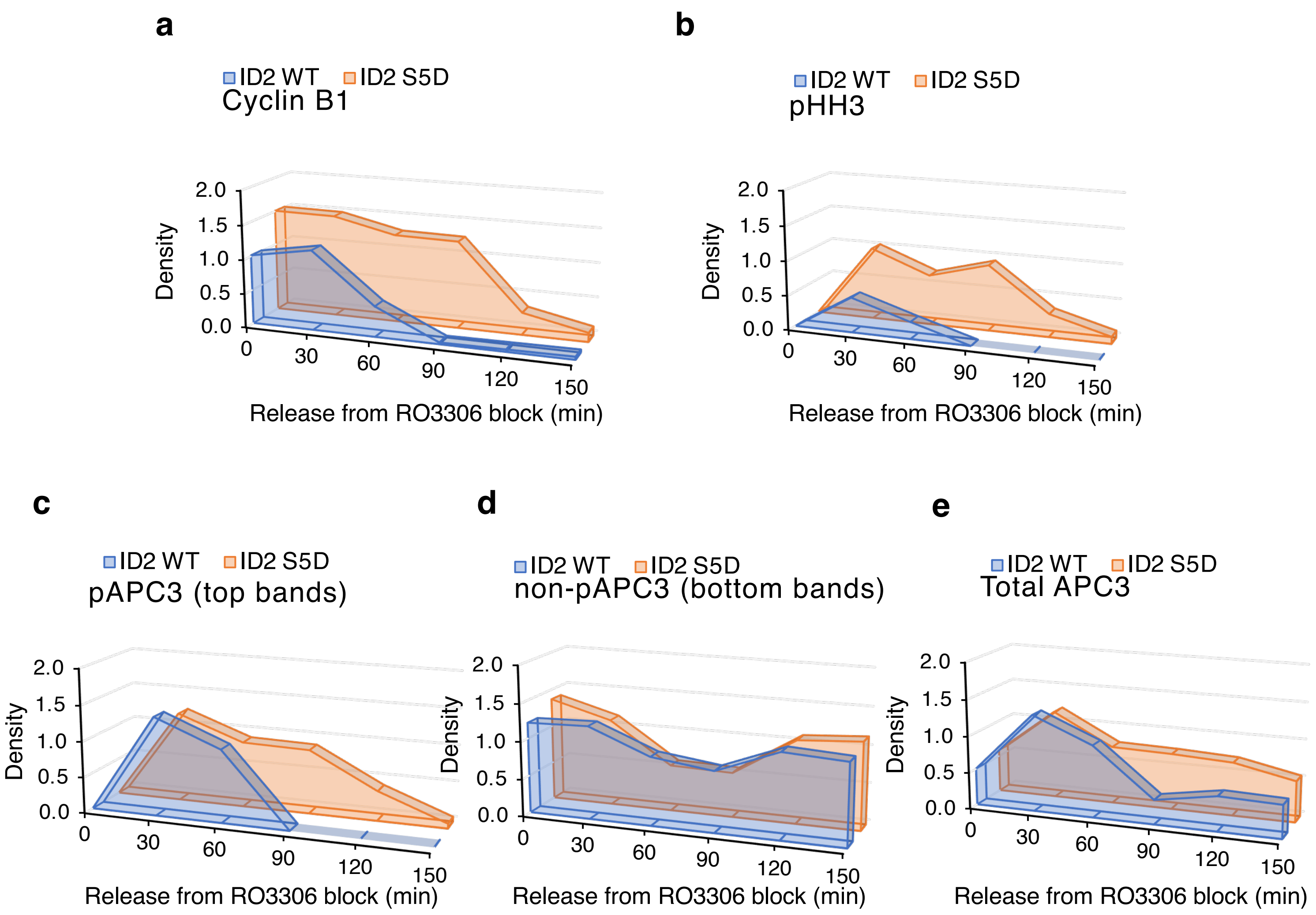

Supplementary Figure 7

Supplementary Fig. 7. Quantitative analysis of mitotic proteins and APC3 in HeLa cells expressing ID2 wild type or ID2 S5D. Cells were synchronized by treatment with RO3306 and released at the indicated times as in Fig. 4d.

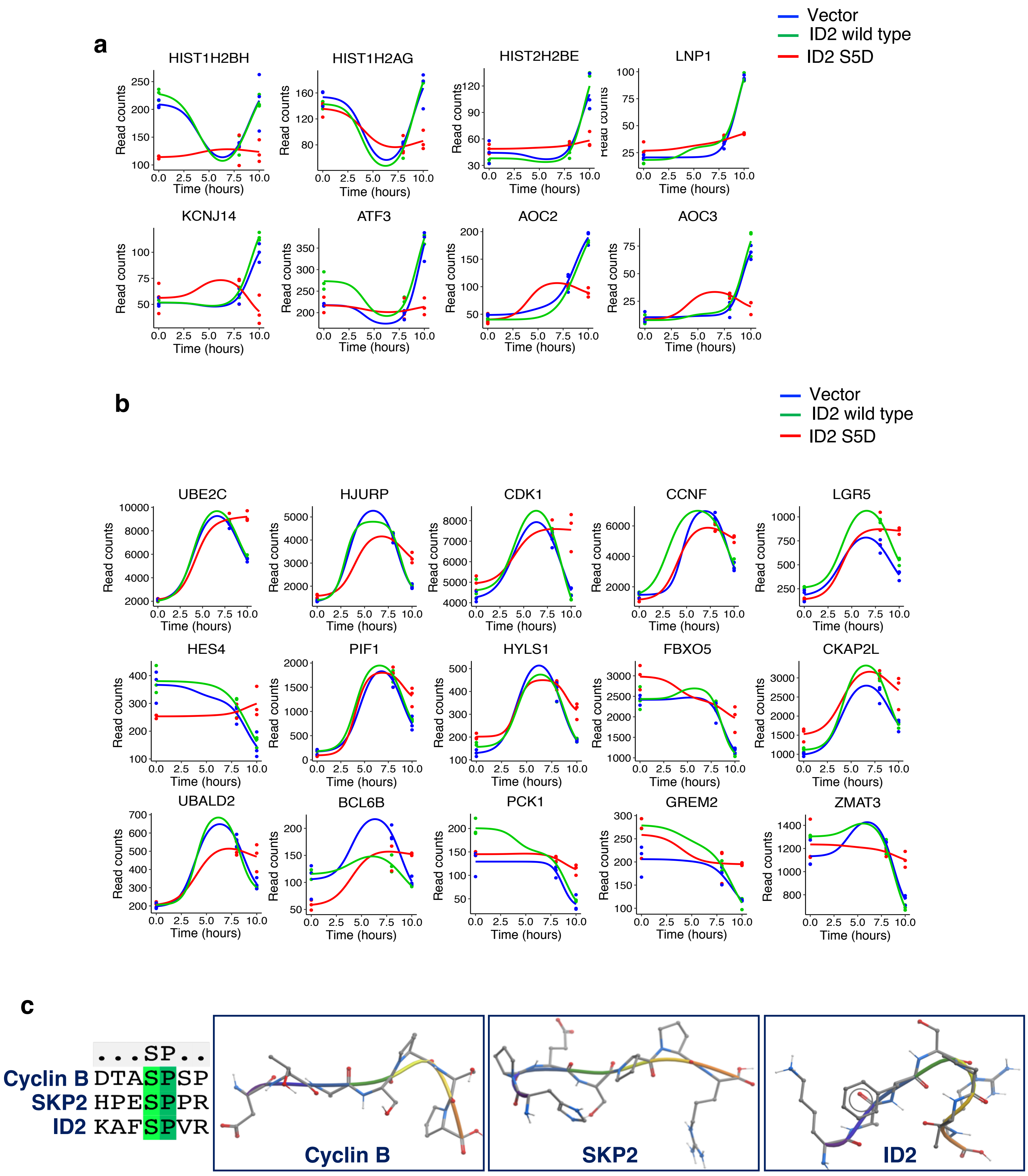

Supplementary Figure 8

Supplementary Fig. 8. Time series transcriptomic analysis of cells expressing ID2 Ser-5D and modeling of CDK1 phosphorylated serine in ID2, SKP2, and Cyclin B. **a**, Time series analysis of representative genes downregulated at exit from mitosis in cells expressing ID2 Ser-5D. **b**, Time series analysis of representative genes upregulated at exit from mitosis in cells expressing ID2 Ser-5D. Transcriptomic profiles were obtained from three biological samples. **c**, Sequence of the 7 amino acid residues surrounding CDK1 phosphorylated serine in Cyclin B, SKP2 and ID2 (left); 3-D structure of the same sequences centered on the phosphorylated serine, which are located in disordered flexible regions of Cyclin B, SKP2 and ID2, shows similarity of the three peptides (right).

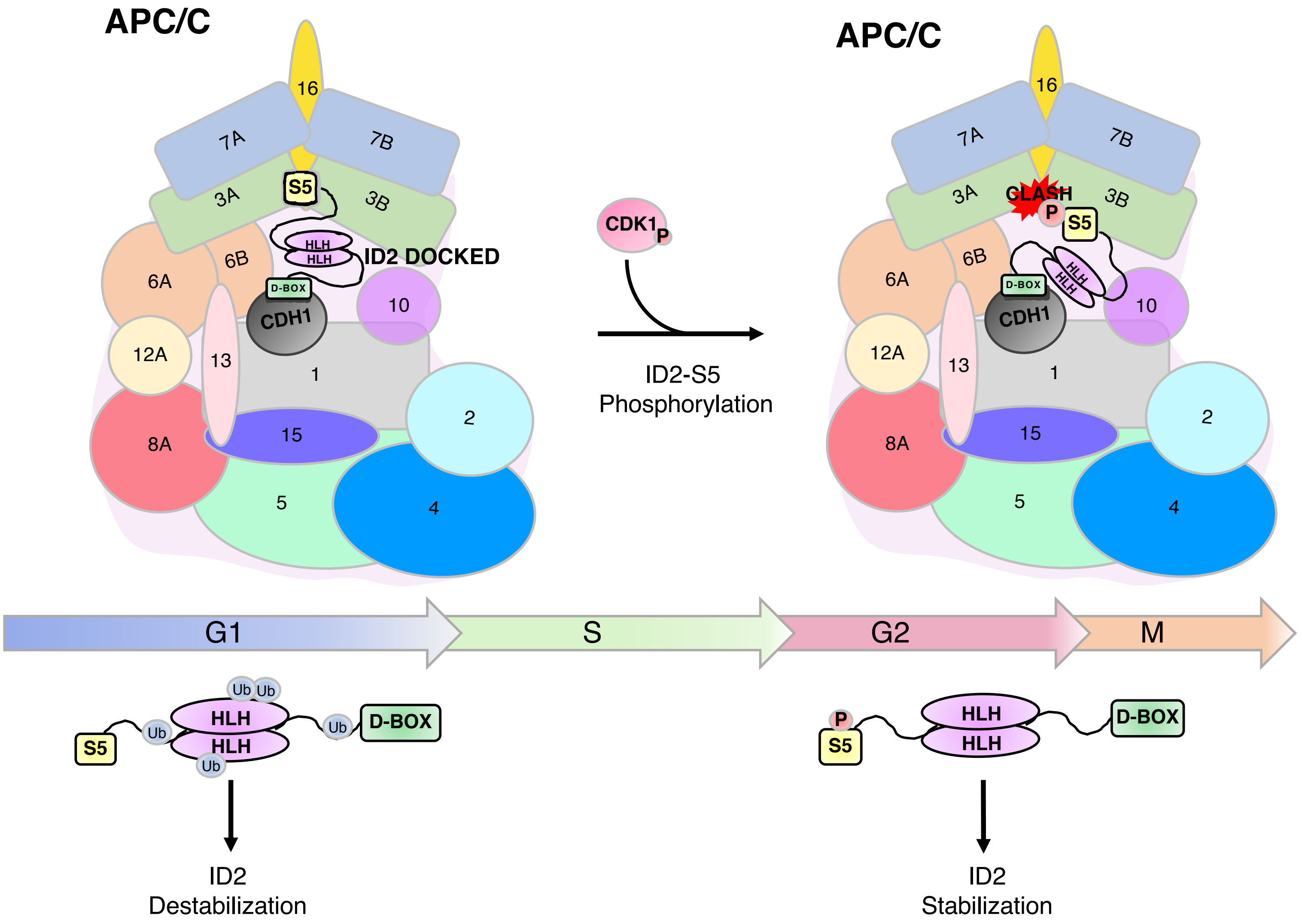

Supplementary Figure 9

Supplementary Fig. 9. Schematic model of ID2-APC^{CDH1} interaction during cell cycle. Left, after mitotic exit, at the G1 phase of the cell cycle, ID2 interacts with CDH1 through the C-terminal D-box and associates with core APC via the N-terminal containing unphosphorylated S5. S5-ID2 binds to APC within a pocket mainly constituted by the APC3 homodimer. The dual interaction of ID2 with core APC and CDH1 causes optimal ID2 ubiquitylation and destruction. As cell cycle progresses and Cyclin B-CDK1 becomes activated, CDK1-mediated phosphorylation of S5 of ID2 prevents binding to APC3 resulting in inefficient ID2 ubiquitylation and consequent stabilization.
