# Supplementary material for: Regulated interaction of ID2 with the anaphase-promoting complex links progression through mitosis with reactivation of cell-type-specific transcription
Source: Nat Commun. 2022 Apr 19;13:2089. doi: 10.1038/s41467-022-29502-2 (PMC9018835; doi:10.1038/s41467-022-29502-2)
Supplement: Supplementary file 2 — Description of Additional Supplementary Files [file 41467_2022_29502_MOESM2_ESM.pdf]

Description of Additional Supplementary Files

File Name: Supplementary Data 1
Description: Differential expression analysis between ID2 Ser-5D versus wt and empty vector.

File Name: Supplementary Data 2
Description: Gene ontology enrichment analysis between ID2 Ser-5D versus wt expressing samples collected 10 h after double thymidine block.

File Name: Supplementary Data 3
Description: ImpulseDE2 analysis between ID2 Ser-5D versus wt expressing samples collected 10 h after double thymidine block.
