# Supplementary material for: Regulated interaction of ID2 with the anaphase-promoting complex links progression through mitosis with reactivation of cell-type-specific transcription
Source: Nat Commun. 2022 Apr 19;13:2089. doi: 10.1038/s41467-022-29502-2 (PMC9018835; doi:10.1038/s41467-022-29502-2)
Supplement: Supplementary file 4 — Reporting Summary [file 41467_2022_29502_MOESM4_ESM.pdf]

nature research

Corresponding author(s): Anna Lasorella, Antonio Iavarone

Last updated by author(s): Mar 9, 2022

Reporting Summary

Nature Research wishes to improve the reproducibility of the work that we publish. This form provides structure for consistency and transparency in reporting. For further information on Nature Research policies, see our Editorial Policies and the Editorial Policy Checklist.

Statistics

For all statistical analyses, confirm that the following items are present in the figure legend, table legend, main text, or Methods section.

n/a	Confirmed	
☐	☒	The exact sample size (*n*) for each experimental group/condition, given as a discrete number and unit of measurement
☐	☒	A statement on whether measurements were taken from distinct samples or whether the same sample was measured repeatedly
☐	☒	The statistical test(s) used AND whether they are one- or two-sided<br>*Only common tests should be described solely by name; describe more complex techniques in the Methods section.*
☐	☒	A description of all covariates tested
☐	☒	A description of any assumptions or corrections, such as tests of normality and adjustment for multiple comparisons
☐	☒	A full description of the statistical parameters including central tendency (e.g. means) or other basic estimates (e.g. regression coefficient) AND variation (e.g. standard deviation) or associated estimates of uncertainty (e.g. confidence intervals)
☐	☒	For null hypothesis testing, the test statistic (e.g. *F*, *t*, *r*) with confidence intervals, effect sizes, degrees of freedom and *P* value noted<br>*Give P values as exact values whenever suitable.*
☒	☐	For Bayesian analysis, information on the choice of priors and Markov chain Monte Carlo settings
☒	☐	For hierarchical and complex designs, identification of the appropriate level for tests and full reporting of outcomes
☒	☐	Estimates of effect sizes (e.g. Cohen's *d*, Pearson's *r*), indicating how they were calculated

Our web collection on statistics for biologists contains articles on many of the points above.

Software and code

Policy information about availability of computer code

Data collection	No software was used
Data analysis	GraphPad Prism v.6.0<br>ImpulseDE2 v.1.8.0<br>kallisto v.0.46.0<br>R v.3.4.4<br>tximport v.1.20.0<br>yaGST v.1<br>bd facsdiva v.7.0.1<br>FCS Express 6 Flow v.7.12.007<br>FlowJo v.10.7.1<br>ICM-Pro, Molsoft LLC

For manuscripts utilizing custom algorithms or software that are central to the research but not yet described in published literature, software must be made available to editors and reviewers. We strongly encourage code deposition in a community repository (e.g. GitHub). See the Nature Research guidelines for submitting code & software for further information.

Data

Policy information about availability of data

All manuscripts must include a data availability statement. This statement should provide the following information, where applicable:

- Accession codes, unique identifiers, or web links for publicly available datasets
- A list of figures that have associated raw data
- A description of any restrictions on data availability

RNA-Seq profiling of 27 samples (3 cell conditions: Hela cells transduced with ID2 S5D, ID2 WT, EV; 3 time points: 0 h, 8 h, 10 h; 3 replicates each) have been submitted to GEO (raw counts and fastq data; accession no. GSE181639). The remaining data are available within the Article and Supplementary Information. Source data are provided with this paper.
The list of figures that have associated biological raw data are:
Figure 1;
Figure 3;
Figure 4;
Figure 5;
Figure 6;
Supplementary Figure 3e, f, g;
Supplementary Figure 4a, b, c;
Supplementary Figure 5a, b, e, f;
Supplementary Figure 6.

Field-specific reporting

Please select the one below that is the best fit for your research. If you are not sure, read the appropriate sections before making your selection.

☒ Life sciences ☐ Behavioural & social sciences ☐ Ecological, evolutionary & environmental sciences

For a reference copy of the document with all sections, see nature.com/documents/nr-reporting-summary-flat.pdf

Life sciences study design

All studies must disclose on these points even when the disclosure is negative.

Sample size	No statistical methods were used to predetermine sample size. All available samples passing the quality control were included.
Data exclusions	No data were excluded.
Replication	Three technical replicates were performed and experiments were repeated at least three times with similar results. All attempts at replication were successful.
Randomization	No experimental grouping requiring randomization was performed.
Blinding	Molecular classification was performed independent of and blinded to the clinical features. Investigators were blinded to the clinical and molecular features during experiments and outcome assessments.

Reporting for specific materials, systems and methods

We require information from authors about some types of materials, experimental systems and methods used in many studies. Here, indicate whether each material, system or method listed is relevant to your study. If you are not sure if a list item applies to your research, read the appropriate section before selecting a response.

Materials & experimental systems

n/a	Involved in the study	
☐	☒	Antibodies
☐	☒	Eukaryotic cell lines
☒	☐	Palaeontology and archaeology
☒	☐	Animals and other organisms
☒	☐	Human research participants
☒	☐	Clinical data
☒	☐	Dual use research of concern

Methods

n/a	Involved in the study	
☒	☐	ChIP-seq
☐	☒	Flow cytometry
☒	☐	MRI-based neuroimaging

Antibodies

Antibodies used	ID2 1:500 (C-20, #sc-489), E2A/E47 1:1000 (N-649, #sc-763) and CDH1/Fzr 1:250 (DCS-266, #sc-56312) obtained from Santa Cruz Biotechnology; HA 1:1000 (C29F4, #3724 or #2367), phospho-SKP2 1:1000 (Ser64) (#14865), SKP2 1:1000 (D3G5, #2652), CCNB1 1:1000 (D5C10, #12231), APC1 1:1000 (D1E9D, #13329), APC3/CCDC27 1:1000 (D3I1V, #12530), TCF12/HEB 1:1000 (D2C10, #11825), securin1:1,000 (D2B6O #13455) obtained from Cell Signaling Technology; β-actin 1:8000 (#A5441), alpha-tubulin 1:8000 (#T5168), vinculin 1:5000 (#V9131), and Flag M2 1:500 (#F1804) obtained from Sigma; HA 1:1000 (3F10, #12158167001) obtained from Roche; phospho-Cyclin B1 (S126) 1:1000 (#ab55184) obtained from Abcam. Secondary antibodies horseradish-peroxidase-conjugated Thermofisher Scientific: anti-mouse 1:10,000 (#32460); anti rabbit 1:10,000 (#32430), anti-rat 1:10,000 (#31470) .
Validation	Antibodies used were commercially available and were validated in multiple previous studies. The following are the Research Resource Identifiers (RRIDs) from the Resource Identification Portal, supporting guidelines for Rigor and Transparency in scientific publications.<br>FLAG, RRID:AB_262044.<br>HRP-conjugated goat-anti-mouse secondary, RRID:AB_228217.<br>ID2, RRID:AB_2122883.<br>E2A/E47, RRID:AB_631405.<br>CDH1/Fzr, RRID:AB_783404.<br>HA, RRID:AB_1549585.<br>phospho-SKP2, RRID:AB_2798634.<br>SKP2, RRID:AB_11178941.<br>CCNB1, RRID:AB_2783553.<br>APC1, RRID:AB_2798182.<br>APC3/CCDC27, RRID:AB_2797944.<br>TCF12/HEB, RRID:AB_2797736.<br>securin, RRID:AB_2798220.<br>beta-actin, RRID:AB_476744.<br>alpha-tubulin, RRID:AB_477579.<br>vinculin, RRID:AB_477629.<br>Flag M2, RRID:AB_262044.<br>HA, RRID:AB_390915.<br>phospho-Cyclin B1 (S126), RRID:AB_879764.<br>Phospho-Histone H3, RRID:AB_10694086.

Eukaryotic cell lines

Policy information about cell lines

Cell line source(s)	HeLa (ATCC, CCL-2)<br>HEK293T (ATCC, CRL-11268)<br>U-251 MG (Millipore-Sigma # 09063001)<br>U-2 OS (American Type Culture Collection, HTB-96)
Authentication	Cell authentication was performed using short tandem repeats (STR) at the ATCC facility.
Mycoplasma contamination	Cells were routinely tested for mycoplasma contamination using Mycoplasma Plus PCR Primer Set (Agilent, Santa Clara, CA) and were found to be negative.
Commonly misidentified lines (See ICLAC register)	We have not used cell lines listed in the database of commonly misidentified cell lines.

Flow Cytometry

Plots

Confirm that:

- [x] The axis labels state the marker and fluorochrome used (e.g. CD4-FITC).
- [x] The axis scales are clearly visible. Include numbers along axes only for bottom left plot of group (a 'group' is an analysis of identical markers).
- [x] All plots are contour plots with outliers or pseudocolor plots.
- [x] A numerical value for number of cells or percentage (with statistics) is provided.

Methodology

Sample preparation	Cells were harvested by trypsinization, washed in PBS and fixed in cold 70% ethanol overnight. Fixed cells were washed twice in PBS, treated with ribonuclease A 100g/ml in PBS plus 0.1% Triton X-100 and stained with propidium iodide at concentration of 50 mg/ml for at least 2 hours before the analysis.

	For phospho-histone-H3 staining, cells harvested and fixed in cold 70% ethanol were permeabilized in PBS containing 0.25% triton X-100 for 15 minutes, washed in PBS containing 1% bovine serum albumin and immunostained using Alexa Fluor 647-conjugated histone-H3 phospho-Ser-10 antibody (1:50; Cell Signaling Technology #3458) in PBS containing 1% bovine serum albumin for 1 hour at room temperature. After tree washes in PBS, cells were stained with propidium iodide in PBS containing 100g/ml ribonuclease A for 30 minutes.
Instrument	BD FACSCalibur and LSR II Flow Cytometer (BD Biosciences, San Jose, CA) on the basis of forward and sideward scatter parameters and Texas red or Alexa 647 fluorescence.
Software	BD FACSDiva Software v.7.0.1 for acquisition; FlowJo v.10.7.1 or FCS Express 6 Flow v.7.12.007 for analysis.
Cell population abundance	At least 10,000 cells for each sample were evaluated.
Gating strategy	The gating strategy includes all viable cells and singlets.

☒ Tick this box to confirm that a figure exemplifying the gating strategy is provided in the Supplementary Information.
