# Supplementary material for: Regulated interaction of ID2 with the anaphase-promoting complex links progression through mitosis with reactivation of cell-type-specific transcription
Source: Nat Commun. 2022 Apr 19;13:2089. doi: 10.1038/s41467-022-29502-2 (PMC9018835; doi:10.1038/s41467-022-29502-2)

## Slide 1
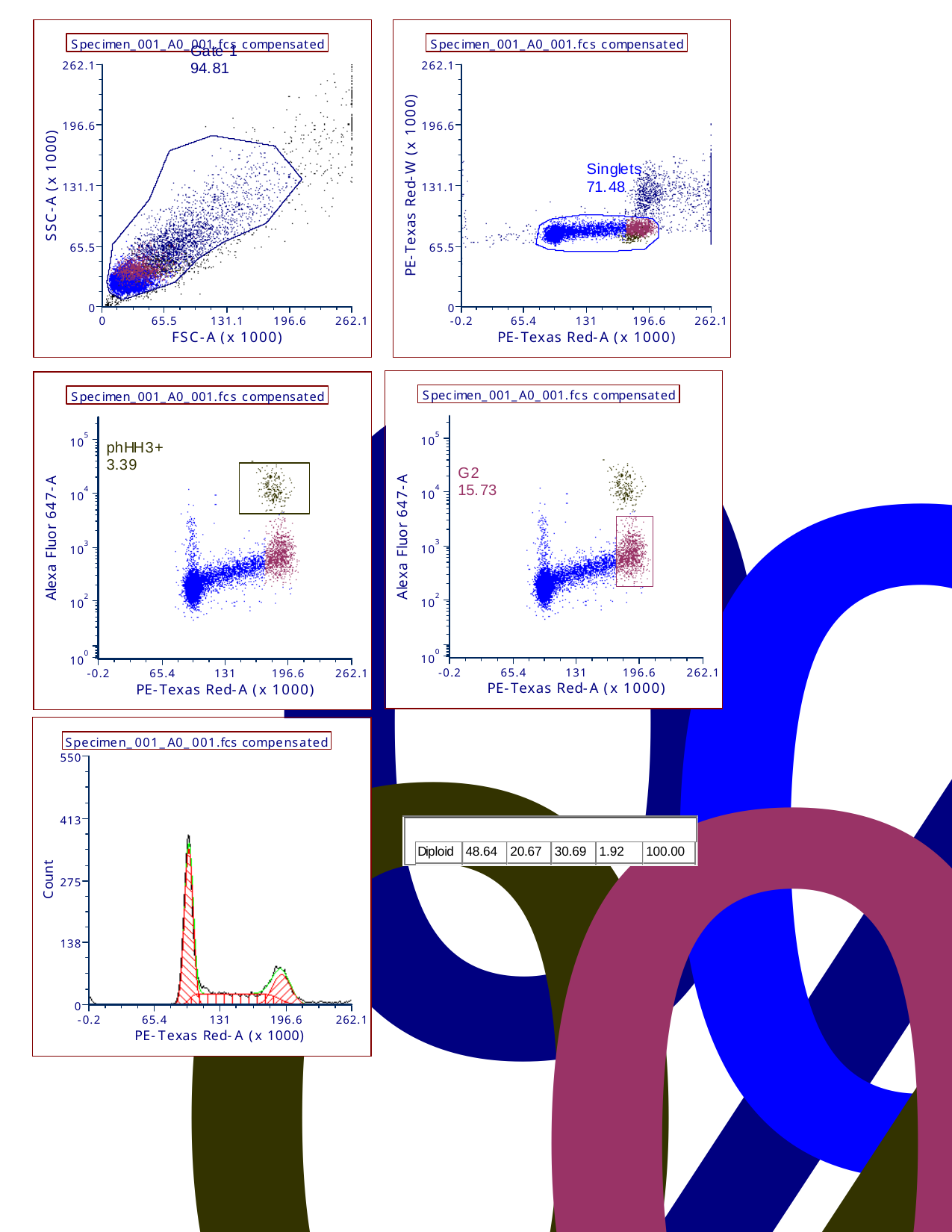

## Slide 2
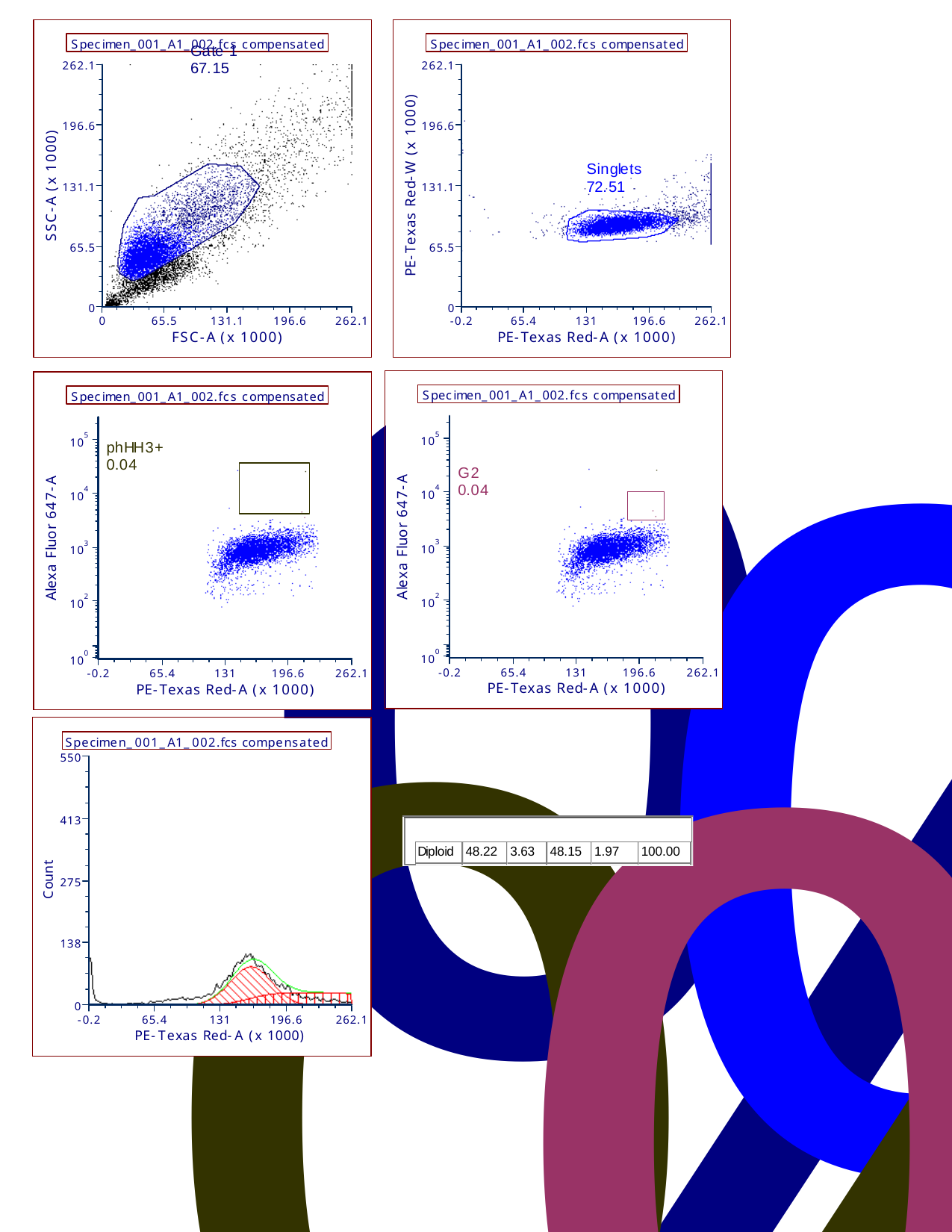

## Slide 3
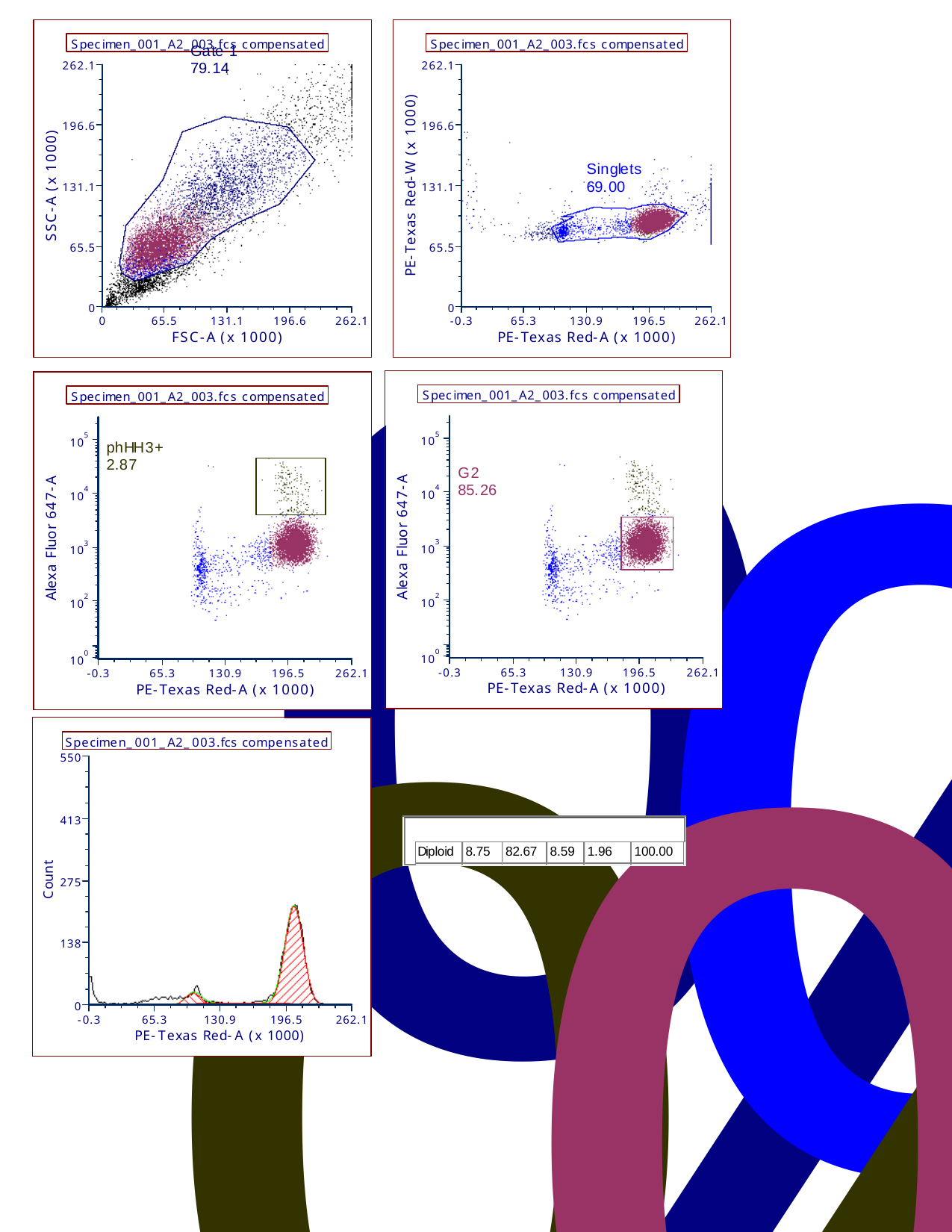

## Slide 4
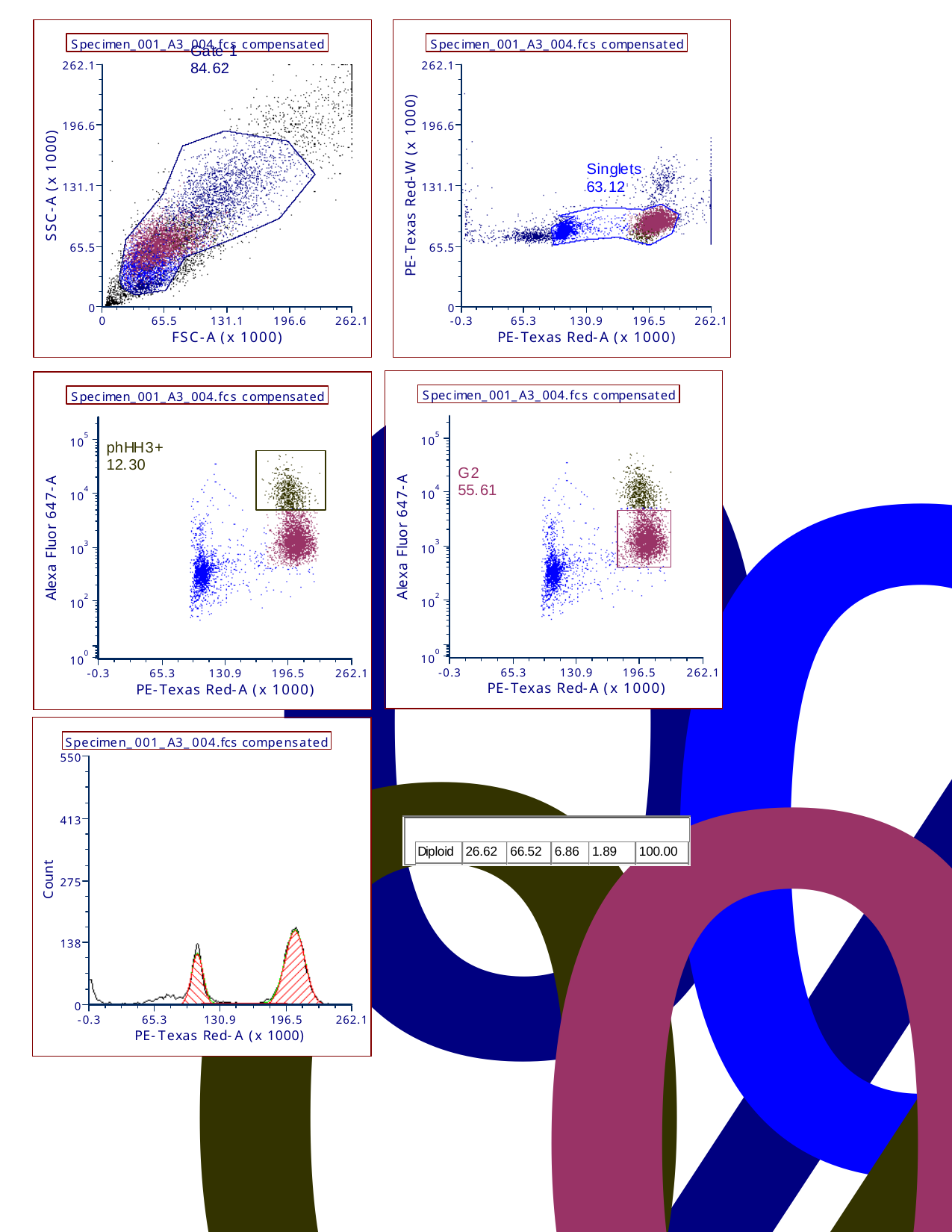

## Slide 5
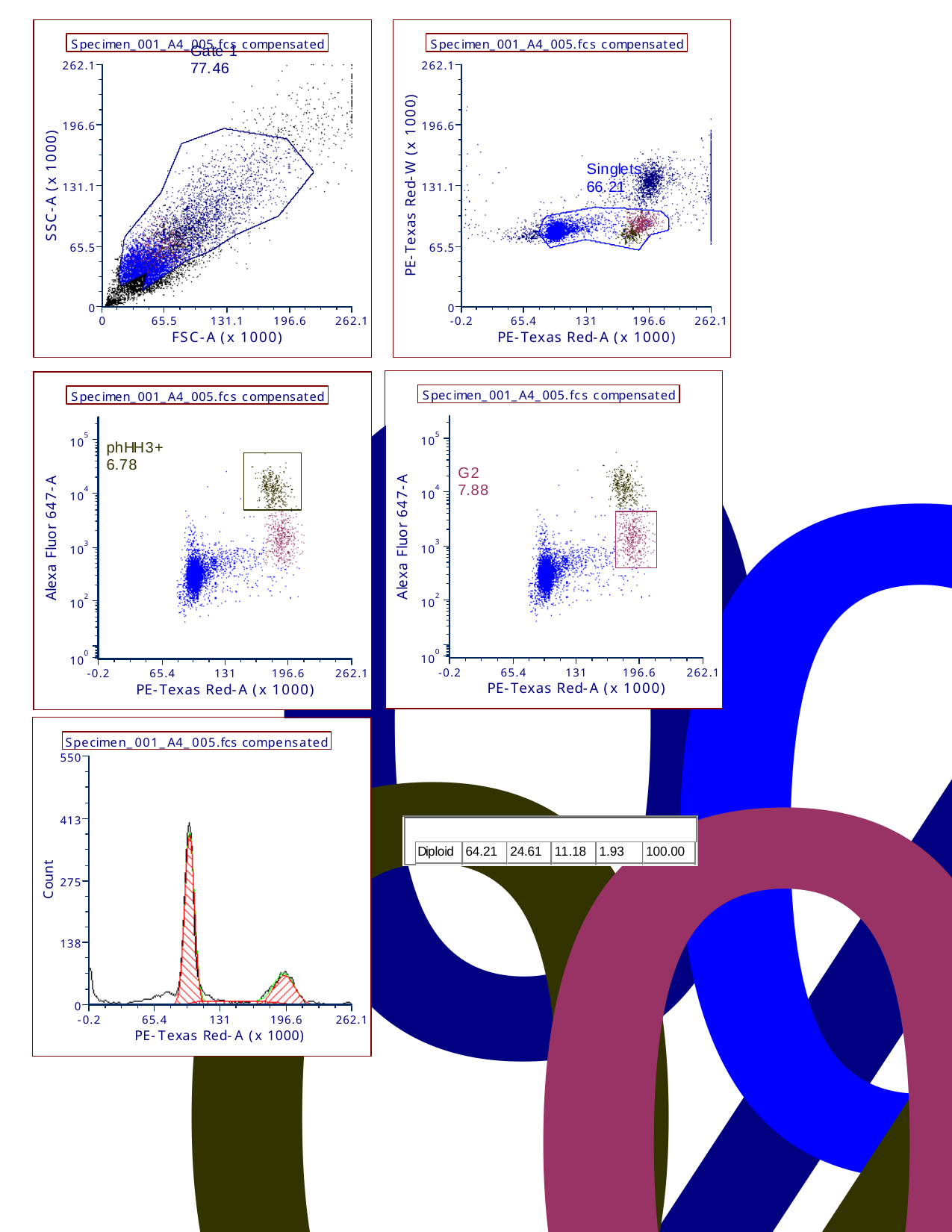

## Slide 6
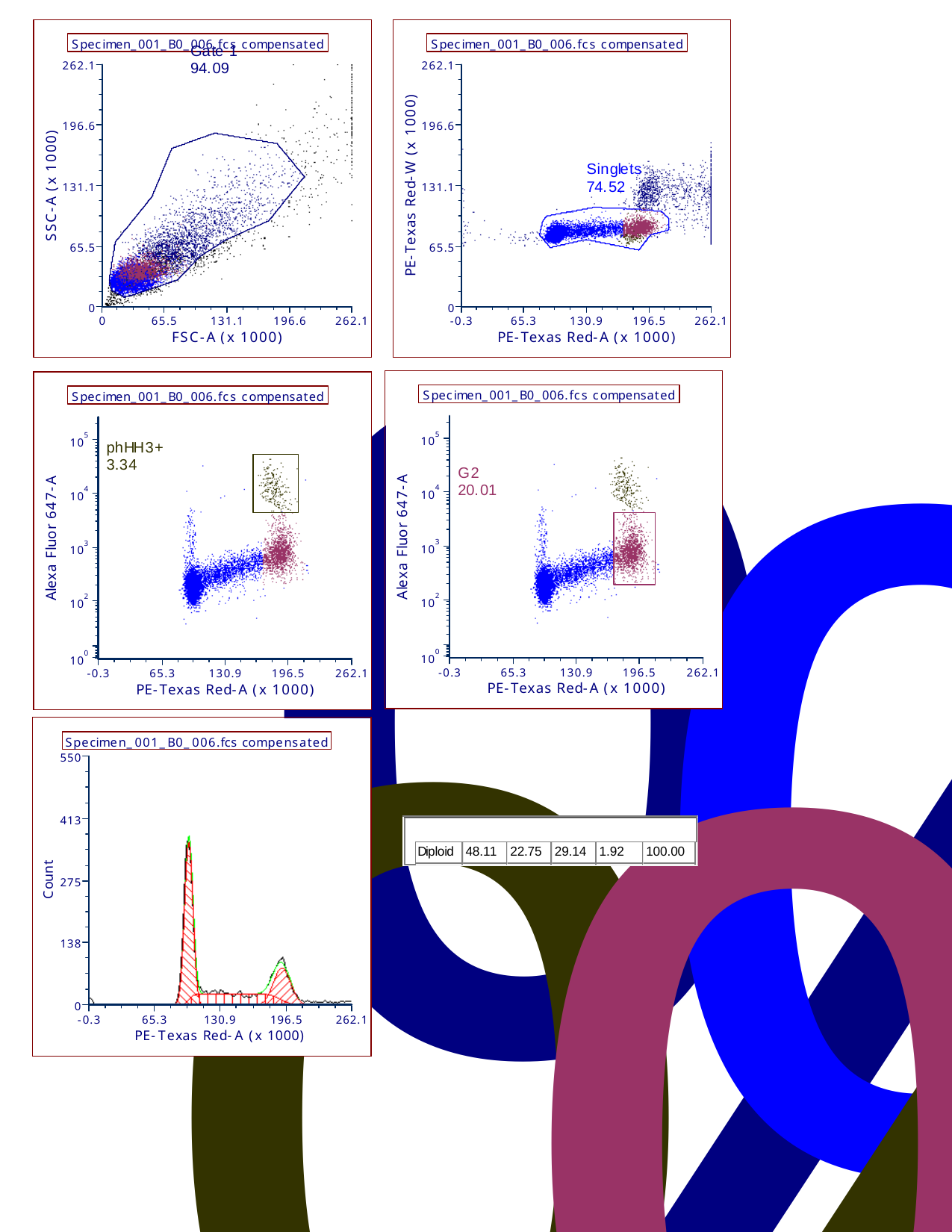

## Slide 7
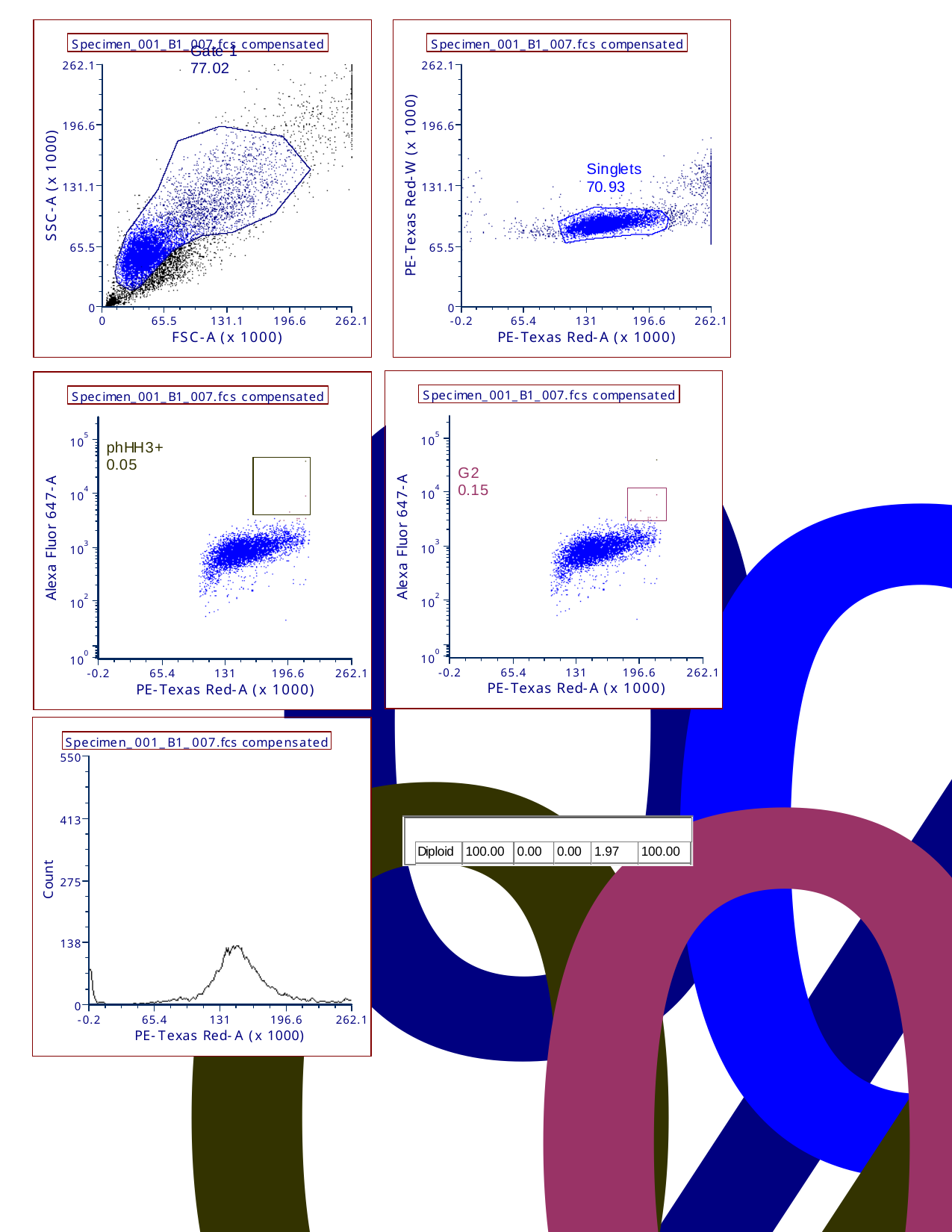

## Slide 8
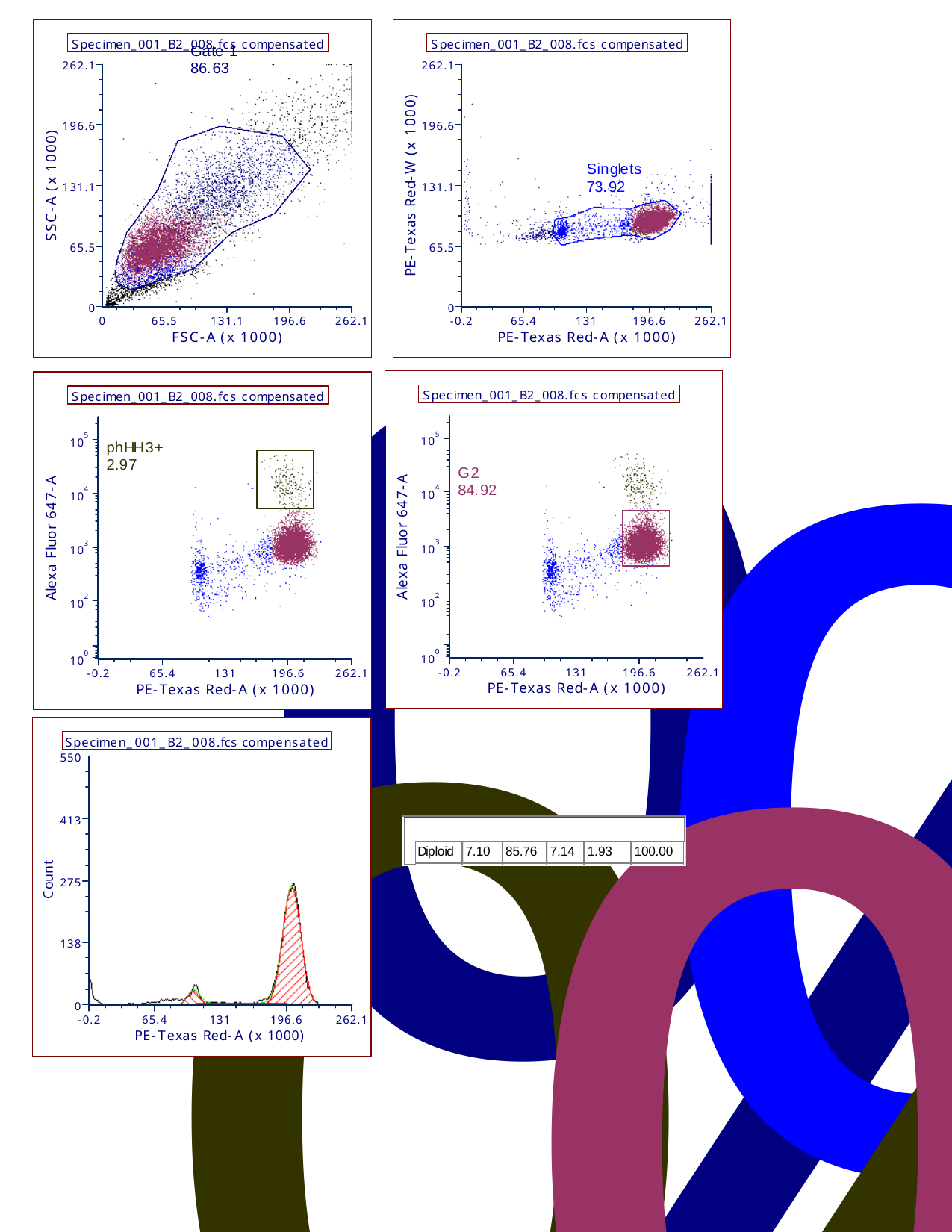

## Slide 9
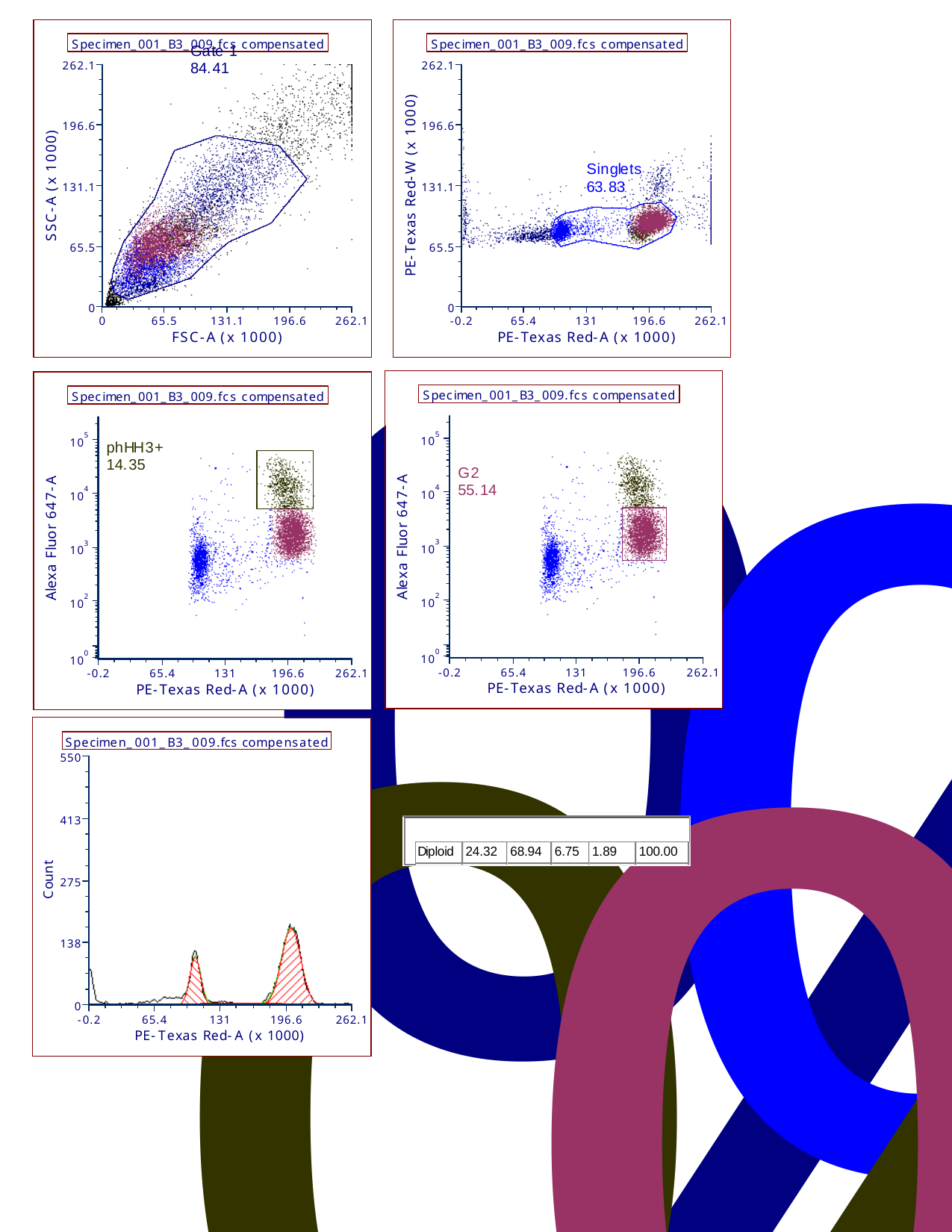

## Slide 10
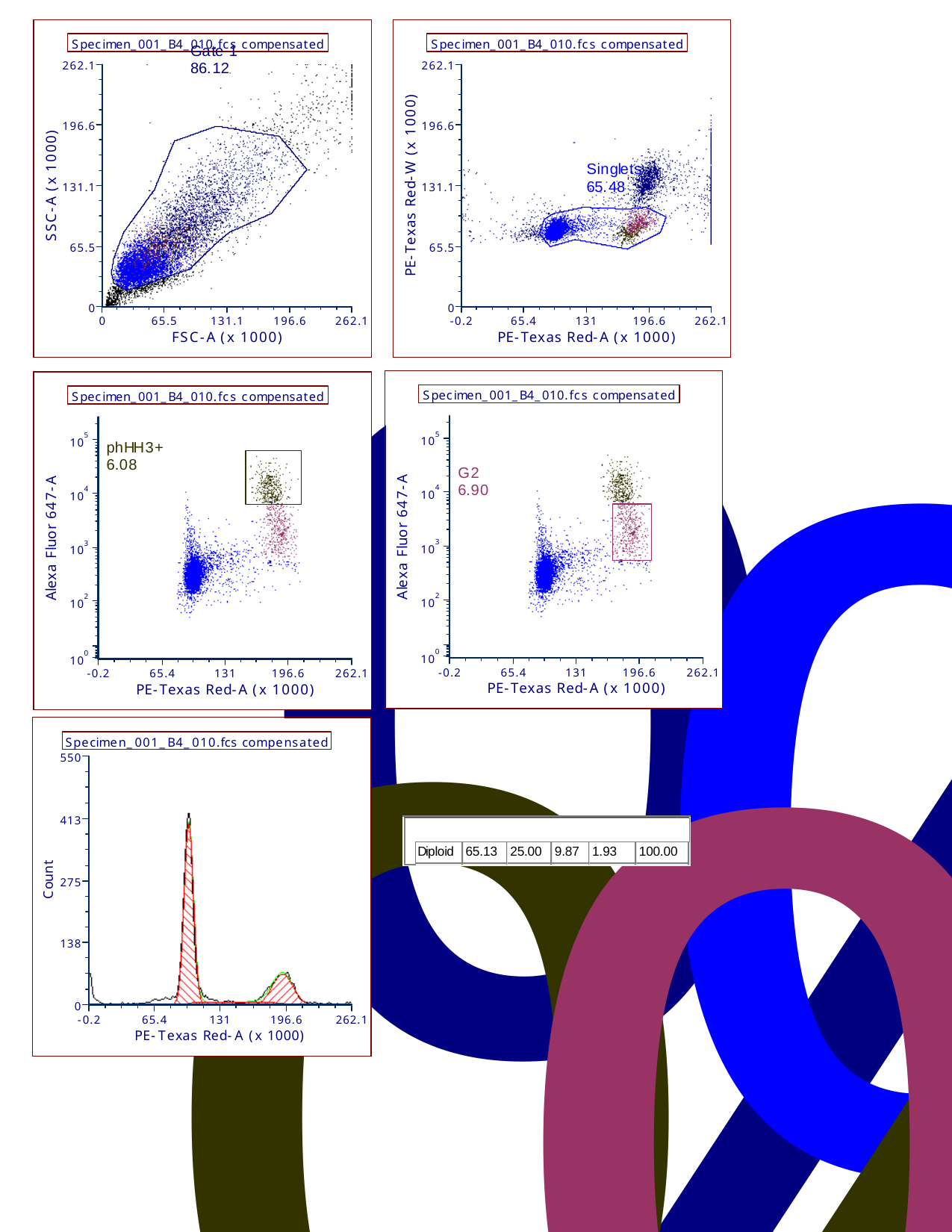

## Slide 11
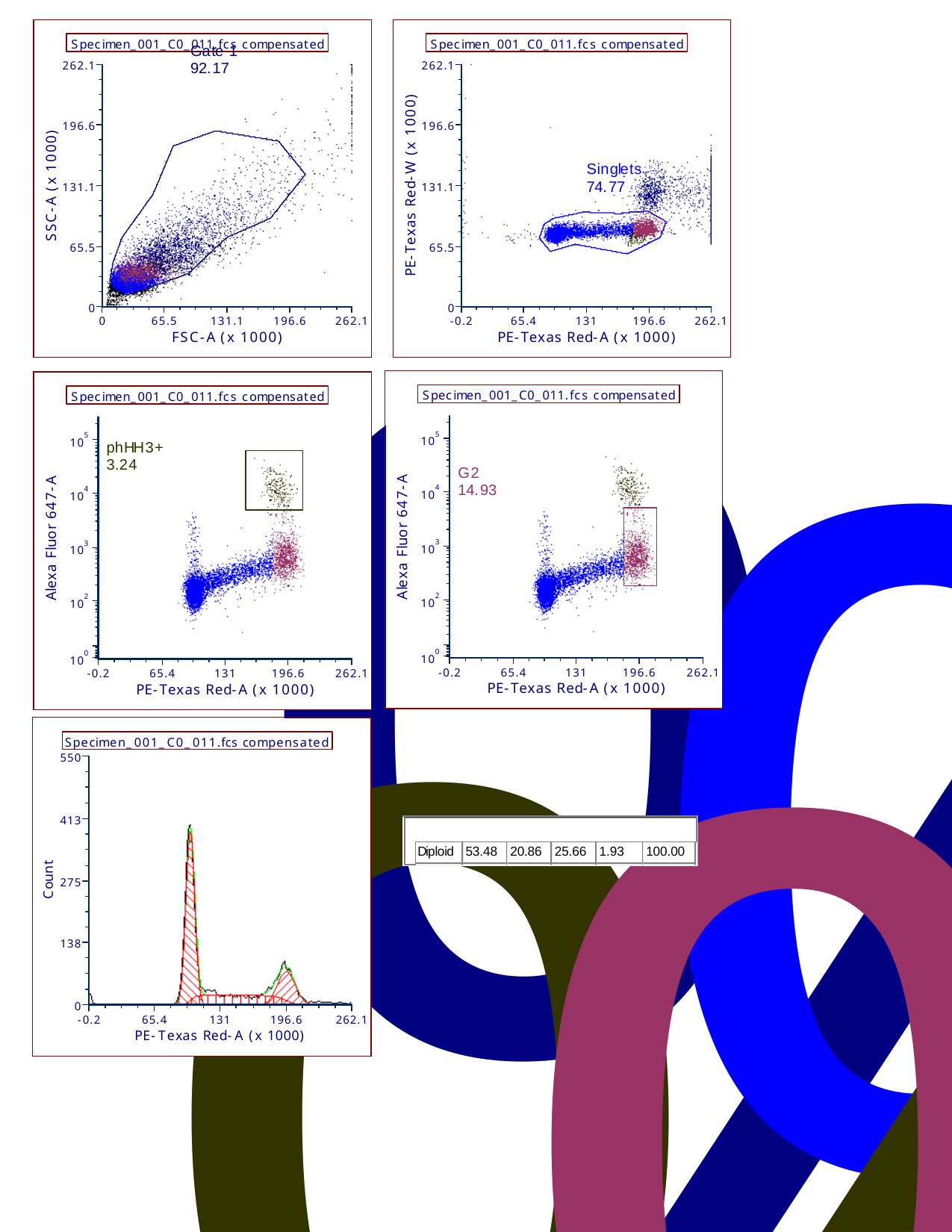

## Slide 12
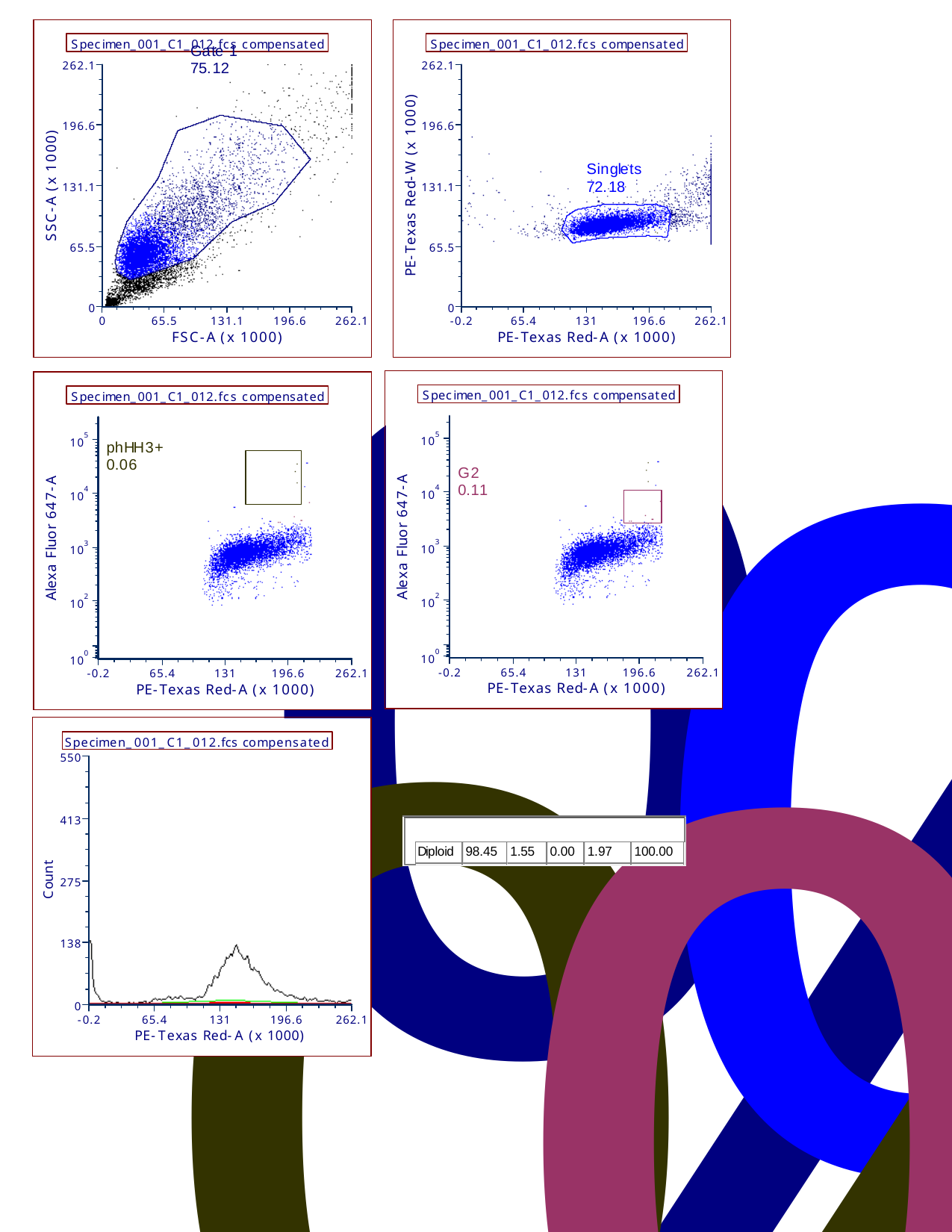

## Slide 13
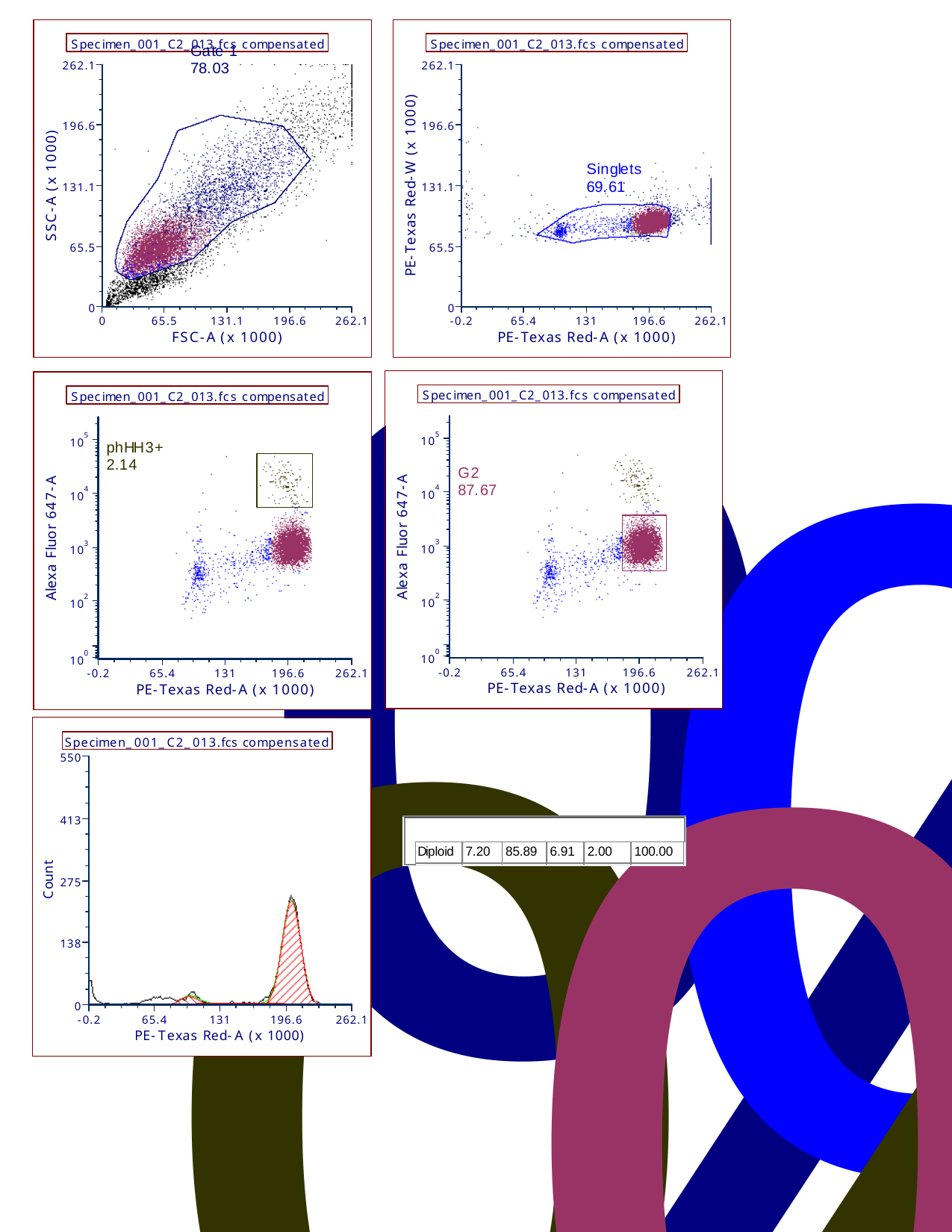

## Slide 14
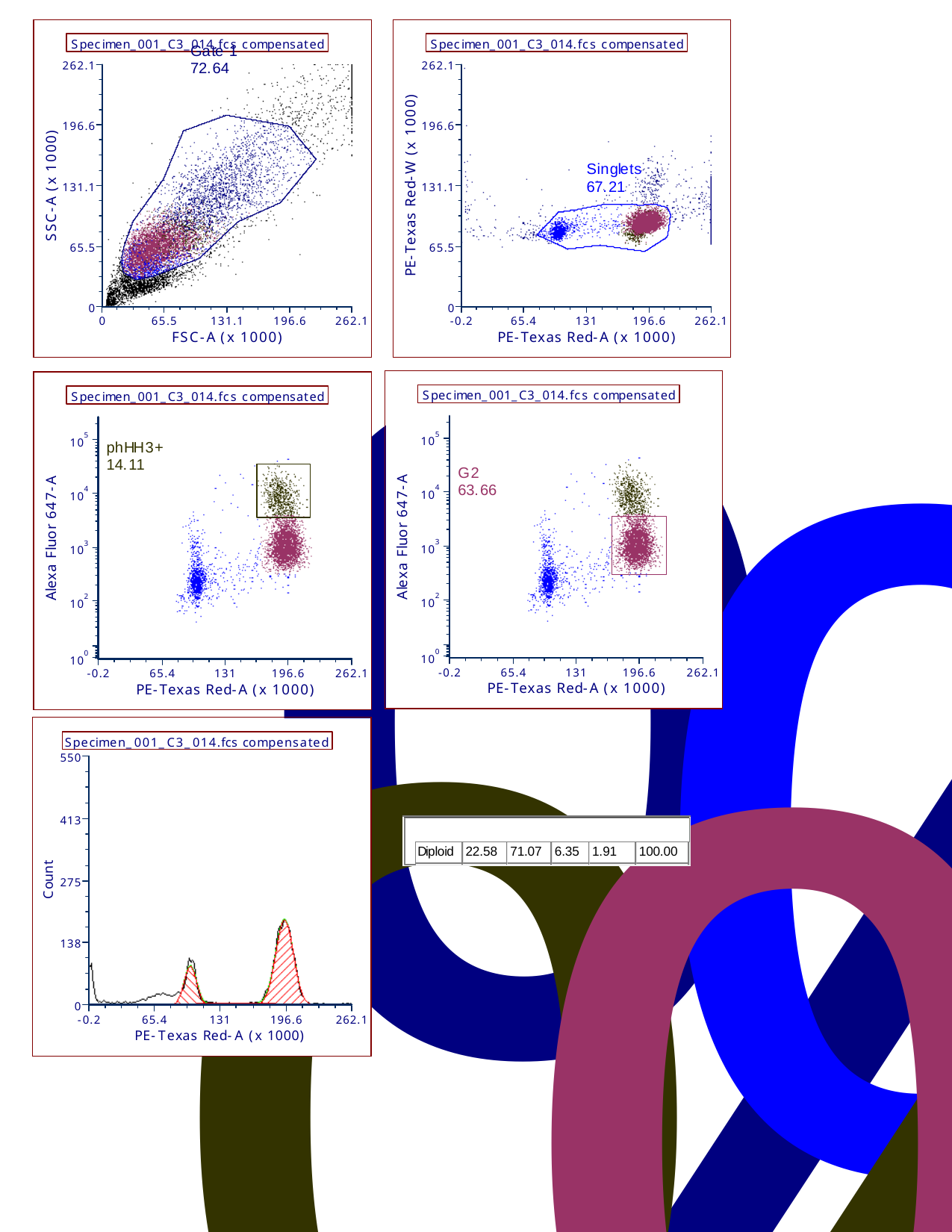

## Slide 15
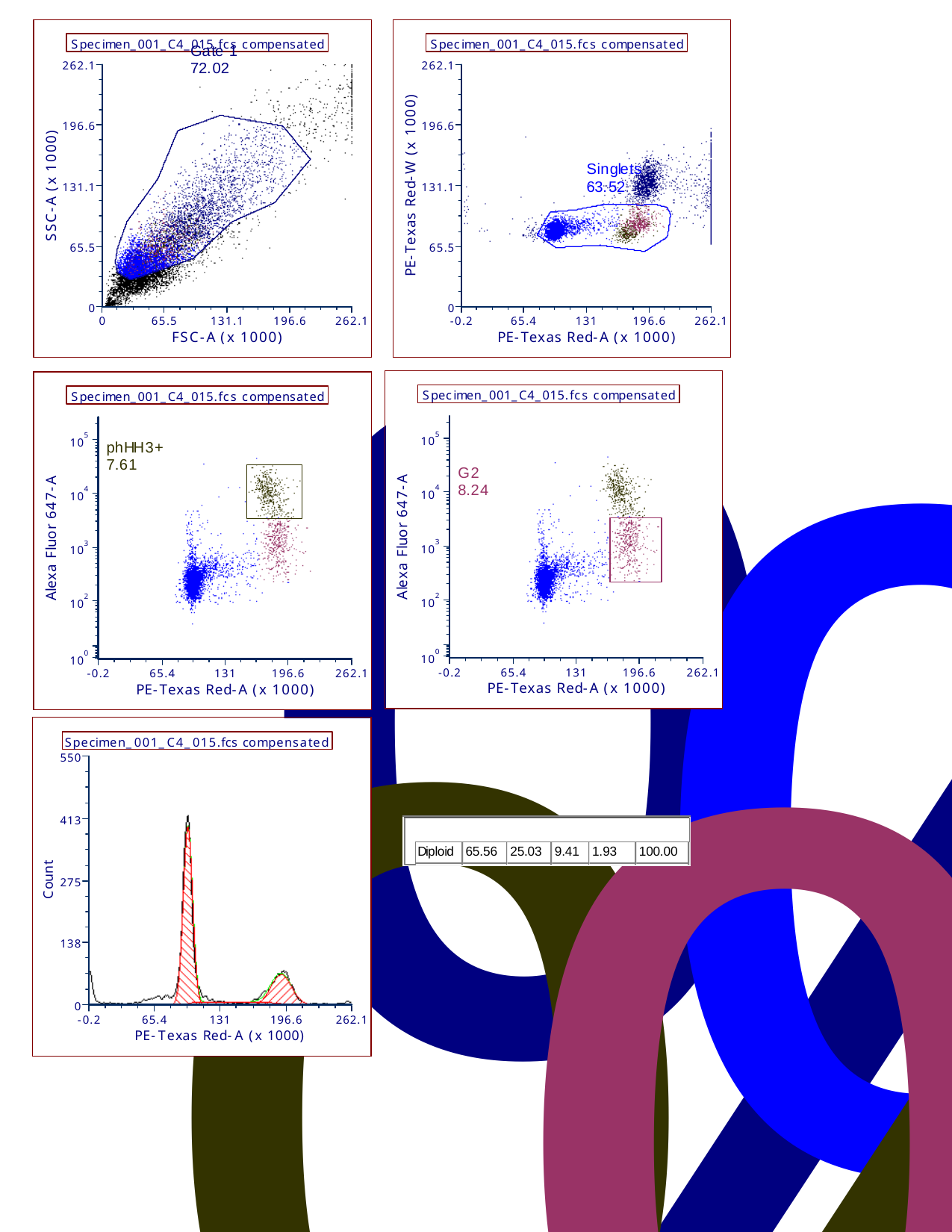

## Slide 16
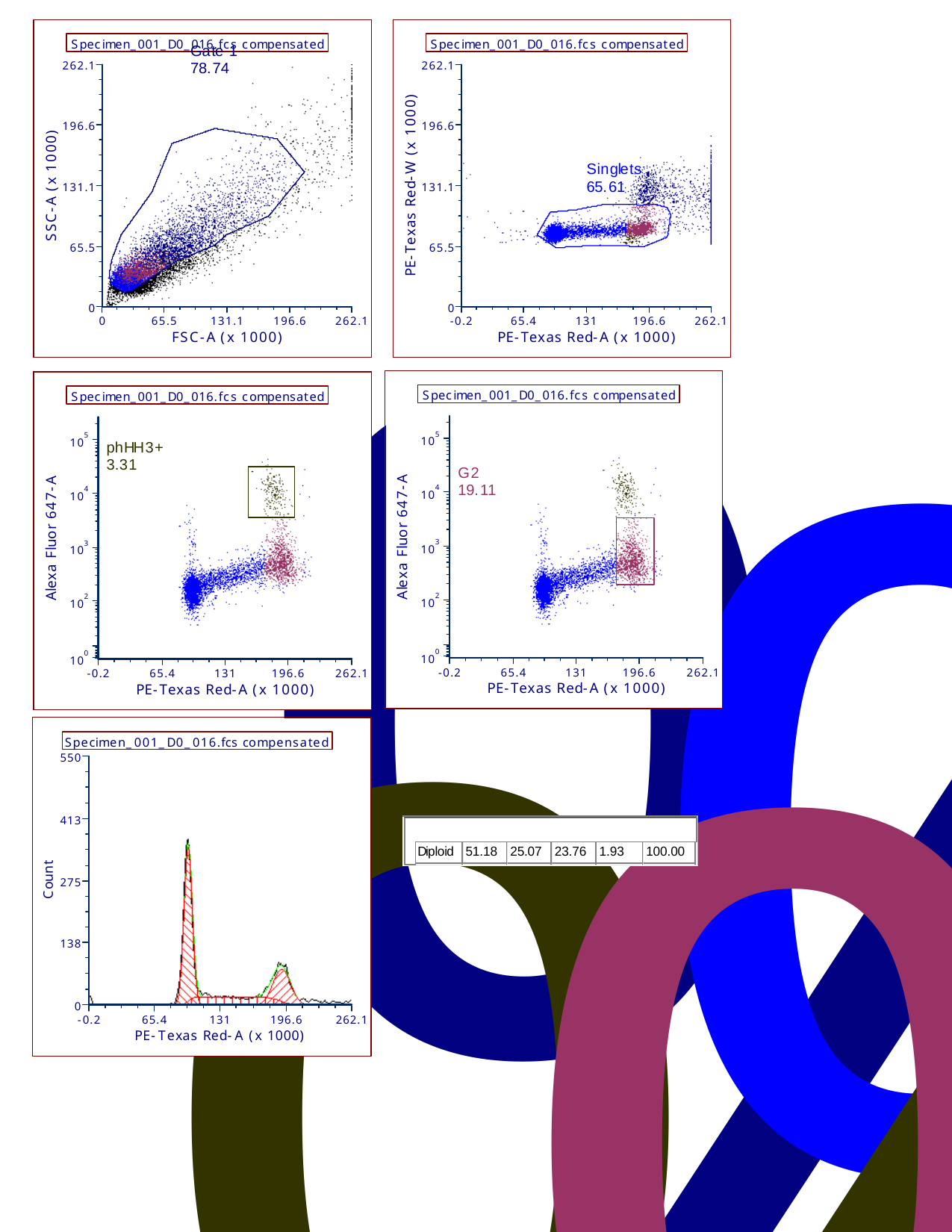

## Slide 17
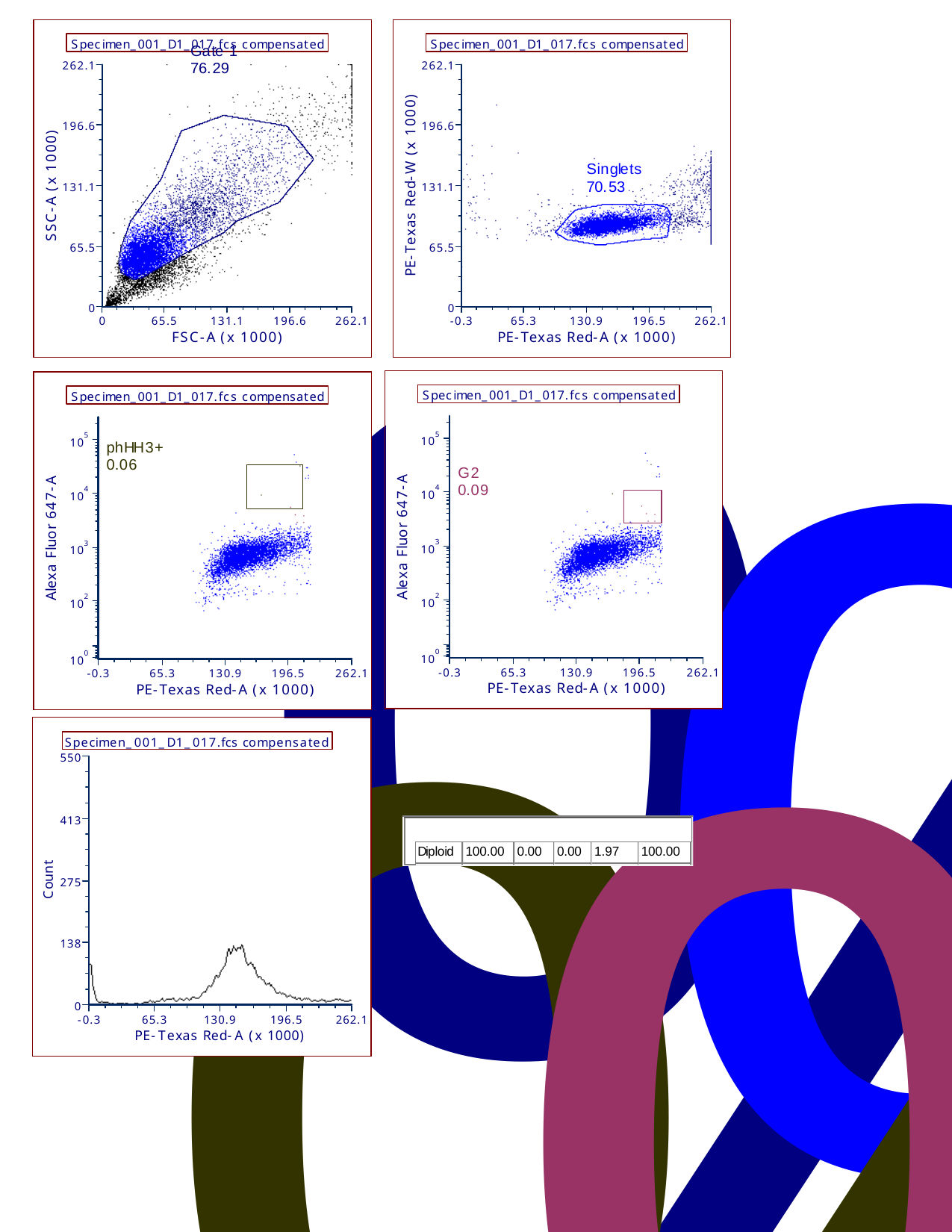

## Slide 18
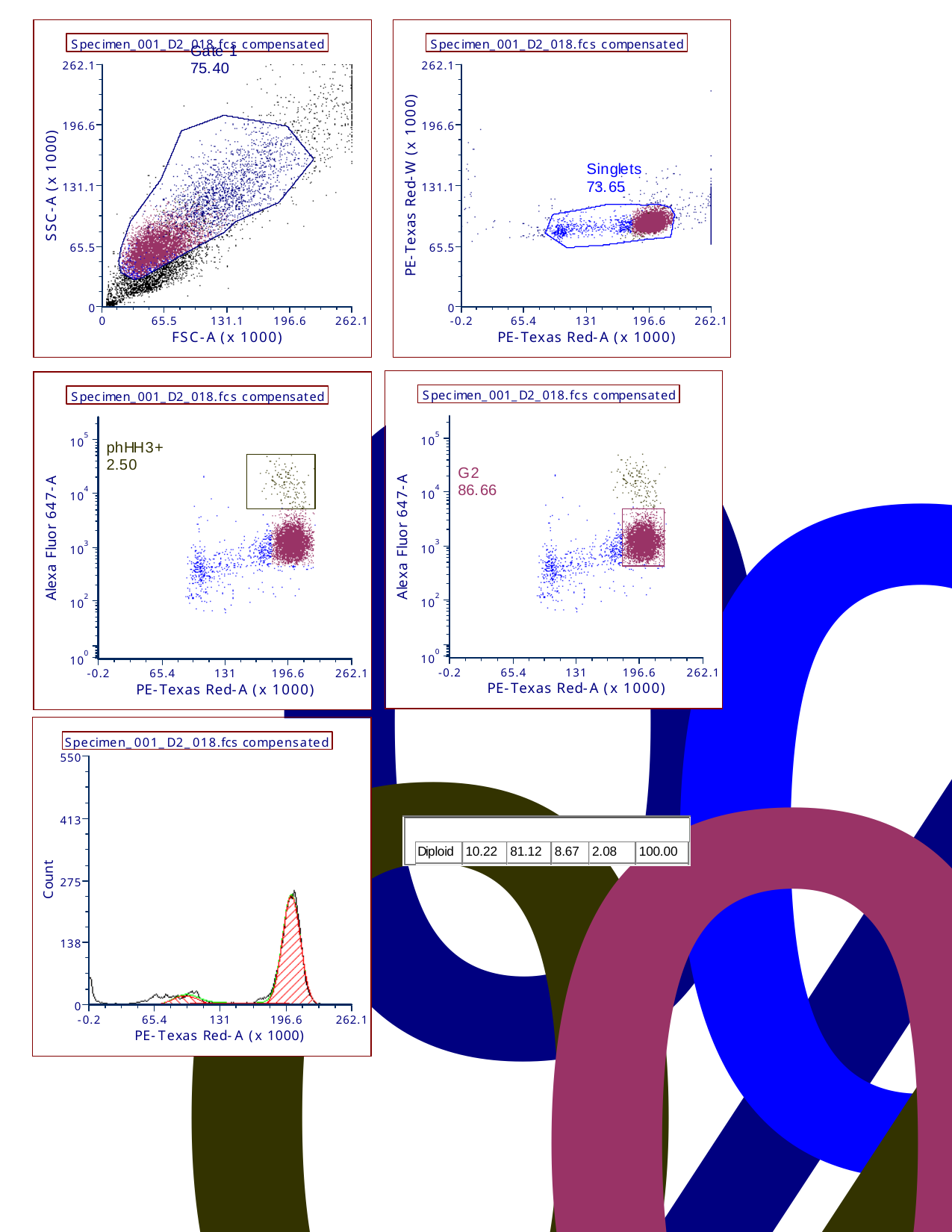

## Slide 19
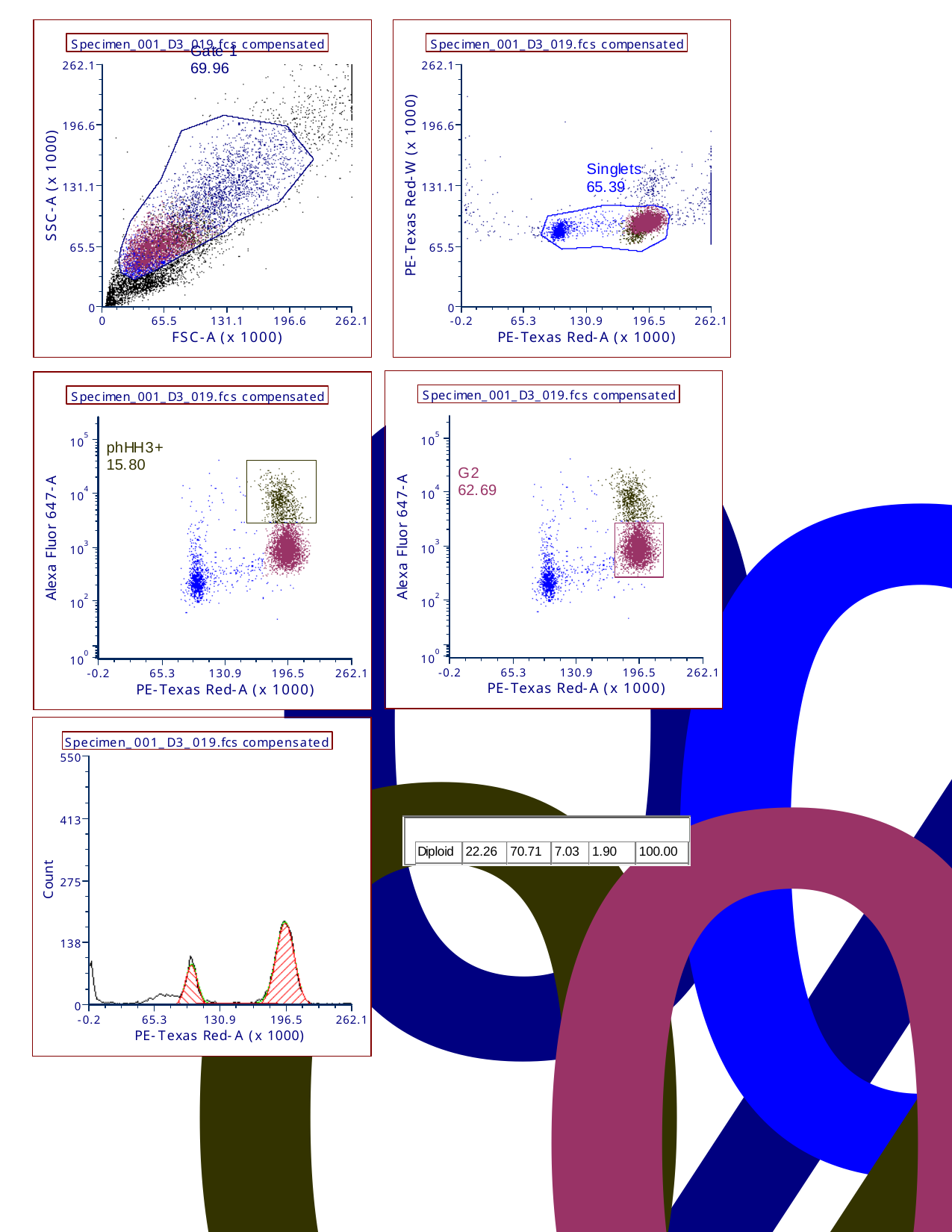

## Slide 20
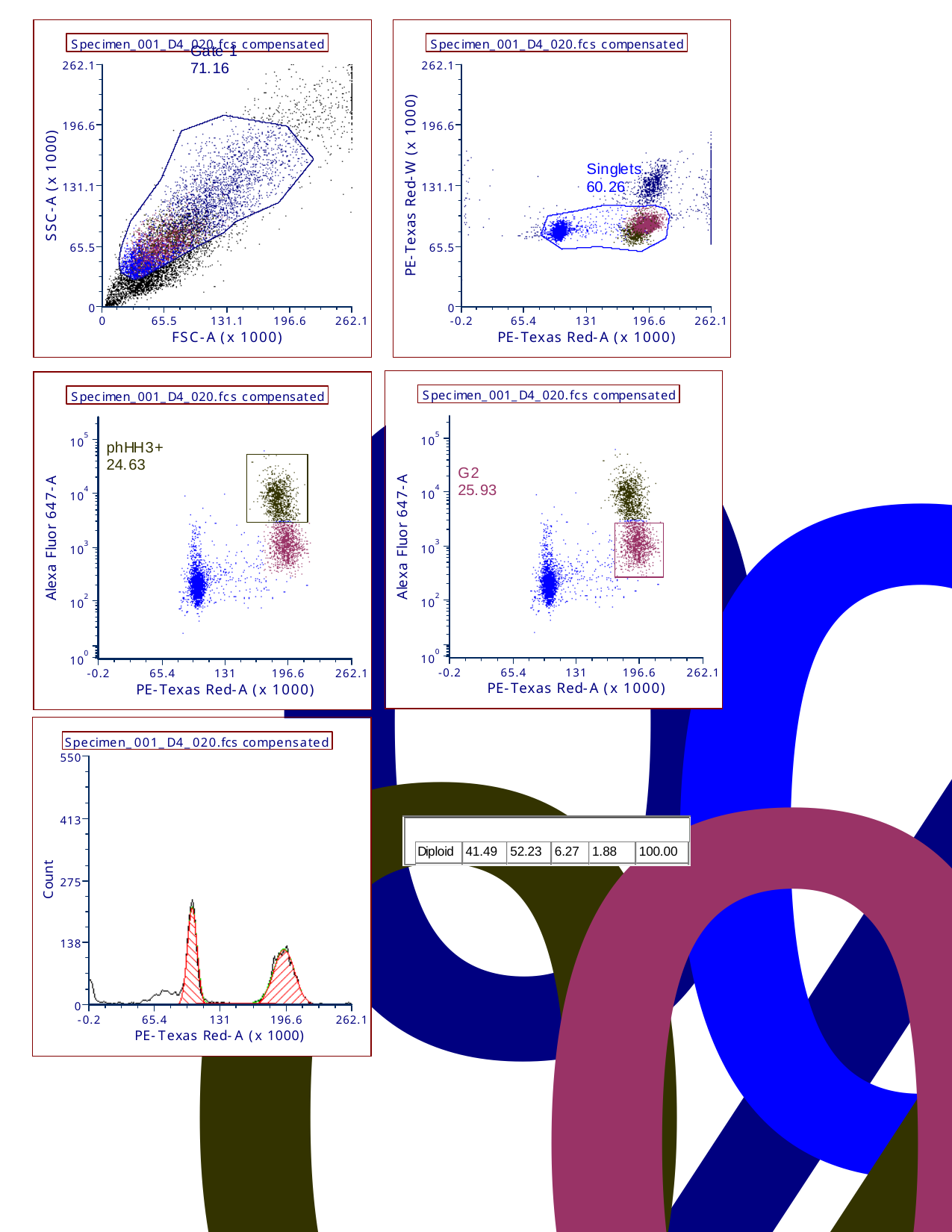

Supplement: Supplementary file 5 — Source Data [file 41467_2022_29502_MOESM5_ESM.zip › Source data/Supplementary_Information_Figure_2.pptx]
